# Methoxyisoflavan derivative from *Trigonella stellata* inhibited quorum sensing and virulence factors of *Pseudomonas aeruginosa*

**DOI:** 10.1007/s11274-022-03337-x

**Published:** 2022-07-08

**Authors:** Nourhan G. Naga, Ahmed A. Zaki, Dalia E. El-Badan, Heba S. Rateb, Khaled M. Ghanem, Mona I. Shaaban

**Affiliations:** 1 Botany and Microbiology Department, Faculty of Science, Alexandria University, Alexandria, Egypt; 2 Pharmacognosy Department, Faculty of Pharmacy, Mansoura University, El Mansûra, 35516 Egypt; 3 Pharmacognosy Department, Faculty of Pharmacy, Horus University-Egypt, New Damietta, 34518 Egypt; 4 Department of Pharmaceutical and Medicinal Chemistry, Pharmacy College, Misr University for Science and Technology, Cairo, Egypt; 5 Microbiology and Immunology Department, Faculty of Pharmacy, Mansoura University, El Mansûra, Egypt

**Keywords:** Quorum sensing inhibition, Virulence factors, Methoxyisoflavan, *Pseudomonas aeruginosa*, *Trigonella stellata*

## Abstract

The number of deaths caused by multidrug-resistant *Pseudomonas aeruginosa* has risen in the recent decade. The development of quorum sensing inhibition (QSI) is a promising approach for controlling *Pseudomonas* infection. Therefore, this study mainly aimed to investigate how a plant-source material inhibits QSI to produce an antipathogenic effect for fighting microbial infections. The QSI effect of *Trigonella stellata* was assessed by using *Chromobacterium violaceum* ATCC 12472 reporter strain. *Trigonella stellata* exhibited high QSI activity, and an ethanolic extract of *T. stellata* was prepared for phytochemical isolation of the most active QSI compound. Nine pure compounds were isolated and identified as kaempferitrin (1), soyasaponin I (2), β-sitosterol-3-*O*-glucoside (3), dihydromelilotoside (4), astrasikokioside I (5), methyl dihydromelilotoside (6), (3R, 4S)-4, 2′, 4′-trihydroxy-7-methoxy-4′-*O*-β-d-glucopyranosylisoflavan (7), (3S, 4R)-4, 2′, 4′-trihydroxy-7-methoxyisoflavan (8, TMF), and (+)-d-pinitol (9). These compounds were screened against *C. violaceum* ATCC 12472, and TMF exhibited a potent QSI. The effect of TMF at sub-minimum inhibitory concentrations (MICs) was assessed against *P. aeruginosa* virulence factors, including biofilm, pyocyanin formation protease and hemolysin activity. TMF induced significant elimination of QS-associated virulence behavior. In addition, TMF at sub-MICs significantly reduced the relative expression of *lasI*, *lasR*, *rhlI*, and *rhlR* compared with that in untreated cells. Furthermore, molecular docking was performed to predict structural basis of the QSI activity of TMF. The study demonstrated the importance of *T. stellata* as a signal modulator and inhibitor of *P. aeruginosa* pathogenesis.

**Supplementary Information:**

The online version contains supplementary material available at 10.1007/s11274-022-03337-x.

## Introduction

*Pseudomonas aeruginosa* is an opportunistic human pathogen that can be isolated from various ecosystems, including water and soil (Baltch and Smith 1994). *Pseudomonas aeruginosa* is prevalent in people who have cystic fibrosis, serious burns, or deep-wound infections, as well as those who have urinary tract infections (Turkina and Vikström 2019), and is a problematic pathogen due to the development of multiple drug resistance that extends to many antimicrobial agents. In addition, *P. aeruginosa* can form a biofilm that acts as a protective shield containing extra polysaccharide layers that prevent the penetration of antimicrobial agents. These factors create an urgent need for the development of novel approaches to manage *Pseudomonas* infection (Tolker‐Nielsen 2014).

*Pseudomonas aeruginosa* exhibits various virulence behaviors, including extracellular molecules such as rhamnolipids, lectins, lipase and aminopeptidase that facilitate microbial spreading. Furthermore, *P. aeruginosa* exhibits a pronounced ability to colonize hosts via adhesive microbial attachment and biofilm assembly. The majority of these virulence behaviors are regulated by quorum sensing (QS; Jimenez et al. 2012; Maraolo et al. 2020). QS is a cell-to-cell communication process mediated by the production of small molecules called autoinducers (AIs). These AIs function as infochemicals enabling bacterial cells to act as a group and facilitating crosstalk between two distinct bacterial species that share the same environment. Currently, four basic QS circuits in *P. aeruginosa* have been identified: LasI/LasR and RhlI/RhlR (Pesci et al. 1997), *Pseudomonas* quinolone signal (PQS) system (Dubern and Diggle 2008), and integrated QS system (Rampioni et al. 2016). On reaching a threshold level, these chemicals form a network that is interconnected and allows hierarchical control of each other’s activities and thereby controls the expression of genes linked to virulence factors (Passador et al. 1993).

QS inhibition (QSI) is one of the most promising approaches for reducing the pathogenicity of *Pseudomonas* infection via various mechanisms (Rémy et al. 2018; Naga et al. 2021). Plants are a potent source of therapeutic compounds and have attracted attention for as potential sources of compounds that could interfere with the QS signaling systems of *P. aeruginosa* and reduce associated pathogenesis. Natural products have long been thought of as a source of essential antibacterial agents that could be used to treat various pathogenic infections (Howes et al. 2020). For example, ascorbic acid is a purely natural compound that has been found to be a QSI (El‐Mowafy et al. 2014). In addition, QSIs have been identified from extracts of medicinal plants, including *Adhatoda vasica* Nees, *Myoporum laetum* (Zaki et al. 2013), *Citrus sinensis*, *Laurus nobilis*, *Elettaria cardamomum*, *Allium cepa*, and *Coriandrum sativum* (Al-Haidari et al. 2016)*.*

*Trigonella stellata* is a palatable and nutraceutical herb that possesses anti-inflammatory activity, antioxidant and anticancer effects (Sindhu et al. 2012; Sheweita et al. 2020) and has been used as a traditional treatment for diarrhea and dysentery. The methanol extract of *Trigonella foenum-graceum* (fenugreek) seeds inhibited QS and biofilm formation in *Aeromonas hydrophila* and *P. aeruginosa* with activity associated with caffeine production by the plant (Husain et al. 2015). However, the antivirulence and the QSI activity of *T. stellata* have not yet been assessed. For instance, *T. stellata* could be a promising source for active derivatives that have potential QSI efficacy and antivirulence attributes against *P. aeruginosa*.

Therefore, this study aimed to evaluate the QSI effect of *T. stellata* total extract followed by purification and identification of active compounds. We evaluated the anti-QS activity of the pure compounds against *P. aeruginosa* clinical isolates and then assessed the phenotypic and genotypic analysis of QSI and potential for inhibition of virulence traits. Finally, we performed a modeling study to identify the probable mechanism of QSI in respect to an identified active component.

## Materials and methods

### Plant material collection and extract preparation

*Trigonella stellata* aerial parts were gathered from Daba, Matrouh, Egypt, in April 2013. The plant was identified by the Botany Department, Faculty of Sciences, Mansoura University. A voucher specimen with the code “TsT-4-13” was washed in tap water directly after collecting, air-dried for 6 weeks, and grounded using a miller to fine powder. Then, 50 g of dried powder was extracted using 70% ethanol and incubated overnight at 30 °C with shaking at 200 rpm. Plant extracts were filtrated and concentrated using a rotary evaporator at 40 °C.

### Bacterial strains and growth media

The QSI activity was assessed using the standard strain *C. violaceum* ATCC 12472 (McClean et al. 1997), which was grown for 24 h at 28 °C on Luria–Bertani (LB) media (0.5% w/v yeast extract, 1% w/v tryptone, and 1% w/v NaCl, pH 7) and 2% w/v agar (Bertani 2004). *Pseudomonas aeruginosa* clinical strains were collected from urine samples and termed Ps.A11, Ps.A12, Ps.A13, and Ps.A16. Collected samples were handled in accordance with the ethical guidelines of the Faculty of Medicine, Alexandria University, Egypt, and were identified as *P. aeruginosa* according to biochemical standards (Koneman et al. 2006) and the Vitik2 (bioMerieux, Inc., UAS) automated identification method. In addition, *P. aeruginosa* PA14 and PAO1 were used as the positive standard strains (Maura and Rahme 2017), while *P. aeruginosa* PAO-JP2 was used as a negative standard strain (Pearson et al. 1997). Standards and clinical *P. aeruginosa* strains were grown in LB medium and incubated overnight at 37 °C.

### QSI assay of crude plant extract

Pure colonies of *C. violaceum* ATCC 12472 were inoculated in LB broth medium and cultivated at 28 °C with shaking at 200 rpm. Sterile LB agar molten at 55 °C was prepared and 15 mL was poured in the plates and left to solidify; the soft LB agar (5 mL) was then inoculated with 100 µL *C. violaceum* ATCC 12472 culture, and plates were allowed to completely solidify before wells were cut (10-mm diameter) in the agar using a cork borer. An aliquot of the concentrated *T. stellata* extract (100 μL) was added to the agar wells. After 24-h incubation at 28 °C, the extent of violet color around the well was evaluated and inhibition of this was determined as a positive QSI activity. Solvent was used as a negative control (McClean et al. 1997).

### Isolation of the active compounds of *T. stellata*

A total of 470 g of air-dried and crushed aerial portions of *T. stellata* was extracted with 3 L of ethanol till exhaustion for 48 h at room temperature. The ethanolic extract was collected and dried at 45 °C under vacuum to yield a dry total extract with a final weight of 15 g. The entire dried extract was then disseminated in 250 mL water using sonication and then fractionated with hexane, ethyl acetate, methylene chloride, and butanol. The hexane, methylene chloride, ethyl acetate, and butanol fractions were dried to obtain weights of 3.9, 1.1, 1.53, and 2.5 g, respectively.

The ethyl acetate fraction (1.53 g) was spotted onto a 2.5 × 95 cm silica gel column and eluted with a mobile phase consisting of ethyl acetate:CHCl_3_:CH_3_OH:H_2_O at ratios of 15:8:4:1, 10:6:4:1, and 6:6:4:1; eluates were then examined using thin-layer chromatography (TLC). The subfractions of comparable spots were combined to yield eight fractions (TE1 to TE8). Fraction TE2 (185 mg) was chromatographed over RP-18 column and isocratically eluted with CH_3_OH:H_2_O (1:1) to provide two pure compounds: 6 and 7 (13.2 and 9.7 mg, respectively).

The methylene chloride extract (1.1 g) was purified using vacuum liquid chromatography (VLC) over RP-18 silica (20 × 2 cm) and eluted with two gradients of CH_3_OH:H_2_O (1:1 then 6:4) to obtain 11 fractions (TC1 to TC11). Fraction TC5 (118 mg) was applied to a standard silica gel column (2 × 95 cm) and eluted with ethyl acetate:CHCl_3_ (1:1) followed by ethyl acetate:CHCl_3_:CH_3_OH:H_2_O (10:6:4:1, then 6:4:4:1) to provide the purified compound 8 (10 mg). Fraction TC9 (529 mg) was chromatographed over a silica gel glass column (1.5 × 90 cm) and eluted with the gradients of ethyl acetate:CHCl_3_:CH_3_OH:H_2_O (15:8:4:1, 10:6:4:1, and 6:4:4:1) to yield compound 3 (23 mg).

The butanol extract (2.5 g) was passed over a VLC packed with RP-18 silica (25 × 4 cm) and eluted with the following mobile phase compositions H_2_O:CH_3_OH (1:0, 9:1, 7:3, 6:4, 5:5, 4:6, 3:7, 2:8, and 0:1), 1 L each, to yield 11 fractions (TB1 to TB11). Compound 9 (31 mg) was precipitated from the methanol-concentrated fraction TB4 (179 mg). The purification of two compounds 1 (8.1 mg) and 4 (9.8 mg) was achieved through column chromatography of fraction TB2 (108 mg) over RP-18. A white powder of purified compound 2 fraction TB6 (182 mg) was applied to an RP-18 column (2 × 30 cm) and eluted with gradients of H_2_O:CH_3_OH (1:1, 6:4, and 4:6). Compound 5 (6.2 mg) was obtained through the RP-18 column fractionation of TB7 (110 mg) and isocratic elution with H_2_O:CH_3_OH (6:4).

### Characterization of isolated compounds by NMR spectroscopy

One- and two-dimensional (1D and 2D) NMR experiments were performed using a Varian 400 MHz, Bruker DRX-500, or Bruker Avance III 600 and 400 MHz spectrometer with deuterated solvents of pyridine, methanol, or dimethylsulfoxide (DMSO); the solvent signals served as an internal reference for adjustment. The Agilent Technologies 6200 series was used to record the positive and negative ion modes of compound masses. An AUTOPOL IV Automatic Polarimeter (Rudolph, Hackettstown, NJ, USA) was used to detect the specific rotations of the pure compounds. In addition, the Varian Cary 50 Bio UV–Vis spectrophotometer was used to measure the UV spectra. The silica gel (particles diameter 32–63 µm, Dynamic Adsorbents, Inc.) and RP-18 (Polar bond, J. T. Baker) were used for the preparation of column chromatography. TLC plates were monitored with silica gel F254 sheet (Fluka) or RP-18 (Merck). The spots of the tested compounds were visualized at 254 nm (UV) and then sprayed with vanillin (1% w/v) (Sigma) prepared in concentrated H_2_SO_4_:C_2_H_5_OH (10:90). All the chromatographic procedures were performed using analytical grade solvents (Fischer Chemical).

### Assay of QSI activity of *T. stellata* isolated compounds with *C. violaceum* ATCC 12472

QSI activity of the nine isolated compounds from *T. stellata* was assessed using *C. violaceum* ATCC 12472. Compounds were dissolved to 5 mg/mL in DMSO. Aliquots of 50 μL of each compound were applied in each well (10 mm diameter), and then the plates were then incubated at 28 °C. After 24 h, the inhibition of induced violacein production around the wells was examined. DMSO was used as a control. Compounds possessing potential QSI with inhibition of violacein pigment production were estimated for their effect on virulence factors.

### Antibacterial screening

The minimum inhibitory concentration (MIC) of the active compound TMF was calculated using the microtiter plate assay technique. Each well received 100 µL Mueller Hinton broth, and the TMF compound was serially diluted in twofold serial concentrations (512, 256, 128, 64, 32, 16, 8, 4, 1, and 0.5 µg/mL) and inoculated with 1 × 10^6^ CFU/well of actively growing *P. aeruginosa* strains (Patel et al. 2015). The negative control consisted of wells containing media, whereas the positive control included wells containing media inoculated with the test strains. The MIC was defined as the lowest concentration of TMF compound that inhibited growth.

### Effect of sub-MIC of TMF on the growth of *P. aeruginosa* strains

The viability of *P. aeruginosa* was investigated using the pour plate method after adding 1/2 MIC of TMF and comparing with that of the untreated culture. A volume of the overnight culture (1 mL) was collected and diluted 1:10. Diluted cultures were subcultured in molten LB agar, and the colonies were counted after overnight incubation at 37 °C. *Pseudomonas aeruginosa* growth curves (treated and nontreated) at an optical density (OD) at 600 nm were estimated at different time intervals.

### Antivirulence effect of TMF on *P. aeruginosa*

Different virulence factors of *P. aeruginosa* strains were quantified after treatment by 1/2 and 1/4 MICs of TMF in triplicates. Assessment of virulence factors was performed with and without TMF (El-Mowafy et al. 2017). Similarly, control strains PAO1 and PA14 (positive controls) and PAO-JP2 (negative control) were measured under the same conditions (Musthafa et al. 2012).

### Biofilm formation assay

*Pseudomonas aeruginosa* biofilm formation was assessed using 96-well flat-bottomed polystyrene plates. Aliquots of the treated and untreated cultures (100 μL) were distributed into the plates, which were then incubated at 37 °C for 24 h. Wells were washed three times with 200 μL of physiological saline before the attached bacteria were fixed for 15 min with 150 μL of absolute methanol. After emptying and drying the plates, the bacterial cells were stained with 150 μL of 1% (w/v) crystal violet. The excess stain was washed away, and the dye was dissolved in glacial acetic acid at 33% (v/v). The absorbance of the dyed cells was determined at 490 nm (Adonizio et al. 2008).

### Pyocyanin assay

Pyocyanin was quantified using King A broth media (peptone 2% (w/v), K_2_SO_4_ 1.0% (w/v), and MgCl_2_ 0.14% (w/v)) (Essar et al. 1990). A volume of 500 μL of *P. aeruginosa* overnight culture was added to 5 mL of King A broth (treated and untreated media) and incubated with agitation at 200 rpm at 37 °C. The cultures were then centrifuged at 3000 rpm for 10 min at 4 °C to remove the cells. Pyocyanin was extracted with 3 mL of chloroform and vortexed until the color changed to greenish blue. The mixture was centrifuged at 3000 rpm for 10 min; 1 mL of 0.2 M HCl was added, and the mixture was shaken until the blue color turned pink. The absorbance of the pink layer was measured at 520 nm, and the concentration of pyocyanin in µg/mL was estimated using the formula: absorbance: absorbance × 17.072 (Essar et al. 1990).

### Determination of hemolysin activity

The hemolytic activity of *P. aeruginosa* untreated and treated (1/2 and 1/4 MICs of TMF) culture-free supernatant was determined. In brief, 500 μL of supernatant was mixed with 700 μL of washed erythrocytes and incubated for 2 h at 37 °C. The reaction mixture was centrifuged at 8000 × *g* for 15 min at 4 °C (Rossignol et al. 2008), and the absorbance was measured at 540 nm (Annapoorani et al. 2012).

### Total protease production

The proteolytic activity of all *P. aeruginosa* strains (treated and untreated) was estimated using the skimmed milk assay technique. The assay was performed by adding 1 mL of 1.25% (w/v) skimmed milk to 500 μL of culture supernatants and incubating for 1 h at 37 °C. The OD was measured at 600 nm and compared with that of the blank (skimmed milk). The reduction in the OD_600_ of the treated cells was compared with that of the untreated cells under the same conditions (Skindersoe et al. 2008).

### Real-time PCR

The effect of TMF on the expression of QS regulatory genes *lasI/R* and *rhlI/R* in *P. aeruginosa* PAO1 was measured using RT-PCR. The control untreated PAO1 and cultures treated with 1/2 MIC of TMF were grown until an OD 600 nm of 0.4–0.5. Cells were collected via centrifugation at 6000 × *g* for 15 min, and RNA was extracted using TRIZOL reagent (Oxoid, Basingstoke, Hants, UK), according to the manufacturer’s instructions. The SensiFAST™ cDNA Synthesis Kit (Bioline Reagents Ltd., London, UK) was used to synthesize complementary DNA. The Rotor-Gene Q thermocycler (Qiagen, Valencia, CA, USA) was used to perform RT-PCR. The primers described in Table 1 were used in the amplification reaction with TOPrealTM qPCR 2 × PreMIX (SYBR Green with low ROX) (Enzynomics; Daejeon, Korea). The expression of each gene was normalized to that of the *rpoD* housekeeping gene, and the relative expression was determined using the formula 2^−ΔΔCT^ (Livak and Schmittgen 2001). The expression of genes in PAO1 cultures treated with TMF was compared with that in control cultures without treatment.

**Table 1:** MICs and sub-MICs of TMF compound purified from *T. stellata*

Gene type	Gene name	Type of primer	Primer Sequence	Melting temp.	Amplicon size (bp)
Reference gene.	*rpoD*	Fw Rev	5`–CGAACTGCTTGCCGACTT–3` 5`–GCGAGAGCCTCAAGGATAC–3`	56°C	131
	*lasI*	Fw Rev	5`–CGCACATCTGGGAACTCA–3` 5`–CGGCACGGATCATCATCT–3`	56°C	176
QS genes.	*lasR*	Fw Rev	5`–CTGTGGATGCTCAAGGACTAC–3` 5`–AACTGGTCTTGCCGATGG–3	55°C	133
	*rhlI*	Fw Rev	5`–GTAGCGGGTTTGCGGATG–3` 5`–CGGCATCAGGTCTTCATCG–3`	58°C	101
	*rhlR*	Fw Rev	5`–GTAGCGGGTTTGCGGATG–3` 5`–CGGCATCAGGTCTTCATCG–3`	58°C	160

### Molecular docking

TMF was docked into the active site of *P. aeruginosa* LasR ligand-binding domain (Protein Data Bank ID: 2UV0) to evaluate the binding mechanism with LasR; this structure used 3-oxo-C_12_-HSL as an AI (Bottomley et al. 2007). All protein-bound water ligands were eliminated. ChemBioDraw was used to generate all components, which were then imported into the ChemBioOffice ultra v.14 programs. MM2, Jop type was used to minimize the energy, and docking was conducted using Molsoft (Abagyan et al. 1994).

### Data analysis and statistics

Experiments were performed in triplicate, and Excel was used to determine the means, standard deviations, and standard errors. Statistical analysis was calculated using Welch’s t-test. A significant difference between treated and untreated cultures was considered when the probability value was ***p ≤ 0.001, **p ≤ 0.01, or *p ≤ 0.05.

## Results

### Screening for QSI activity of *T. stellata*

The *T. stellata* ethanolic extract showed a potent QSI activity against *C. violaceum* ATCC 12472 after 24 h at 28 °C as shown by the inhibition of violacein pigment production to a diameter of 20 mm.

### Fractionation and structure elucidation of isolated compounds *in T. stellata*

The phytochemical analysis of *T. stellata* extracts in methylene chloride, ethyl acetate, and butanol resulted in isolation and identification of nine known compounds. Their structures were determined by analyzing 1D and 2D NMR spectra and mass spectrometry and by comparing their spectroscopic data with that published data (Ngoc et al., 2012; Shams Eldin et al., 2018).

The structures of isolated compounds (Fig. 1) were identified as kaempferitrin (1) (Figs. S1–S4) (Pereira et al. 2011), soyasaponin I (2) (Figs. S5–S11), β-sitosterol-3-*O*-glucoside (3) (Khan and Hossain 2015), dihydromelilotoside (4) (Figs. S12–S17), astrasikokioside I (5) (Figs. S18–S23) (Yahara et al. 2000), methyl dihydromelilotoside (6) (Figs. S24–S28), (3R, 4S)-4, 2′, 4′-trihydroxy-7-methoxy-4′-*O*-β-d-glucopyranosylisoflavan (7) (Figs. S29–S35), (3S, 4R)-4, 2′, 4′-trihydroxy-7-methoxyisoflavan (Figs. S36–S43), and (+)-d-pinitol (9) (Figs. S4–S47) (Ganbaatar et al. 2016). Interestingly, compounds 5, 6, and 9 have not been previously described from *T. stellate.*

**Fig. 1:**
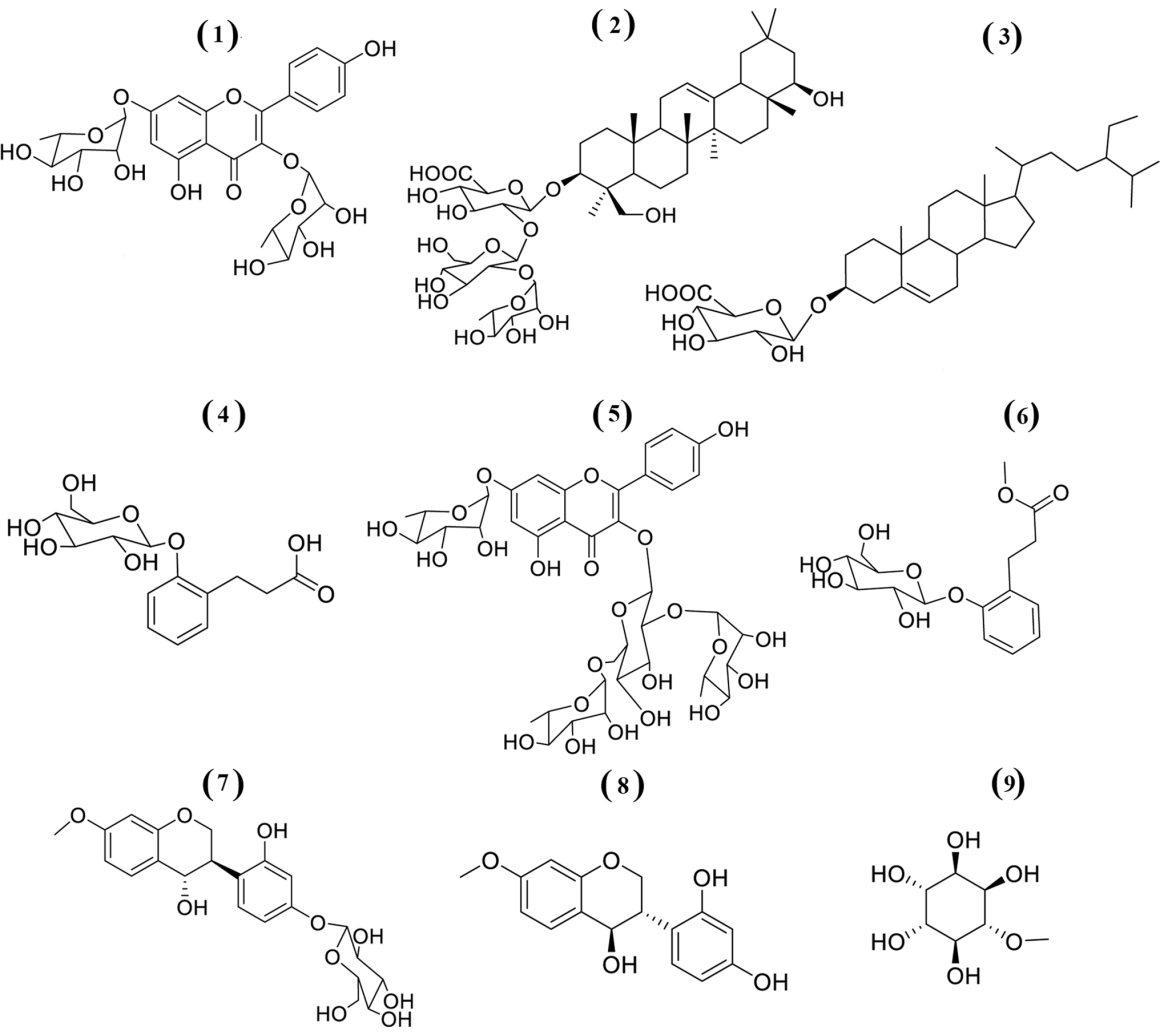
Structures of nine isolated compounds from *T. stellata*, kaempferitrin (**1**), soyasaponin I (**2**), β-sitosterol-3-O-glucoside (**3**), dihydromelilotoside (**4**), astrasikokioside I (**5**), methyl dihydromelilotoside (**6**), (3R,4S)-4,2`,4`-trihydroxy-7-methoxy-4`-O-β-D-glucopyranosylisoflavan (**7**), (3S,4R)-4,2`,4`-trihydroxy-7-methoxyisoflavan (**8**, TMF), and (+)-D-pinitol (**9**)

### *Chromobacterium violaceum* ATCC 12472 bioassay of QSI activity of compounds isolated from *T. stellata*

The QSI activity of nine compounds isolated from *T. stellata* was screened using *C. violaceum* ATCC 12472. Compound 8, TMF, exhibited potent anti-QS activity with an inhibition in the diameter of the violet pigmentation by 15 mm. Compounds 1, 3, 5, 6, and 9 showed weak QSI effect with inhibition of the violet pigment diameter by 2, 5, 5, 3, and 2 mm, respectively. Therefore, fraction 8 was chosen for further investigations of antipathogenic potential against *P. aeruginosa* standard and clinical strains (Table 2).

**Table 2:** Inhibition of violet pigment of the reporter strain *Chromobacterium violaceum* by the purified compounds from *T. stellata*

No	Compound	Diameter of violacein inhibition (mm)
1	Kaempferitrin	2
2	Soyasaponin	–
3	β-Sitosterol-3-*O*-glucoside	5
4	Dihydromelilotoside	–
5	Astrasikokioside I	5
6	Methyl dihydromelilotoside	3
7	(3R, 4S)-4, 2′, 4′-trihydroxy-7-methoxy-4′-*O*-β-d-glucopyranosylisoflavan	–
8	(3S, 4R)-4, 2′, 4′-trihydroxy-7-methoxyisoflavan (TMF)	15
9	(+)-d-pinitol	2

### Antibacterial screening

The MIC of TMF against *P. aeruginosa* strains Ps.A11, Ps.A12, Ps.A13, Ps.A16, PA14, PAO1, and PAO-JP2 was 512 μg/mL. Sub-MICs at 1/2 and 1/4 strength were calculated as 256 and 128 μg/mL, respectively (Table 3).

**Table 3:** MICs and sub-MICs of TMF compound purified from *T. stellata*

	MIC (µg/mL)	1/2 MIC (µg/mL)	1/4 MIC (µg/mL)
*P. aeruginosa* Ps.A11	512	256	128
*P. aeruginosa* Ps.A12	512	256	128
*P. aeruginosa* Ps.A13	512	256	128
*P. aeruginosa* Ps.A16	512	256	128
*P. aeruginosa* PAO1	512	256	128
*P. aeruginosa* PA14	512	256	128
*P. aeruginosa* PAO-JP2	512	256	128

### Effect of the sub-MIC of TMF on *P. aeruginosa* viability

The antimicrobial effect of TMF on *P. aeruginosa* viability was determined before and after supplying growing cells with 1/2 MIC (256 μg/mL) for 24 h at 37 °C. The bacterial count from untreated cultures was 112, 94, 126, 113, 98, 133, and 103 × 10^7^ CFU/mL for Ps.A11, Ps.A12, Ps.A13, Ps.A16, PA14, PAO1, and PAO-JP2, respectively. The count from the treated cultures was 108, 95, 121, 107, 96, 129, and 100 × 10^8^ CFU/mL for Ps.A11, Ps.A12, Ps.A13, Ps.A16, PA14, PAO1, and PAO-JP2, respectively. There was no significant difference between the bacterial count between untreated and treated cultures. Furthermore, no effect on bacterial growth was observed in cultures treated with 1/2 MIC of TMF in comparison with that in untreated cultures (Fig. S48).

### Antivirulence effect of TMF on *P. aeruginosa* strains

Treatment with both the sub-MICs of TMF compound (1/2 and 1/4) significantly inhibited formation of biofilm and pyocyanin and activity of hemolysin and protease in Ps.A11, Ps.A12, Ps.A13, Ps.A16, PA14, and PAO1 as compared with that in untreated cultures. PAO-JP2 had the lowest activity among the tested strains for all virulence factors.

### Inhibition of biofilm formation

**Fig. 2:**
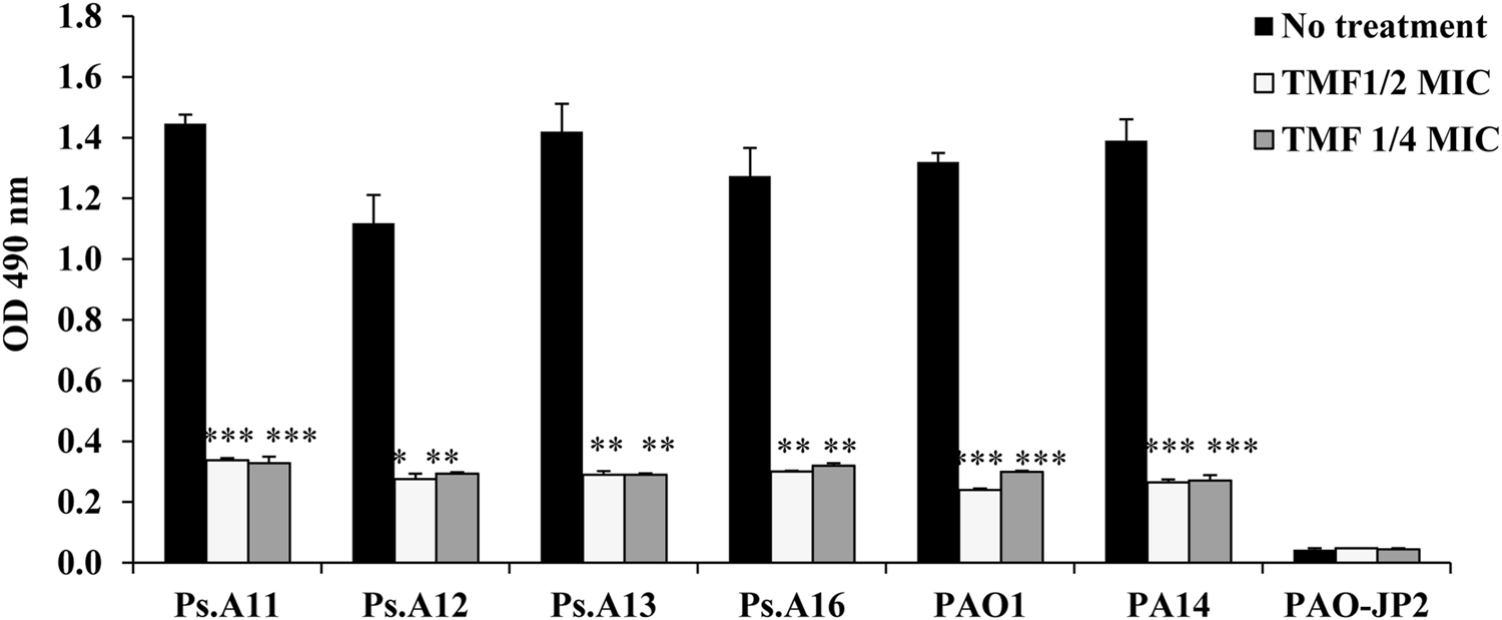
Effect of 1/4 and 1/2 sub-MICs of (3S,4R)-4,2`,4`-trihydroxy-7-methoxyisoflavan (TMF) isolated from *T. stellata* on biofilm formation by *P. aeruginosa* strains; Ps.A11, Ps.A12, Ps.A13, Ps.A16, PA14, PAO1, and PAO-JP2 compared to the untreated cultures. Error bars show standard deviation of three replicates, ***p ≤0.001, **p ≤ 0.01

A sub-MIC of TMF (256 μg/mL) significantly reduced biofilm formation in Ps.A11, Ps.A12, Ps.A13, Ps.A16, PA14, and PAO1 by 76.5%, 75.3%, 79.6%, 76.3%, 81%, and 81.8%, respectively. Furthermore, 1/4 MIC significantly decreased biofilm by 77.2%, 73.7%, 79.6%, 74.9%, 80%, and 77.3% in Ps.A11, Ps.A12, Ps.A13, Ps.A16, PA14, and PAO1, respectively (Fig. 2; Table S1).

### Pyocyanin inhibition

**Fig. 3:**
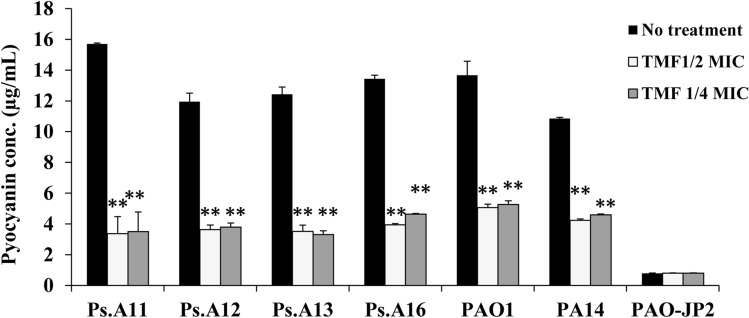
Effect of 1/4 and 1/2 sub-MICs of (3S,4R)-4,2`,4`-trihydroxy-7-methoxyisoflavan (TMF) on pyocyanin production by *P. aeruginosa* strains; Ps.A11, Ps.A12, Ps.A13, Ps.A16, PA14, PAO1, and PAO-JP2 compared to the untreated cultures. Error bars show standard deviation of three replicates, ***p ≤ 0.001, **p ≤ 0.01

Low concentrations of TMF at sub-MICs (1/2 and 1/4) significantly inhibited pyocyanin production in all tested *P. aeruginosa* strains. TMF at 1/2 MIC decreased pyocyanin by 78.6%, 69.6%, 71.7%, 70.7%, 61%, and 63.2%, respectively, in all tested strains (Ps.A11, Ps.A12, Ps.A13, Ps.A16, PA14, and PAO1) (*p* ≤ 0.001), also the 1/4 MIC of TMF significantly decreased pyocyanin by 77.7%, 68.3%, 73.4%, 65.5%, 57.7%, and 61.8%, respectively, in these strains as well (*p* ≤ 0.001, Fig. 3; Table S2).

### Decrease in hemolysin activity

**Fig. 4:**
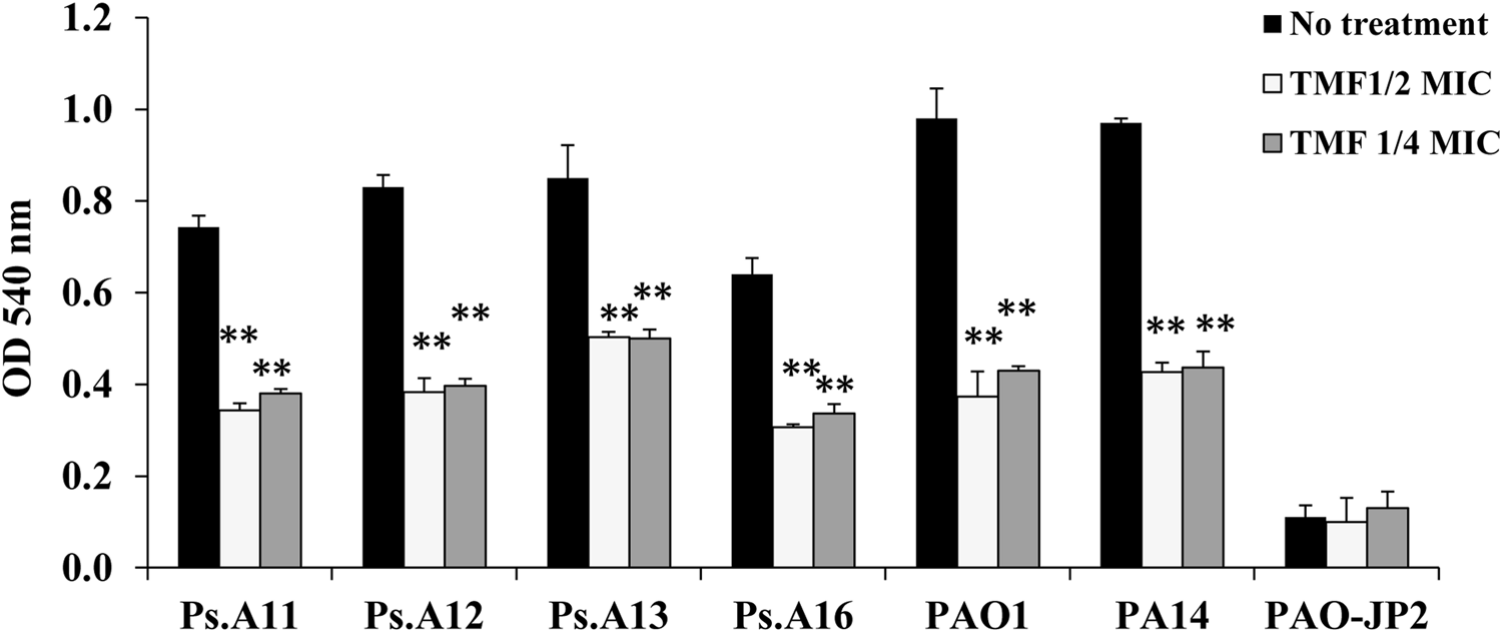
Effect of 1/4 and 1/2 sub-MICs of (3S,4R)-4,2`,4`-trihydroxy-7-methoxyisoflavan (TMF) isolated from *T. stellata* on hemolysin activity by *P. aeruginosa* strains; Ps.A11, Ps.A12, Ps.A13, Ps.A16, PA14, PAO1, and PAO-JP2 compared to the untreated cultures. Error bars show standard deviation of three replicates, **p ≤ 0.01, *p ≤ 0.05

TMF at sub-MICs significantly inhibited hemolysin activity (*p* ≤ 0.001) in isolates Ps.A11, Ps.A12, Ps.A13, Ps.A16, PAO1, and PA14. The 1/2 MIC decreased hemolysin production by Ps.A11, Ps.A12, Ps.A13, Ps.A16, PAO1, and PA14 by 53.8%, 53.8%, 40.8%, 52.1%, 61.8%, and 56%, respectively, whereas the 1/4 MIC (128 μg/mL) lowered hemolysin activity by 48.9%, 52.2%, 41.2%, 47.4%, 56.1%, and 55%, respectively (Fig. 4; Table S3).

### Decrease in protease activity

**Fig. 5:**
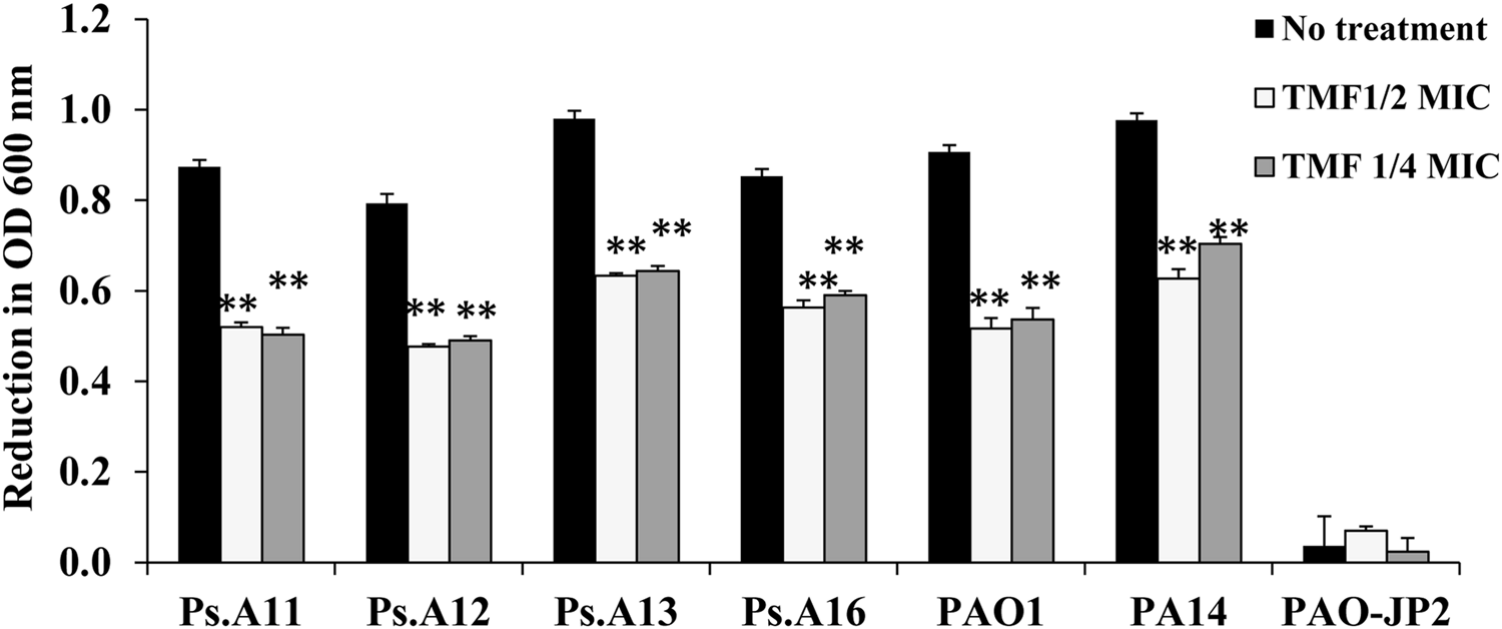
Effect of 1/4 and 1/2 sub-MICs of (3S,4R)-4,2`,4`-trihydroxy-7-methoxyisoflavan (TMF) on protease production by *P. aeruginosa* strains; Ps.A11, Ps.A12, Ps.A13, Ps.A16, PA14, PAO1, and PAO-JP2 compared to the untreated cultures. Error bars show standard deviation of three replicates, ***p ≤ 0.001, **p ≤ 0.01

Protease production was significantly reduced at 1/2 MIC of TMF by 40.2%, 39.7%, 35.4%, 33.7%, 36.1%, and 43.2% in Ps.A11, Ps.A12, Ps.A13, Ps.A16, PA14, and PAO1, respectively, compared with that in untreated cultures. The 1/4 MIC significantly reduced protease activity by 42.1%, 38%, 34.4%, 30.6%, 28%, and 41% in Ps.A11, Ps.A12, Ps.A13, Ps.A16, PA14, and PAO1, respectively (Fig. 5; Table S4).

### Suppression of the QS regulatory genes

**Fig. 6:**
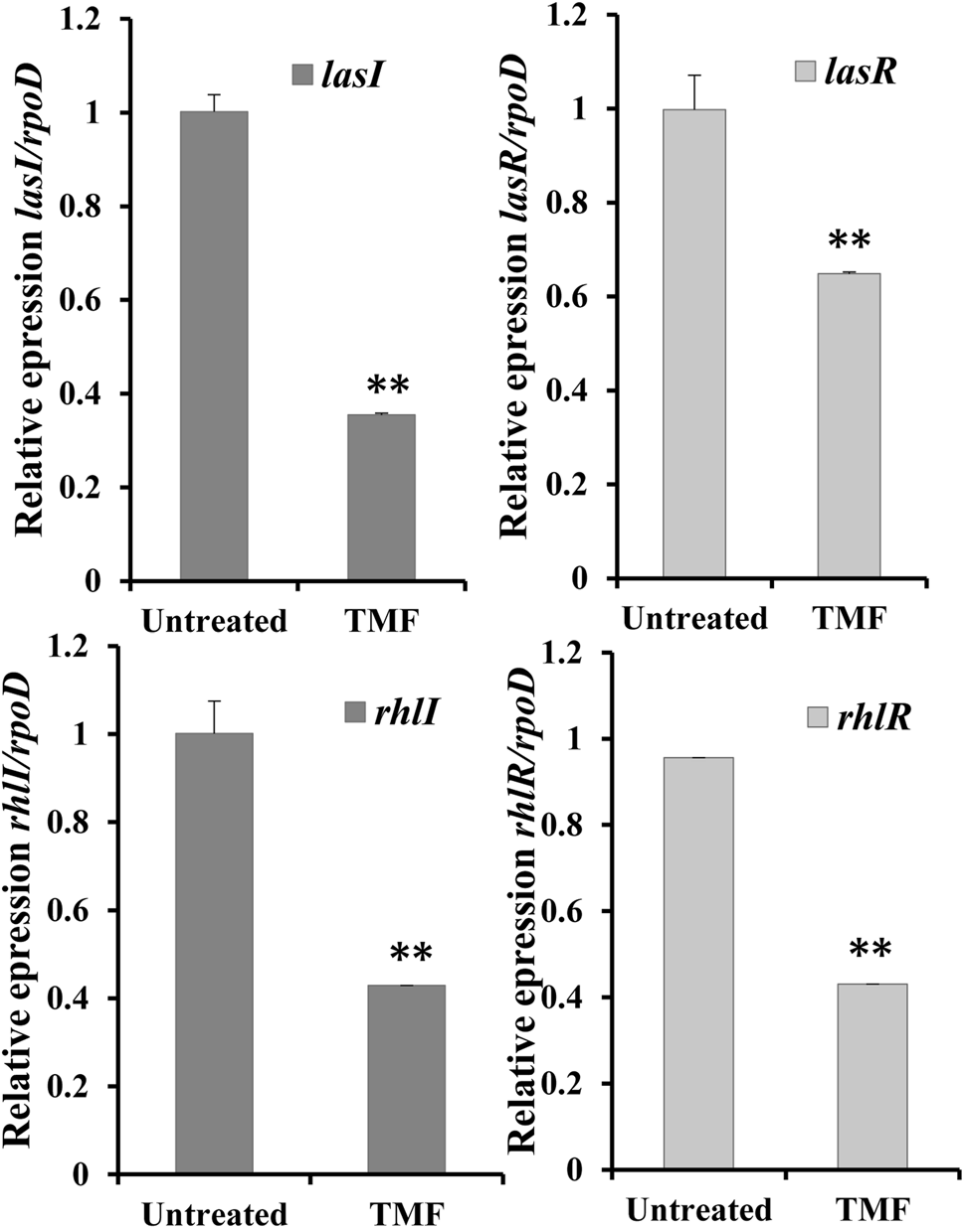
Relative expression of *lasI, lasR, rhlI* and *rhlR* in *P. aeruginosa* PAO1 treated with 1/2 MIC of (3S,4R)-4,2`,4`- trihydroxy-7-methoxyisoflavan (TMF) compared to untreated cultures. Error bars show standard deviation of three replicates, ***p ≤ 0.001, **p ≤ 0.01

We investigated the effect of TMF at 1/2 MIC on gene expression involved in the QS circuit of *P. aeruginosa.* RT-PCR was used to assess the relative expression levels of *lasI*, *lasR*, *rhlI*, and *rhlR* genes. TMF significantly (*p* < 0.01) lowered the mRNA level of *lasI* and *lasR* by 64.5% and 34%, respectively, in the standard strain PAO1 compared with that in the untreated cultures (Fig. 6).

Moreover, the relative expression of *rhlI* and *rhlR* genes in *P. aeruginosa* PAO1 treated with 1/2 MIC of TMF were significantly reduced compared with that in the untreated cells. The relative expression of *rhlI* and *rhlR* mRNA were significantly reduced by 57% and 56.9%, respectively (*p* < 0.01) compared with that in the untreated PAO1 (Fig. 6).

### Binding affinity analysis for LasR and ligands by molecular docking

The LasR ligand, 3-oxo-C_12_-HSL, was redocked with LasR to assess the docking technique. This showed that the ICM score was − 107.47 and that three H-bonds were formed with Asp73, Trp60, and Ser129. Thus, these three amino acids are crucial for binding with the LasR active site. Docking of TMF with the LasR had an ICM score of − 74.35 (Table 4).

**Table 4:** Scores based on ICM, the number of H-bonds, and the residues of amino acids involved in the LasR binding site with TMF and 3-oxo-C_12_-HSL

Compound	ICM score with LasR	No. of H-bonds	Residues of amino acids
3-Oxo-C_12_-HSL	− 107.47	3	Trp60, Asp73, Ser129
TMF	− 74.35	6	Thr 75, Ser 129, Asp 73, Leu 110

TMF formed six H-bonds, one each with both Leu110 and Ser129 and two each with Thr75 and Asp73 (Table 5; Fig.

**Table 5:** Molecular docking results of TMF with interacting amino acids with LasR of *P. aeruginosa*

Amino acids' residues	Atom of amino acids	Atom of compound	Length (Å)
Thr75	Hg_1_	O_3_	1.28
Ser129	Hg	O_1_	2.7
Asp73	Od_1_	H_12_	1.3
Asp73	Od_2_	H_12_	2.7
Thr75	Og_1_	H_12_	2.74
Leu110	O	H_13_	2.3

7).

**Fig. 7:**
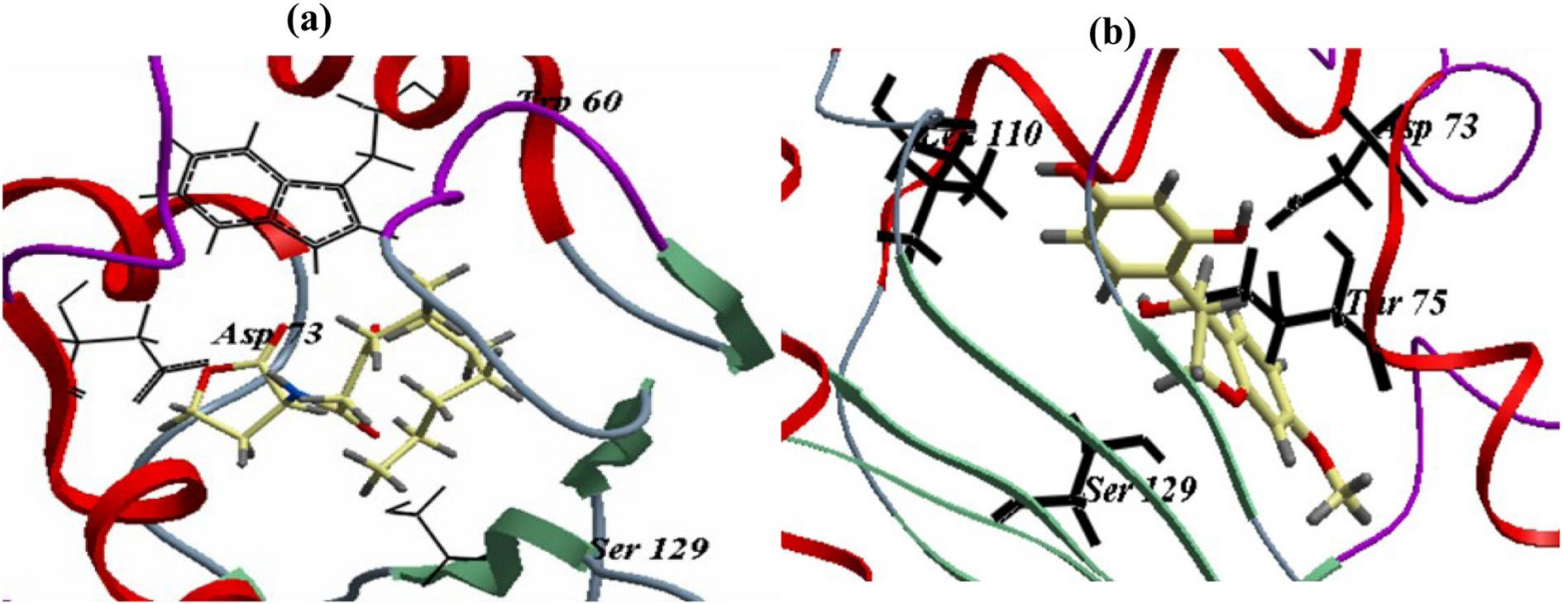
Binding mode of 3-oxo-C12-HSL (**a**) and (3S,4R)-4,2`,4`-trihydroxy-7-methoxyisoflavan (TMF) compound (**b**) docked into the active site of lasR

## Discussion

QS is the key regulatory mechanism that manages bacterial pathogenesis via gene expression and virulence behavior. Treatment of *P. aeruginosa* infection is challenging due to the ability of this bacterium to form a biofilm that is resistant to conventional antibiotics (Pérez-Pérez et al. 2017). The antimicrobial resistance of *Pseudomonas* is also associated with the production of degradative enzymes that can inactivate antimicrobials or the production of debasing chemicals and efflux pumps from chromosomally encoded antibiotic-resistant genes (Du et al. 2013). Accordingly, developing new strategies to manage *Pseudomonas* infection is essential. One approach is to focus on inhibiting QS crosstalk as this could critically inhibit virulent effects. Violacein production in *C. violaceum* ATCC 12472 is controlled by *N*-acyl-l-homoserine lactones mainly *N*-(3-hydroxydecanoyl)-l-homoserine lactone (AHL). In addition, other signaling molecules, such as 3-oxo-C_10_-HSL and 3-oxo-C_12_-HSL, can enhance violacein production in this bacterium (Morohoshi et al. 2008, 2010; Mion et al. 2021). In previous research, *C. violaceum* ATCC 12472 has been used to assay violacein inhibition by glyceryl trinitrate (Abbas and Shaldam 2016), silver nanoparticles (Shah et al. 2019), and *Spirulina platensis* (Lewis Oscar et al. 2018).

*Trigonella stellata* belongs to the genus *Trigonella*, which can be found in dry places across the Mediterranean (Turki et al. 2013). In China, Egypt, and India, *Trigonella* has a long history of traditional use as a medicine (Saxena and Albert 2005). *Trigonella* seeds have been traditionally used as a laxative and antipyretic (Yoshikawa et al. 1998) and have been used to treat gastrointestinal diseases, hyperlipidemia, inflammation, and diabetes for centuries (Shah et al. 2006). *Trigonella* can induce diverse pharmacological effects that are primarily due to the presence of diosgenin, flavonoids, trigonelline, coumarin, furostanol, and flavonol glycosides (Sheweita et al. 2020). In this study, we validated *T. stellata* for anti-QS activities. Extracts from *T. stellata* were highly potent in inhibiting violet pigment formation, which indicated possible interference with AHL-based signaling (Table 2). The bioassay of QSI activity of the nine purified compounds from *T. stellata* revealed that the highest QSI was mediated by the TMF compound. Therefore, TMF was assessed for its ability to inhibit QS-related virulence factors.

*Pseudomonas aeruginosa* causes nosocomial infections that are typically associated with the formation of a biofilm that is resistant to most antimicrobials (Pérez-Pérez et al. 2017). Herein, TMF could significantly reduce biofilm formation with all pseudomonad strains tested (Fig. 2), which would consequently make the bacteria more vulnerable to the response of the immune system (Rasmussen et al. 2005). TMF compound is considered a flavonoid derivative. The antibiofilm effects of flavonoids, such as apigenin, luteolin, quercetin, fisetin, and chrysin, on *Staphylococcus aureus* have also been reported (Cho et al. 2015).

The RhlI/R and PQS signaling systems of *P. aeruginosa* also regulate the production of a distinct green pigment called pyocyanin (Gupta et al. 2011). The absence of this green pigment indicates lower regulatory control of pyocyanin production (Fig. 3). Treatment of all tested *P. aeruginosa* strains with TMF at low concentrations (128–256 μg/mL) resulted in a significant reduction in pyocyanin levels, indicating inhibition of the C_4_-HSL and pqsR signaling molecules of the RhlI/R and PQS signaling systems, respectively.

*Pseudomonas aeruginosa* produces extracellular enzymes such as protease and hemolysin that allow the bacteria to spread inside host tissues and to resist host immunity (Gupta et al. 2011). We found that TMF significantly suppressed the activity of these factors, which are regulated by the LasI/LasR system, to varying degrees (Figs. 4, 5). Similarly, the flavonoid fraction of *Psidium guajava* leaves extract was shown to decrease *P. aeruginosa* PAO1 virulence factors (Vasavi et al. 2014). Flavonoids, such as taxifolin, naringenin, catechin, and flavanes-3-ol, have been shown to have a significant effect on the Las system in *P. aeruginosa* PAO1 (Vandeputte et al., 2011; Rasamiravaka et al., 2013; Bouyahya et al., 2017), and flavonoids from *Centella asiatica* plant have also been shown to reduce the proteolytic activities of this strain (Vasavi et al., 2016).

In *P. aeruginosa*, LasI/R act as transcriptional activators that manipulate the expression of QS cascades while RhlI/R and RqsR regulate the expression of the associated virulence factors [80, 81]. In this study, the relative mRNA expression of *lasI/lasR* and *rhlI/rhlR* was significantly reduced by treatment with TMF at sub-MIC (Fig. 6). The low levels of these factors were associated with QS interruption, confirming the influence of QSI on production of virulence factors of the tested strains.

The molecular docking was performed to evaluate the structural basis of QSI activity of TMF compound (Abagyan et al. 1994). In *P. aeruginosa*, the Las system is the main QS regulator and is activated by the 3-oxo-C_12_-HSL signaling molecule, which controls the QS network (Pesci et al. 1997; Venturi 2006). We retrieved the structure of LasR receptor protein (PDB ID 2UV0) (https://www.rcsb.org/structure/2UV0). The scoring functions and hydrogen bonds produced by the LasR active site with the surrounding amino acids could be used to determine the binding mode, affinity, and orientation of TMF (Fig. 7). Analysis using Pdbsum revealed that the important amino acids that bind with LasR active site are Cys79, Tyr56, Ser129, Ala50, Trp60, Thr75, Tyr93, Leu110, Asp73, Trp88, Ala105, Tyr64, and Gly126 (http://www.ebi.ac.uk/thornton-srv/databases/cgi-bin/pdbsum /GetPage.pl). In 2017, pyridoxal lactohydrazone was discovered to reduce QS-related virulence factors in *P. aeruginosa* and to establish hydrogen bonds with Thr75, Trp60, Ser129, and Arg61 in the active site of LasR (Heidari et al. 2017). Furthermore, fenaclon competitively binds to LasR via the formation of hydrogen bonds with Asp73, Ser129, and Tyr56 (Pattnaik et al. 2018). In this study, TMF formed six H-bonds, one each with Leu110 and Ser129 and two each with Thr75 and Asp73; these bonds would hinder the binding of the natural ligand 3-oxo-C_12_-HSL with its receptor and interrupt the signaling cascade (Table 5).

## Conclusion

This study highlights the importance of the medicinal plant *T. stellate* and its active metabolite TMF as a potent QSI of *P. aeruginosa* without any effect on bacterial viability. This inhibitory effect could be attributed to the effect of TMF at sub-inhibitory concentrations on the relative expression of QS genes. Reduction in the relative expression of *lasI/lasR* and *rhlI/rhlR* was associated with a significant inhibition of virulence factors (biofilm formation, pyocyanin production, and hemolysin and protease activity). Furthermore, these in vitro results were confirmed using a molecular docking study that calculated the binding affinity of TMF with LasR. Overall, our study shows that TMF can combat the pathogenesis and dissemination of *Pseudomonas* infection through QS interference*.* Further studies need to be performed to evaluate the in vivo antipathogenic effects of TMF.

## Supplementary Information

Below is the link to the electronic supplementary material.

Supplementary file1 (PDF 2195 kb)

## Data Availability

The original contributions presented in the study are included in the article/Supplementary Material. Further inquiries can be directed to the corresponding authors.
