# Supplementary material for: Methoxyisoflavan derivative from Trigonella stellata inhibited quorum sensing and virulence factors of Pseudomonas aeruginosa
Source: World J Microbiol Biotechnol. 2022 Jul 8;38(9):156. doi: 10.1007/s11274-022-03337-x (PMC9262770; doi:10.1007/s11274-022-03337-x)
Supplement: Supplementary file 1 — Supplementary file1 (PDF 2195 kb) [file 11274_2022_3337_MOESM1_ESM.pdf]

Methoxyisoflavan Derivative from *Trigonella stellata* Inhibited Quorum Sensing and Virulence factors of *Pseudomonas aeruginosa*

Nourhan G. Naga[1†], Ahmed A. Zaki [2,3†], Dalia E. El-Badan[1], Heba S. Rateb[4] Khaled M. Ghanem[1], Mona I. Shaaban[5]

[1]Botany and Microbiology Department, Faculty of Science, Alexandria University, Egypt.

[2]Pharmacognosy Department, Faculty of Pharmacy, Mansoura University, Mansoura 35516, Egypt.

[3]Pharmacognosy Department, Faculty of Pharmacy, Horus University-Egypt, New Damietta 34518, Egypt.

[4]Pharmaceutical and Medicinal Chemistry Department of, Pharmacy College, Misr University for Science and Technology, Cairo, Egypt.

[5]Microbiology and Immunology Department, Faculty of Pharmacy, Mansoura University, Egypt.

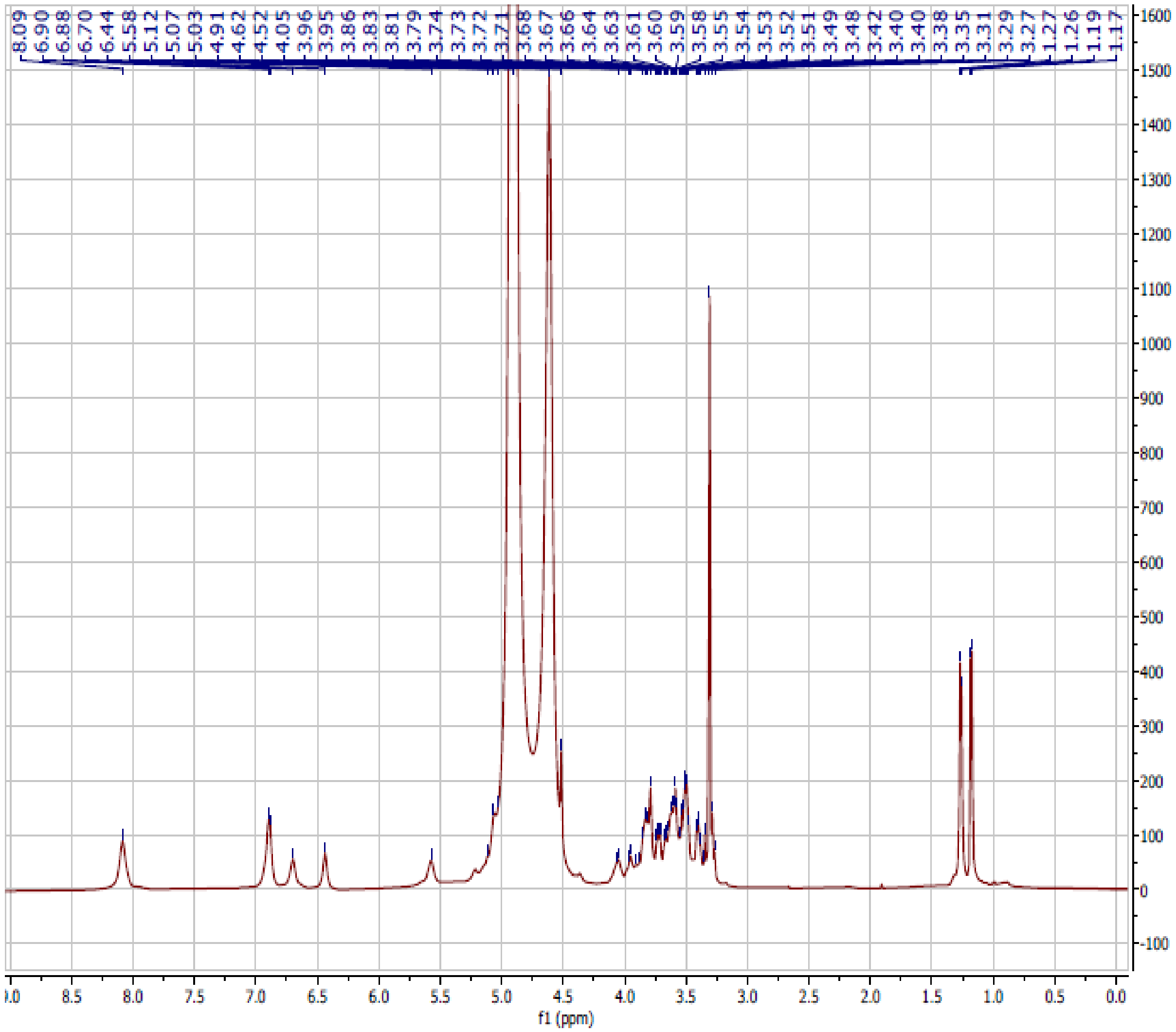

Supplementary figure S1: ^{1}H-NMR Spectrum of Compound **1** (400 MHz, CD3OD)

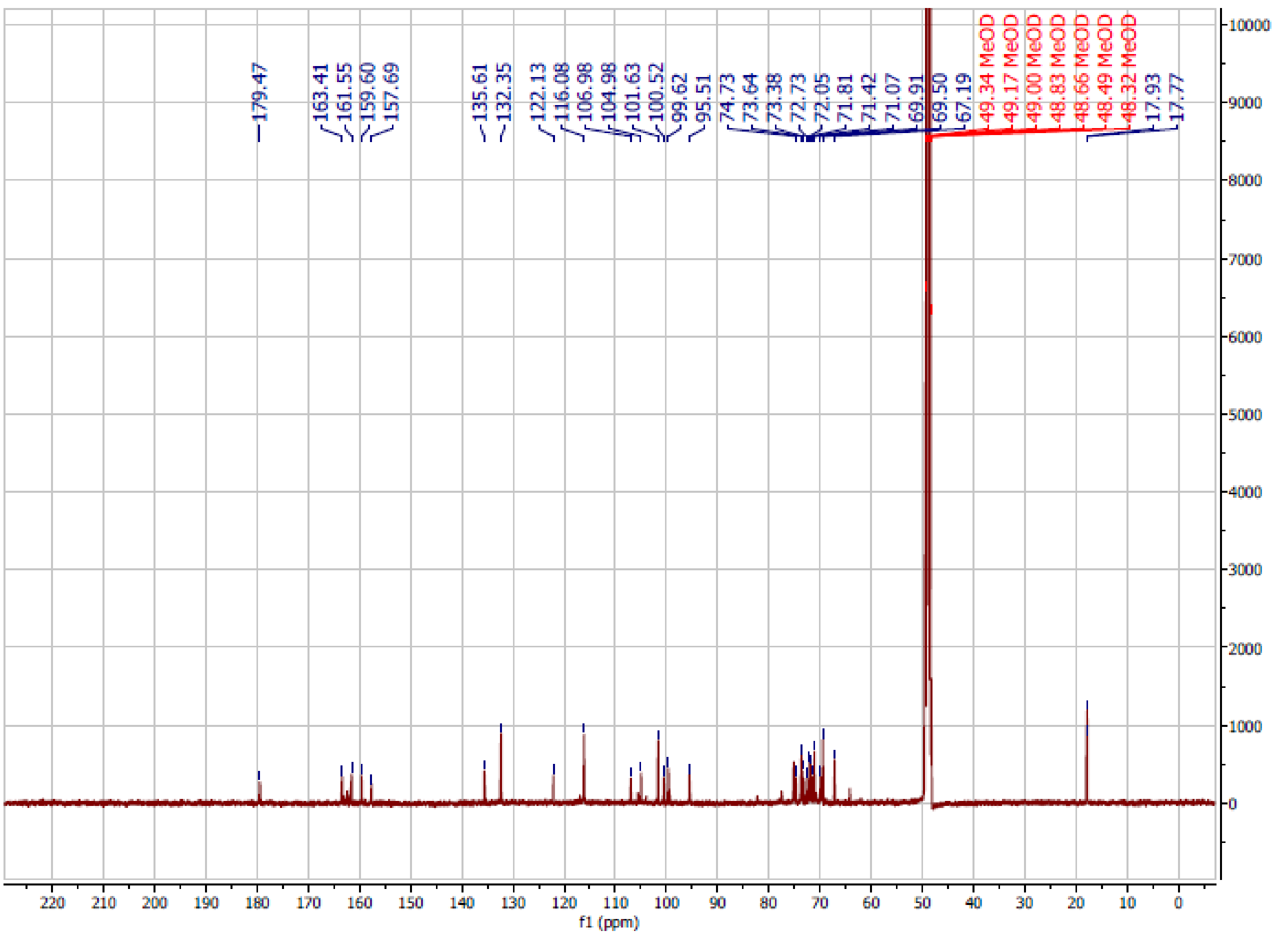

Supplementary figure S2:^{13}C-NMR Spectrum of Compound **1** (100 MHz, CD3OD)

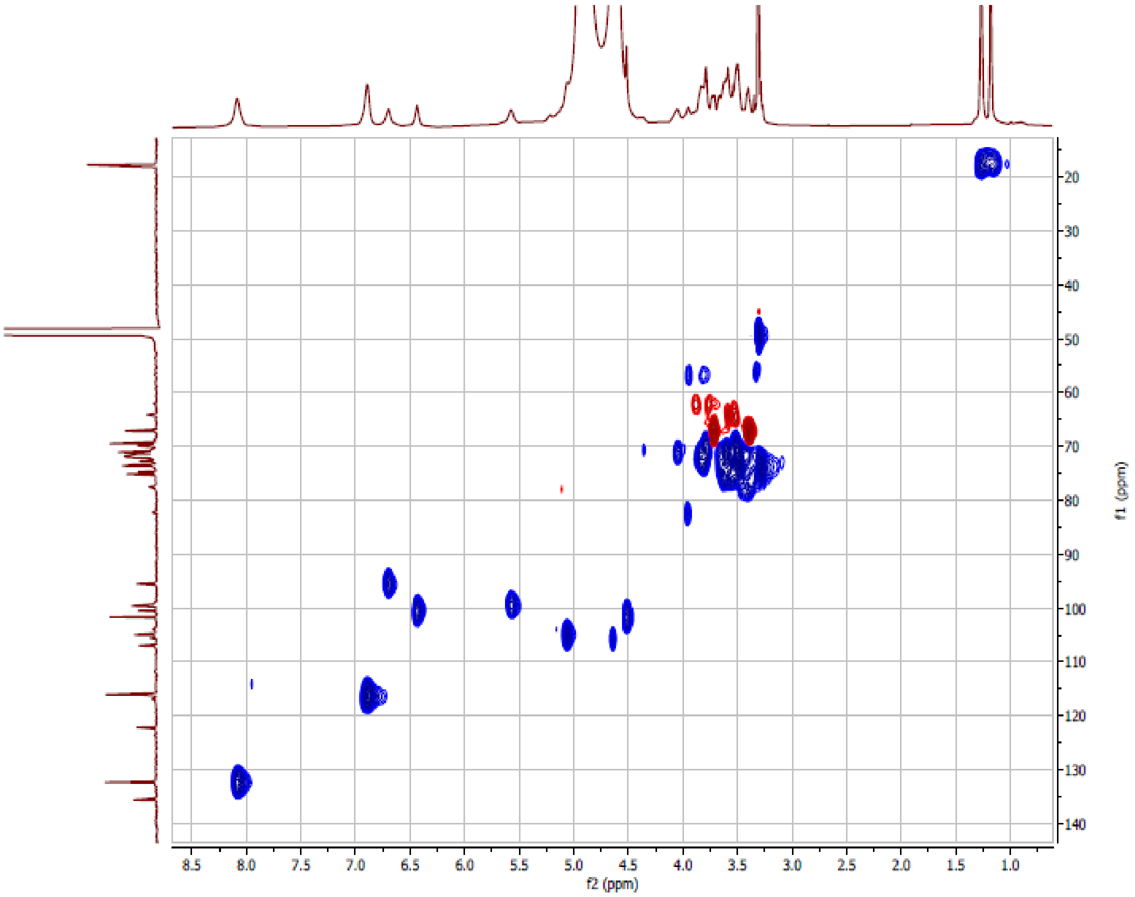

Supplementary figure S3: HSQC Spectrum of Compound **1** (400 MHz, CD3OD)

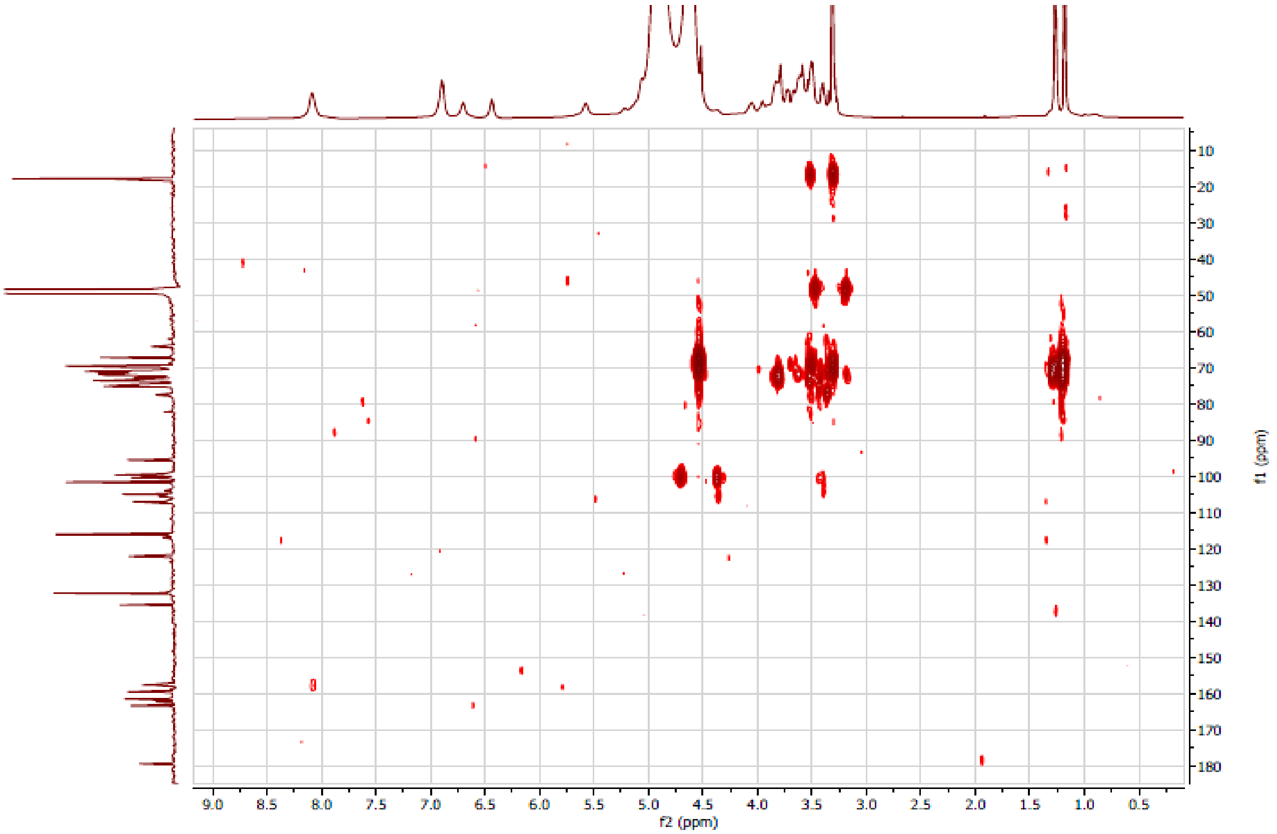

Supplementary figure S4: HMBC Spectrum of Compound **1** (400 MHz, CD3OD)

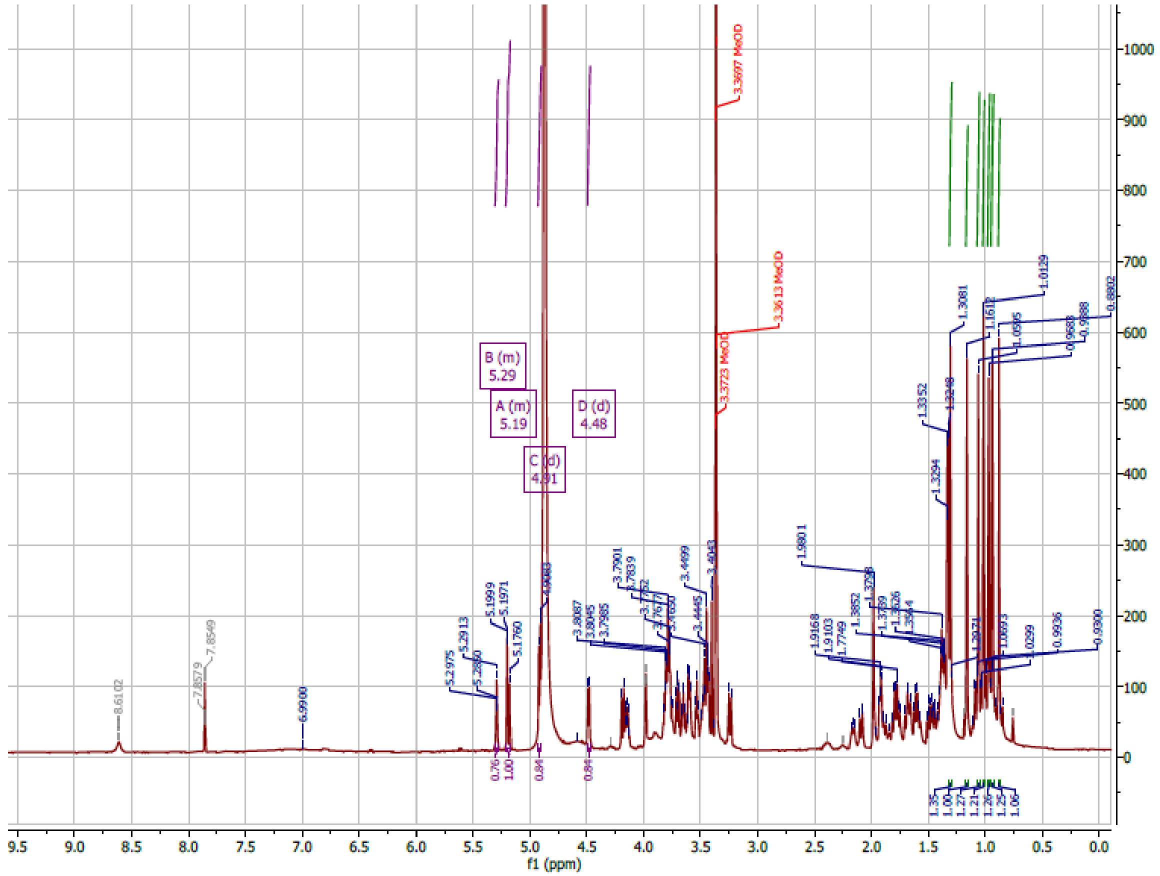

Supplementary figure S5: ^{1}H-NMR Spectrum of Compound **2** (400 MHz, CD3OD)

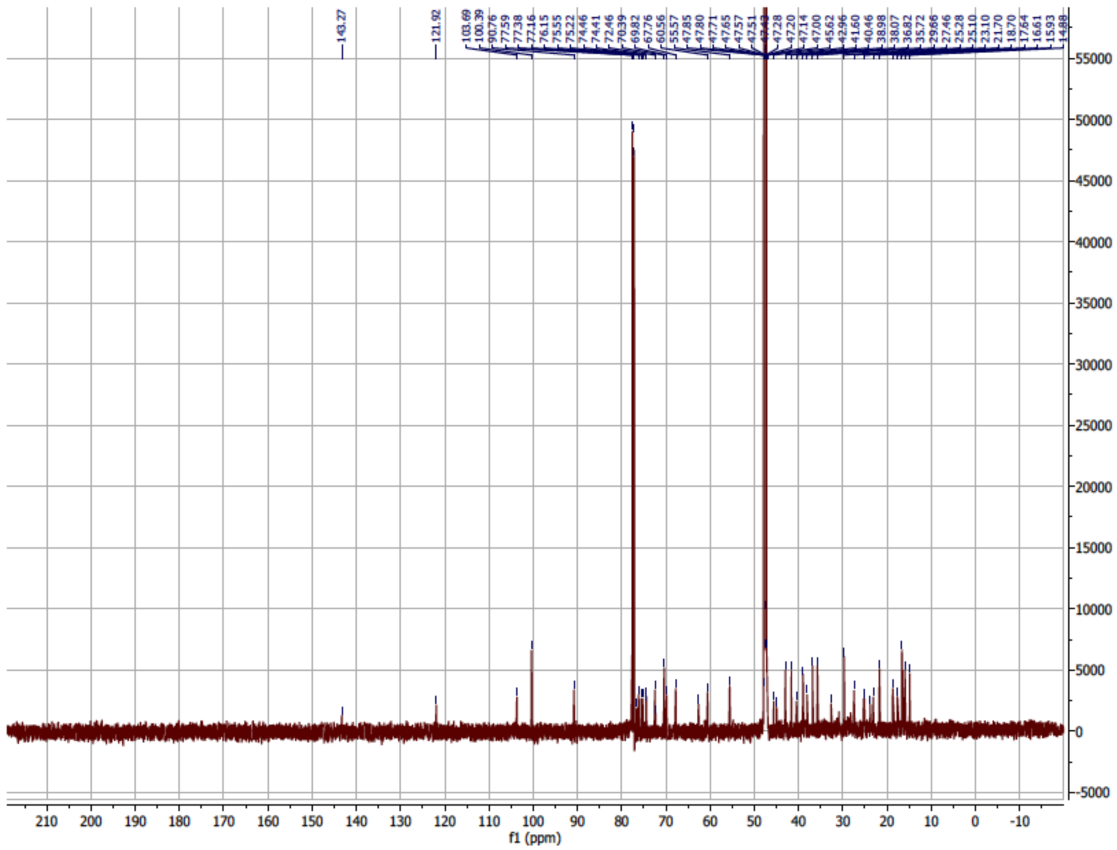

Supplementary figure S6: ^{13}C-NMR Spectrum of Compound **2** (100 MHz, CD3OD)

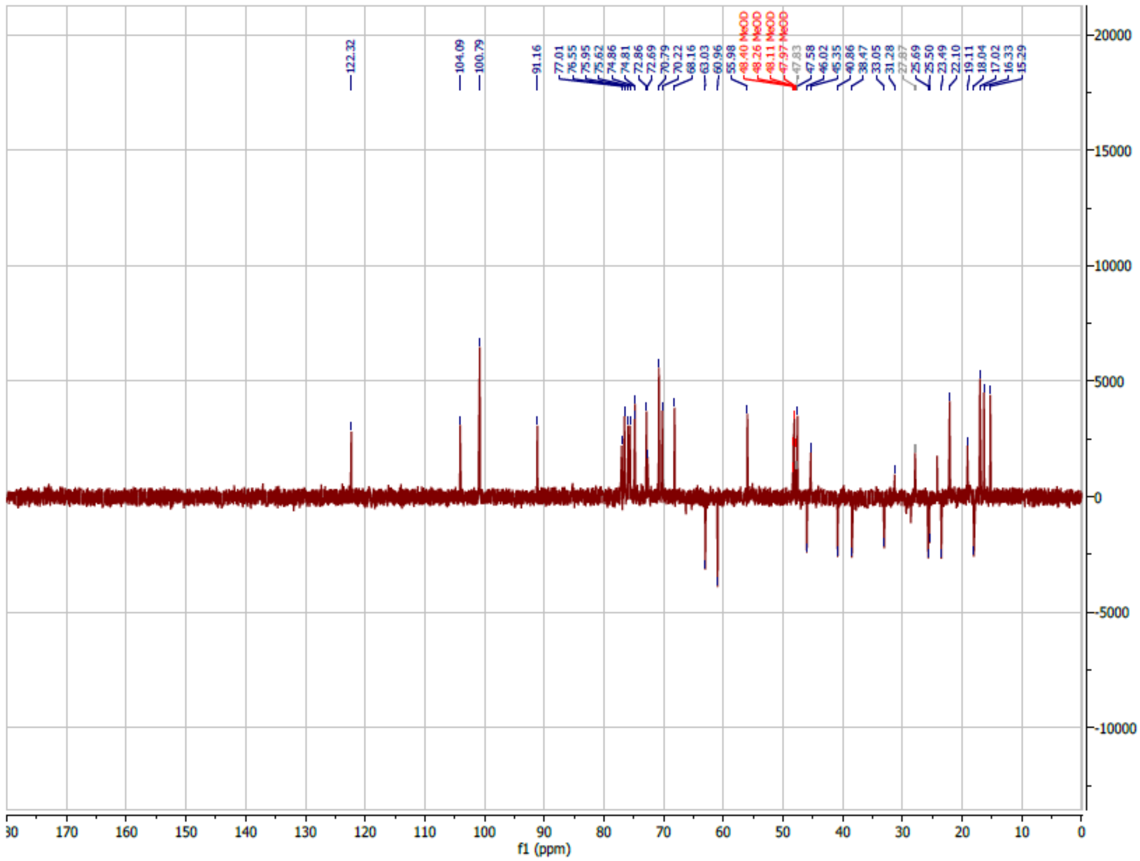

Supplementary figure S7: DEPT Spectrum of Compound **2** in CD3OD

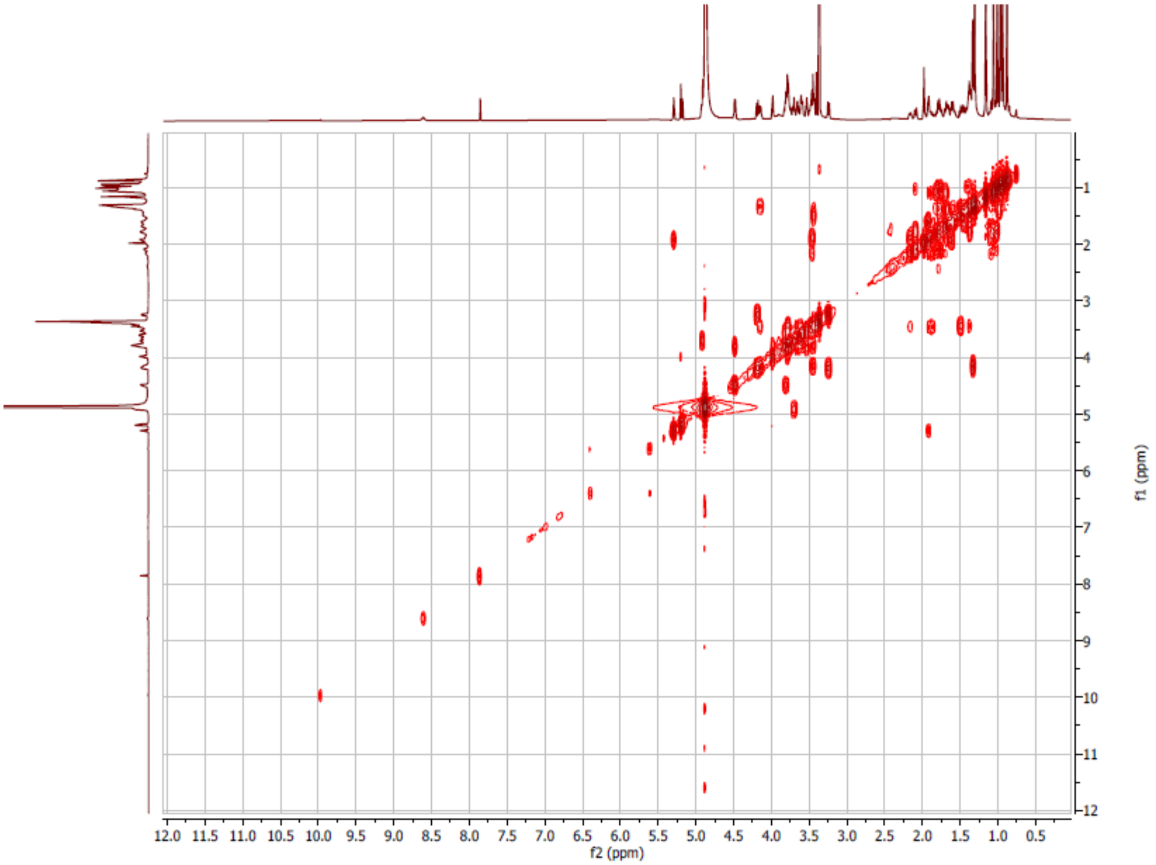

Supplementary figure S8: COSY Spectrum of Compound **2** (400 MHz, CD3OD)

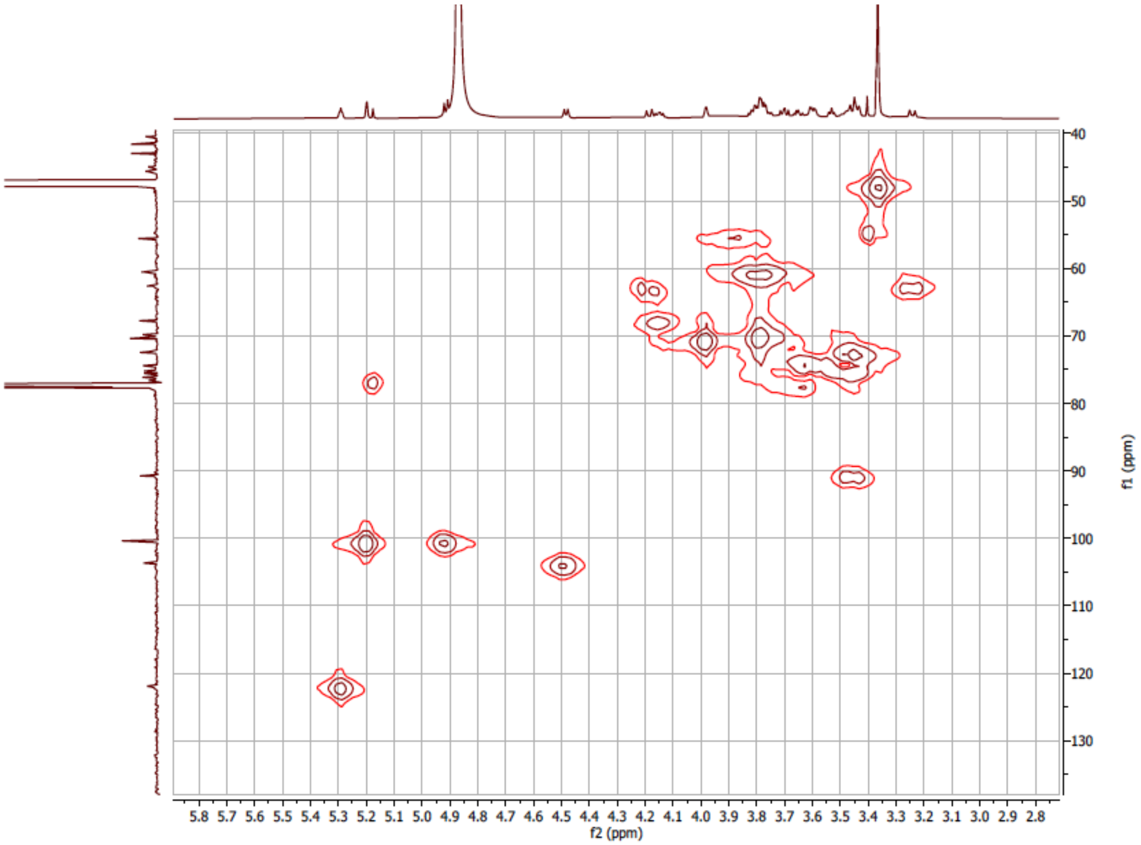

Supplementary figure S9: HMQC Spectrum of Compound **2** (400 MHz, CD3OD)

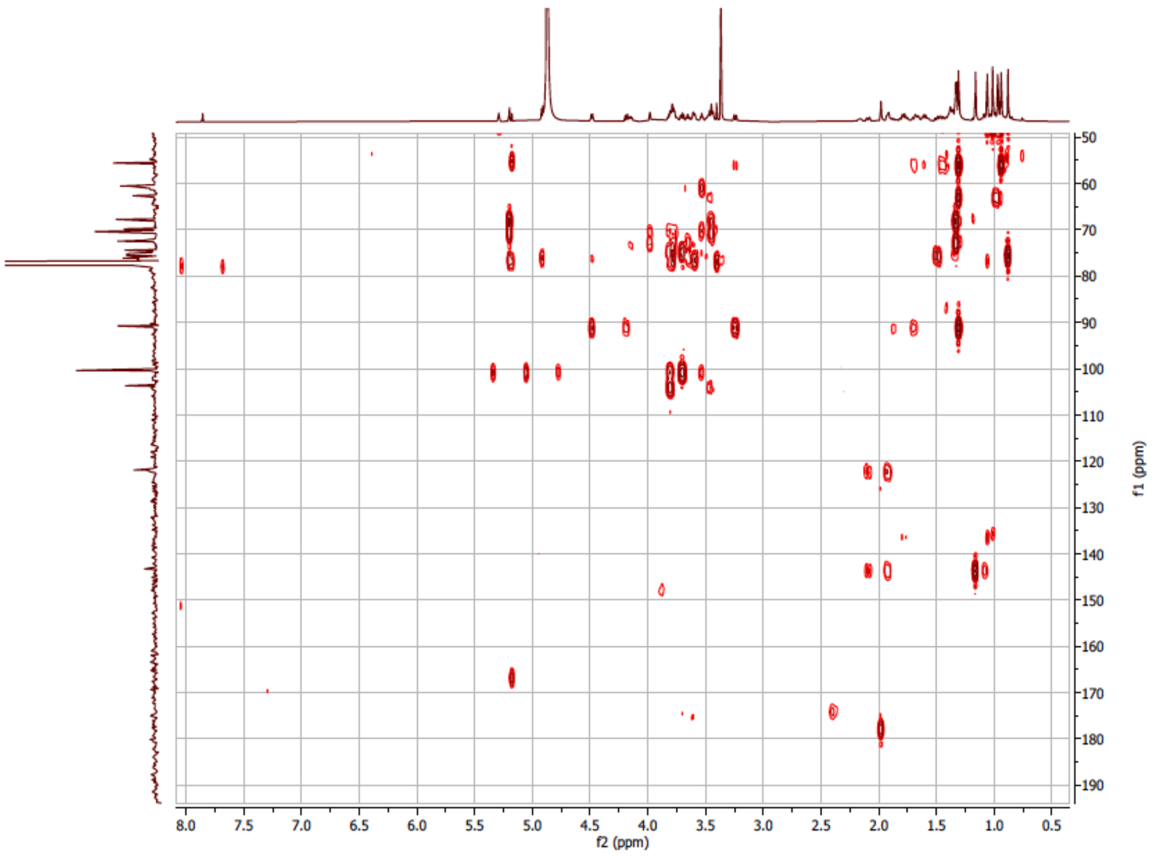

Supplementary figure S10: HMBC Spectrum of Compound **2** (400 MHz, CD3OD)

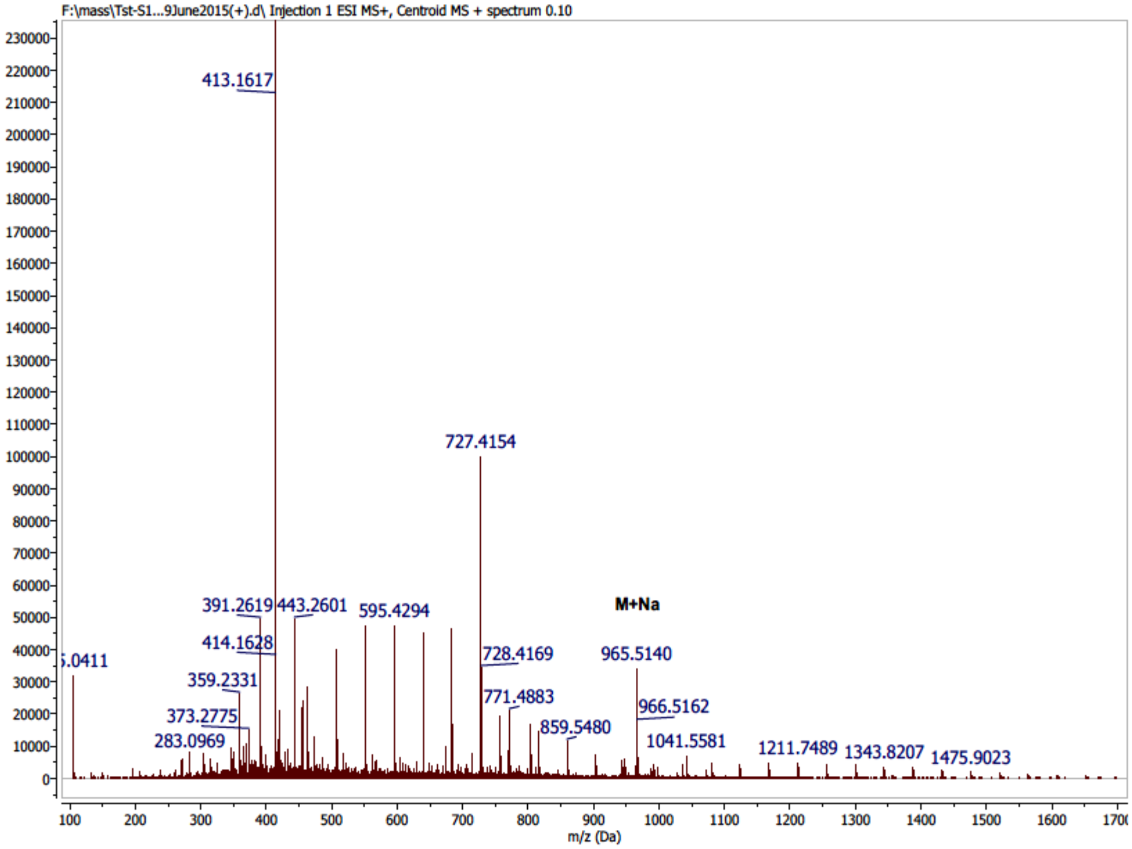

Supplementary figure S11: HRESIMS Spectrum of Compound **2**

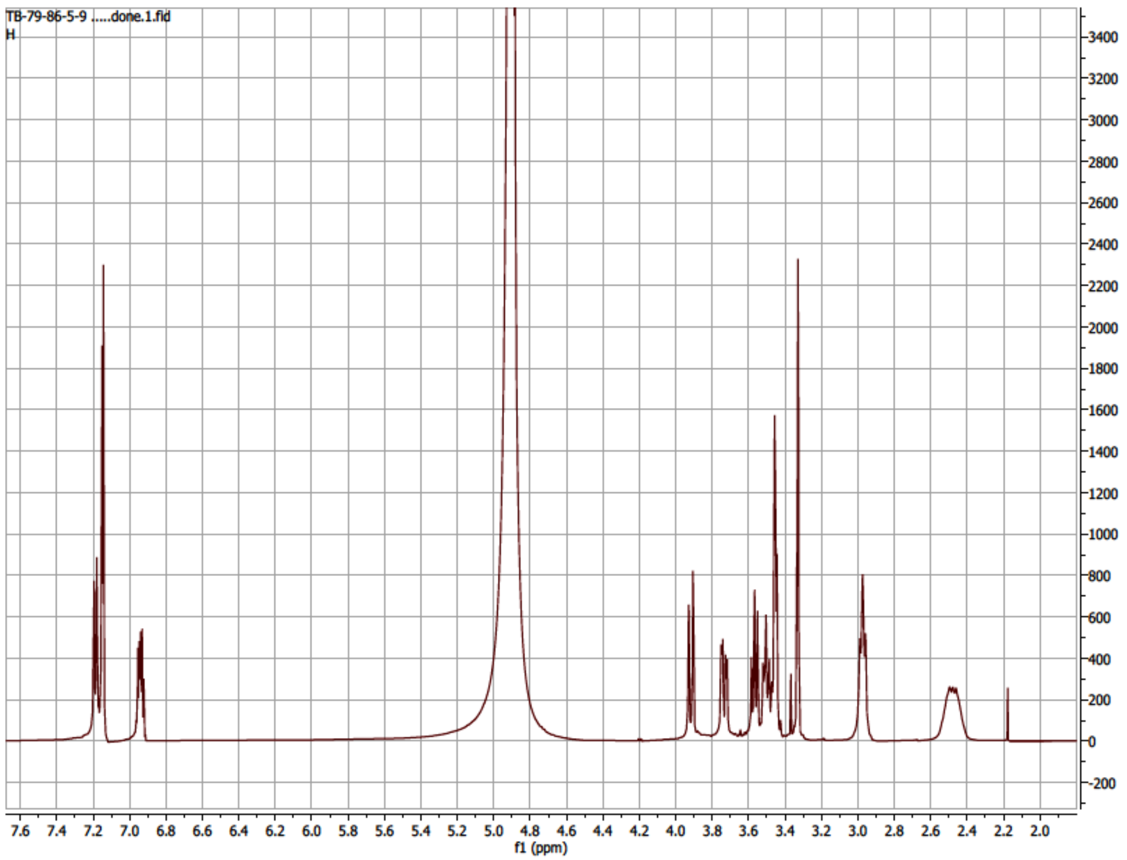

Supplementary figure S12: ^{1}H-NMR Spectrum of Compound **4** (400 MHz, CD3OD)

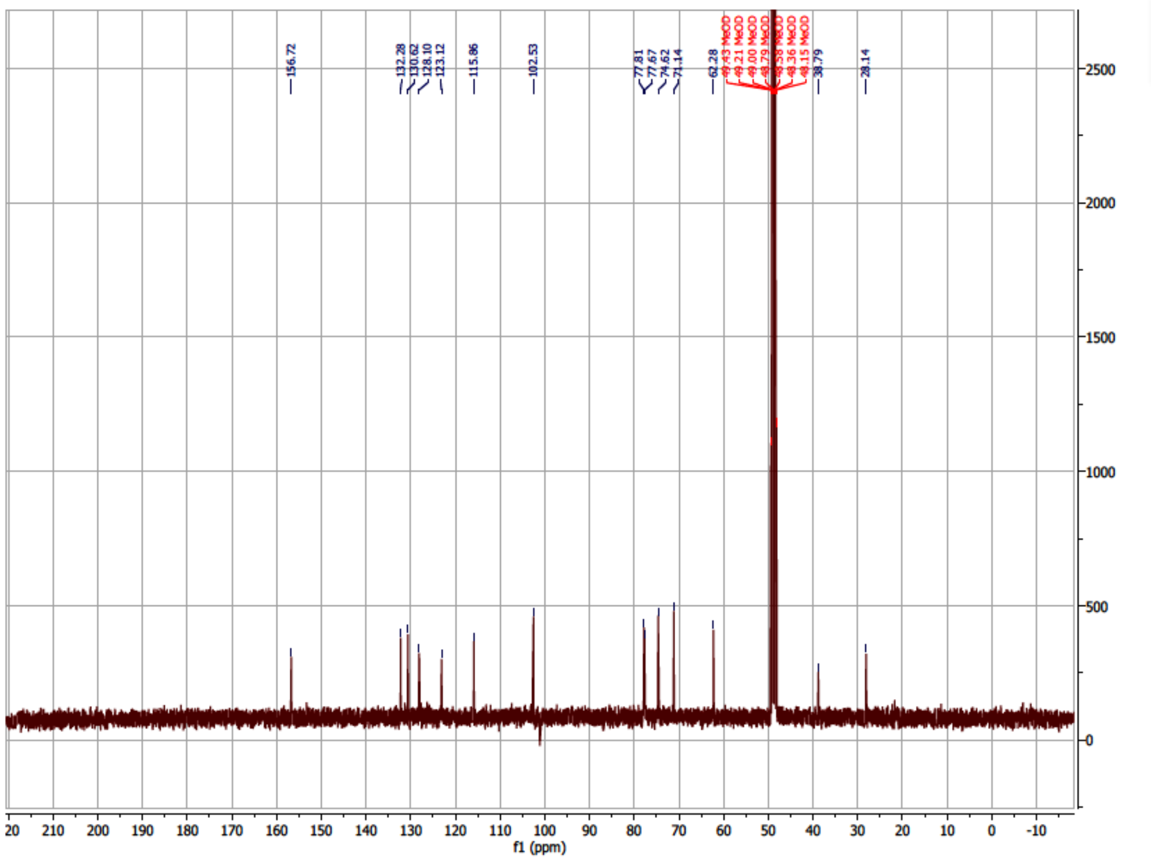

Supplementary figure S13: ^{13}C-NMR Spectrum of Compound **4** (100 MHz, CD3OD)

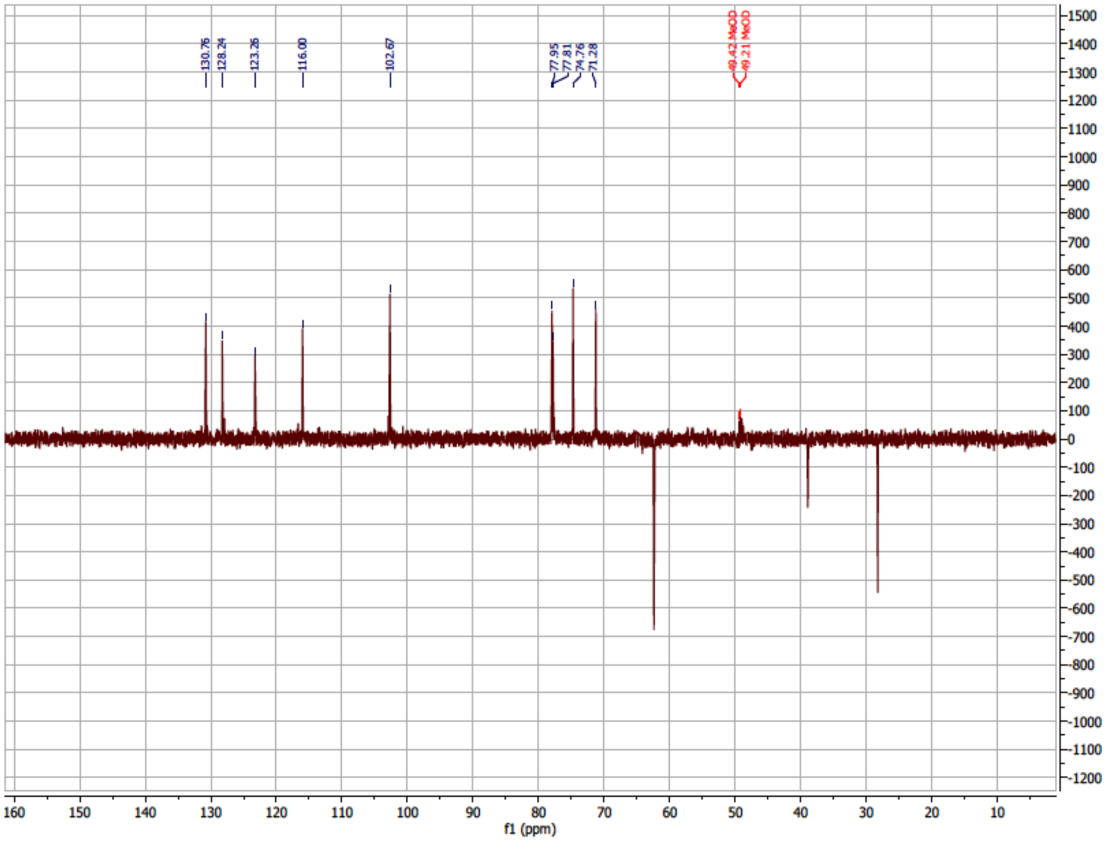

Supplementary figure S14: DEPT Spectrum of Compound **4** in CD3OD

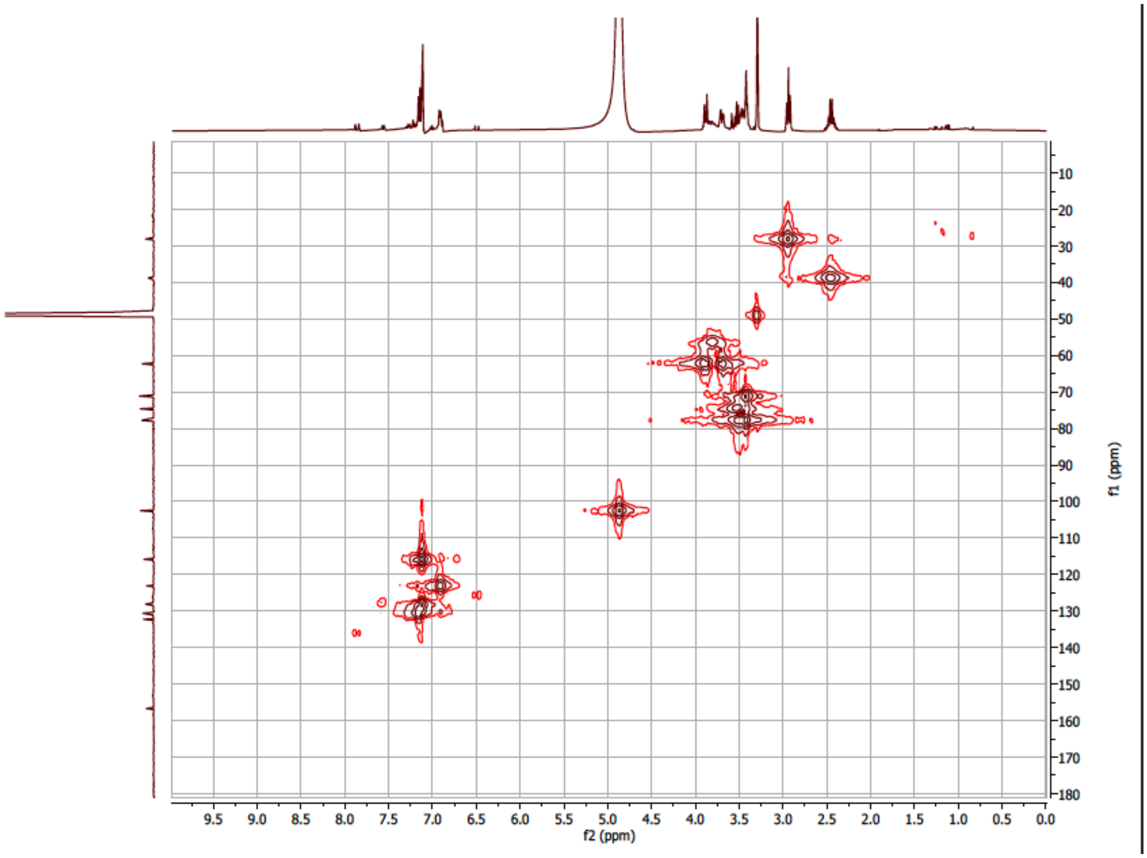

Supplementary figure S15: HMQC Spectrum of Compound **4** (400 MHz, CD3OD)

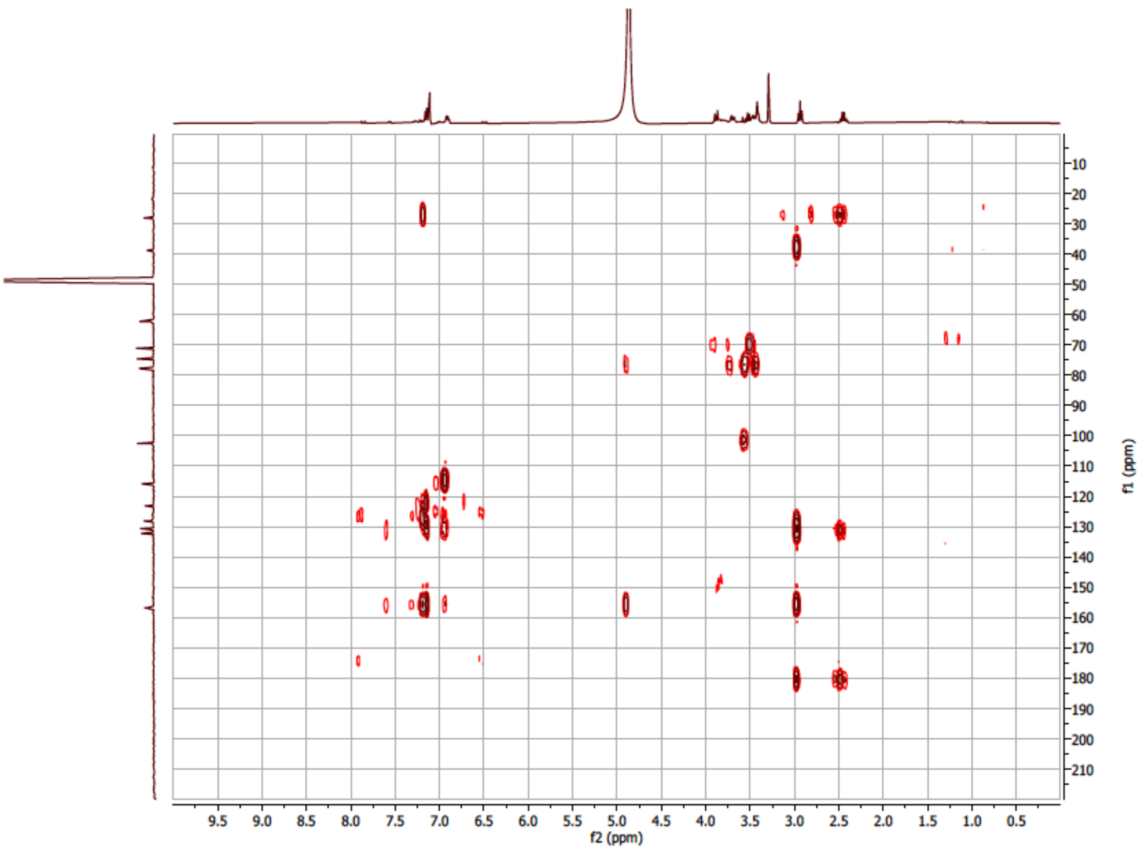

Supplementary figure S16: HMBC Spectrum of Compound **4** (400 MHz, CD3OD)

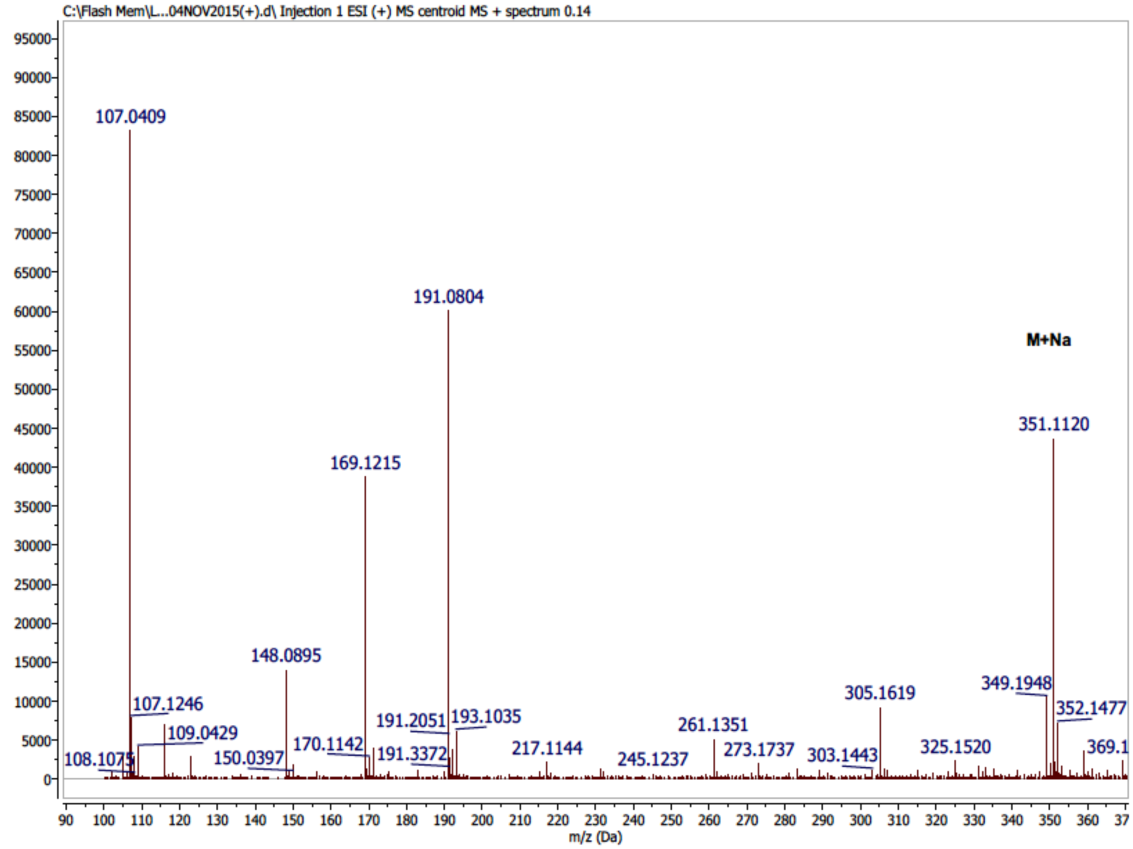

Supplementary figure S17: HRESIMS Spectrum of Compound **4**

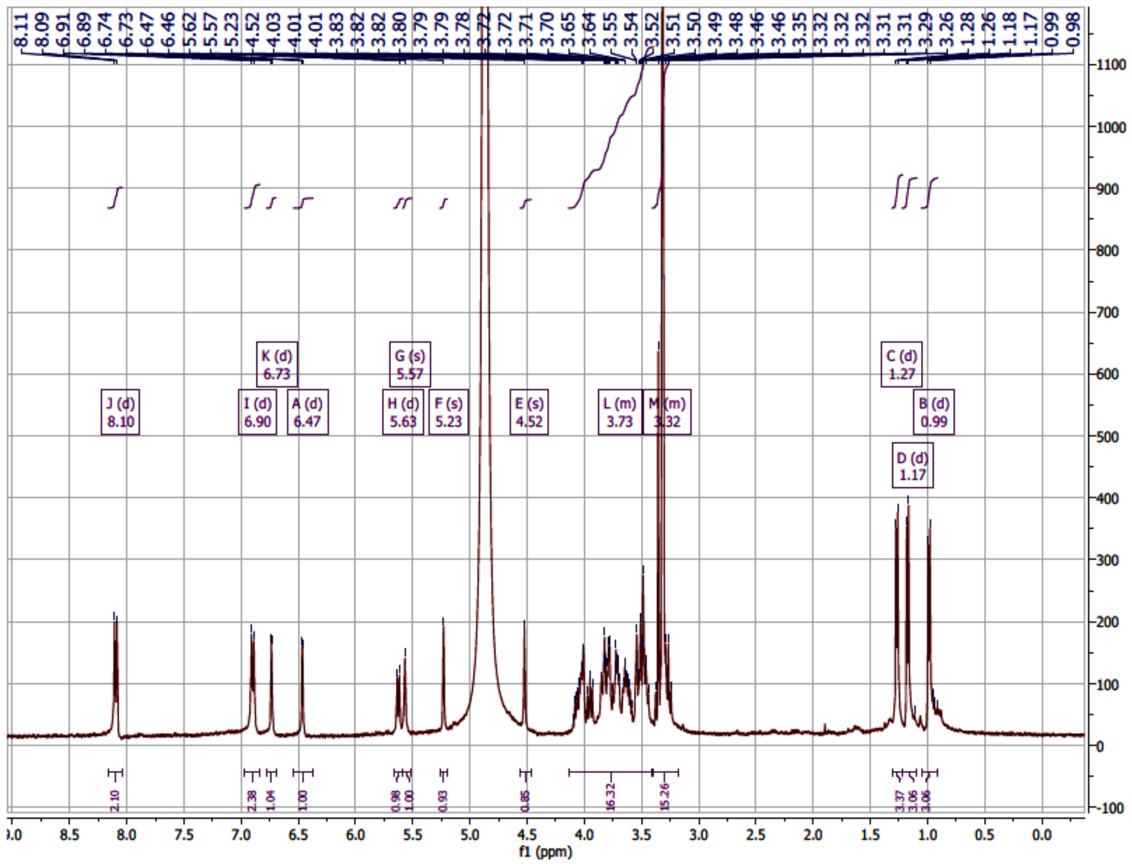

Supplementary figure S18: ^{1}H-NMR Spectrum of Compound **5** (500 MHz, CD3OD)

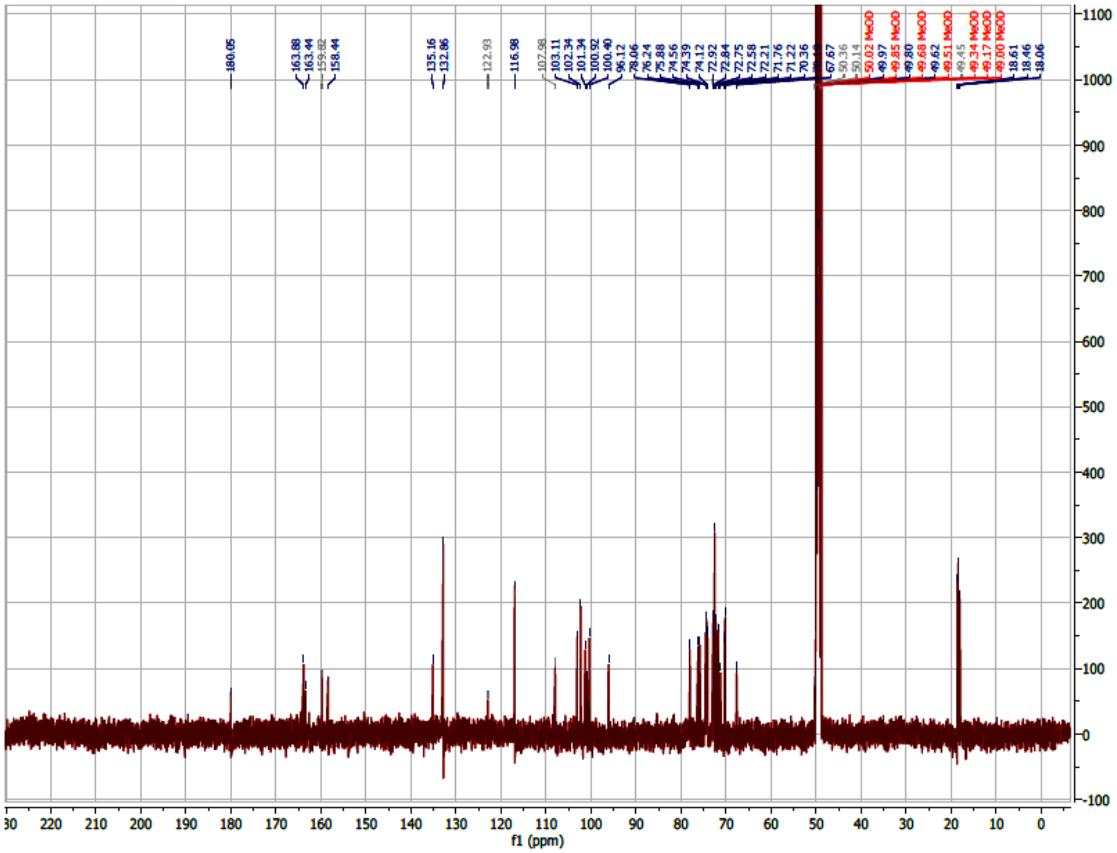

Supplementary figure S19: ^{13}C-NMR Spectrum of Compound **5** (125 MHz, CD3OD)

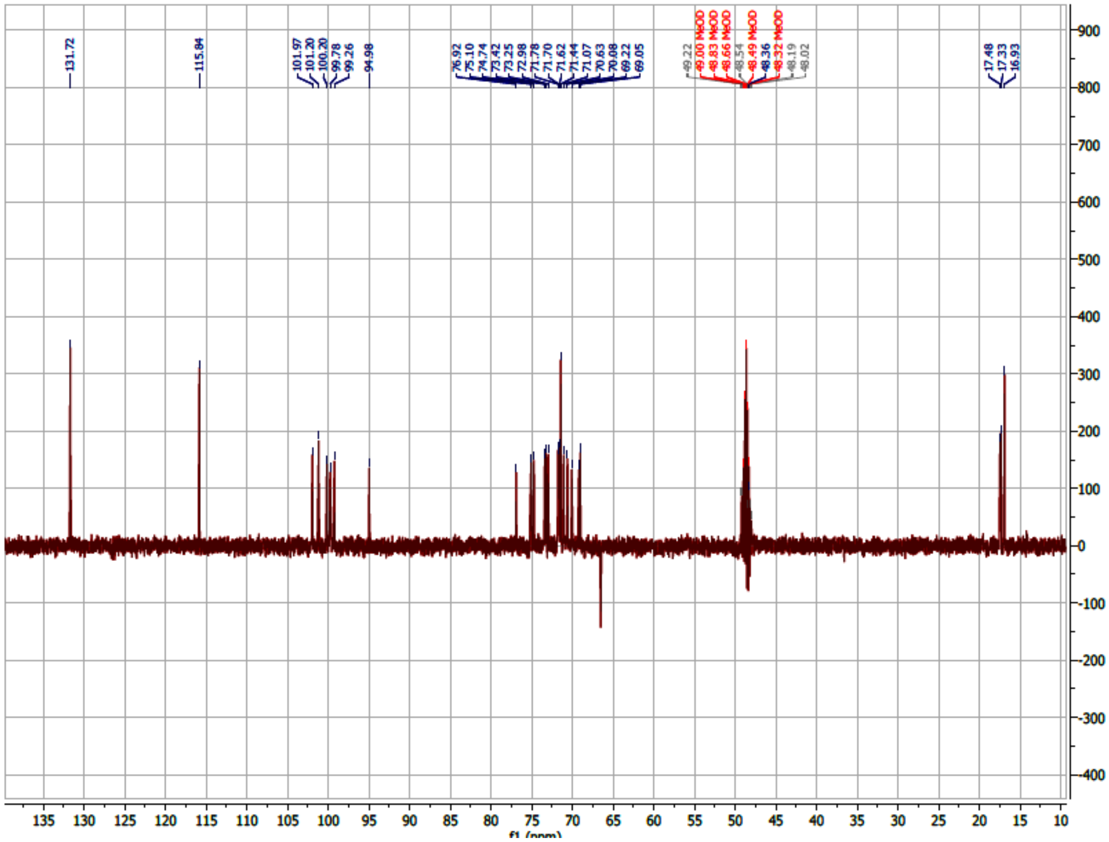

Supplementary figure S20: DEPT Spectrum of Compound **5** in CD3OD

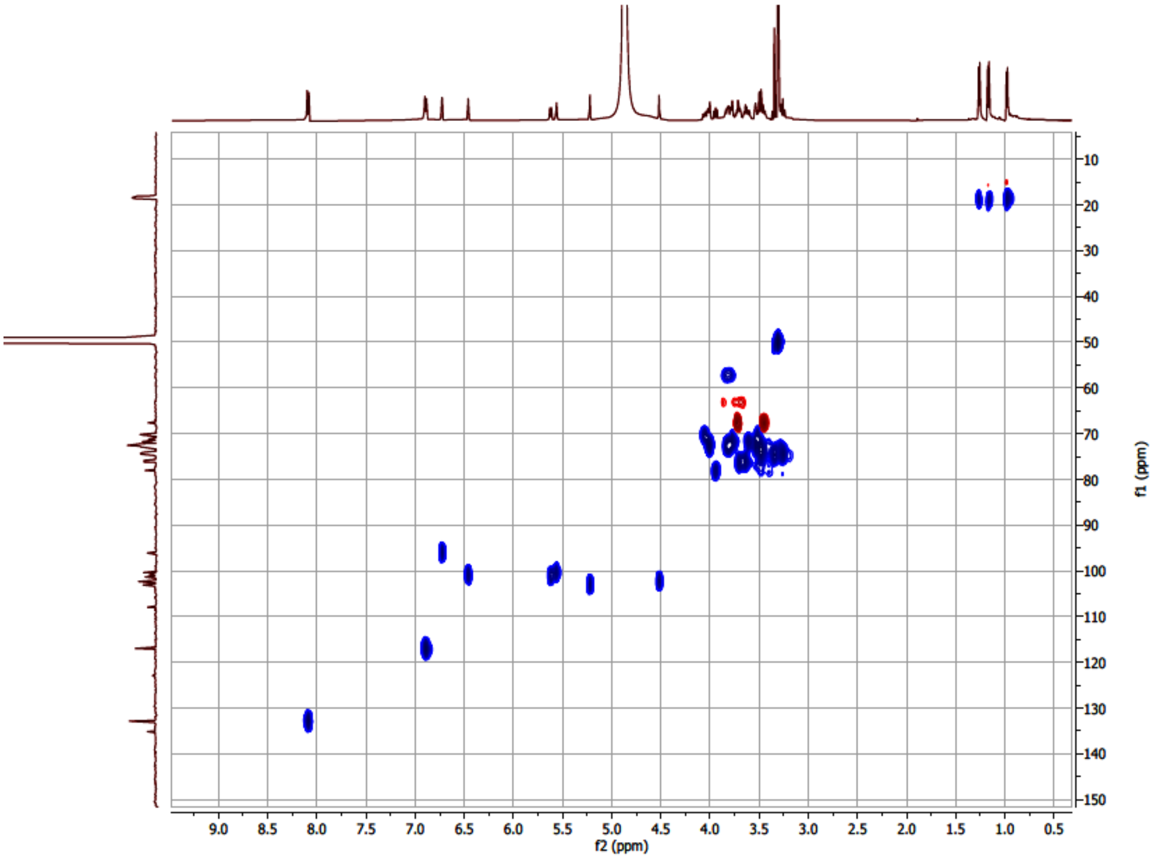

Supplementary figure S21: HSQC Spectrum of Compound **5** (500 MHz, CD3OD)

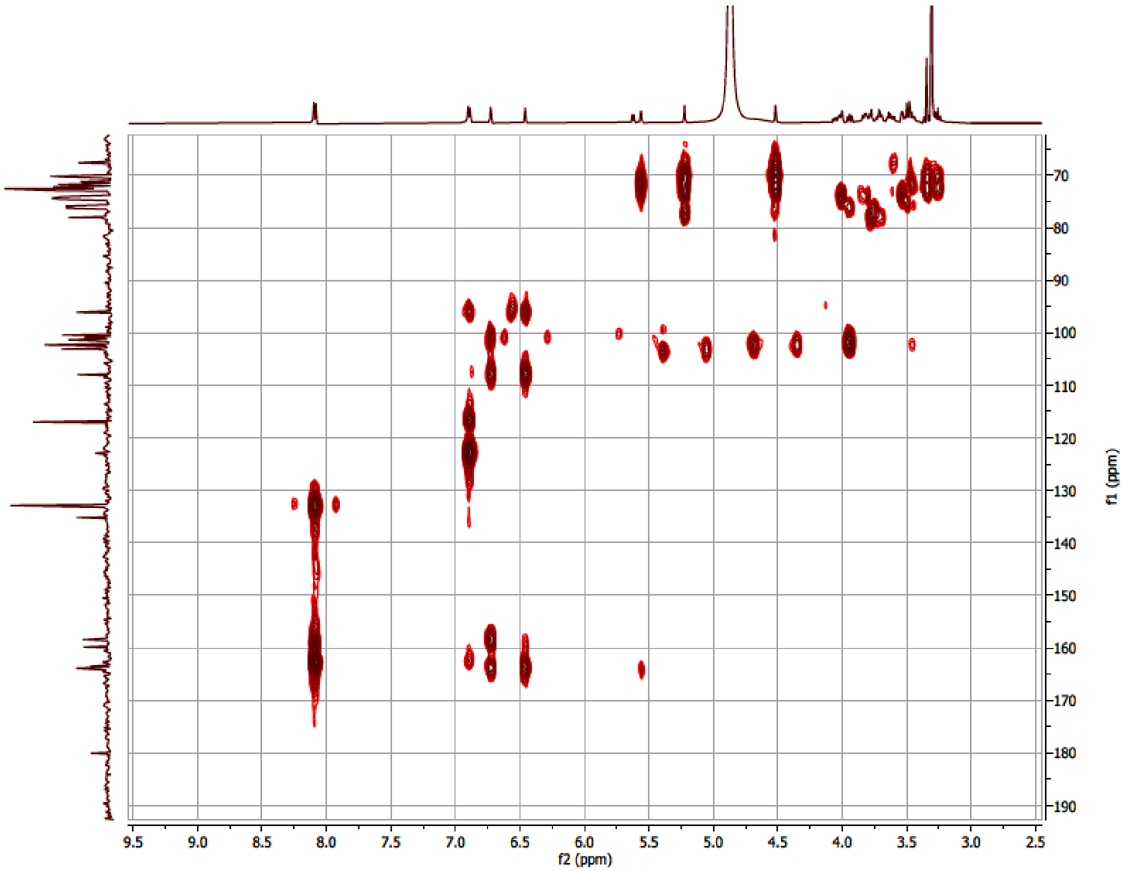

Supplementary figure S22: HMBC Spectrum of Compound **5** (500 MHz, CD3OD)

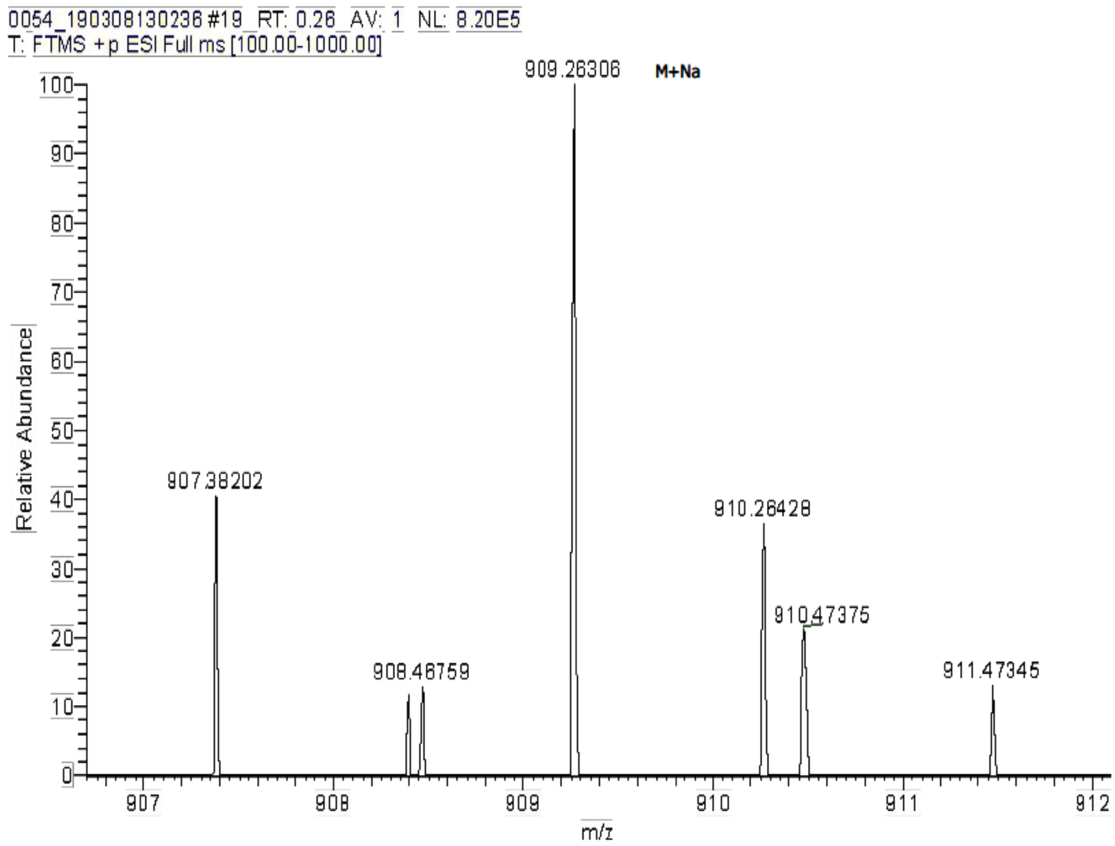

Supplementary figure S23: HRESIMS Spectrum of Compound **5**

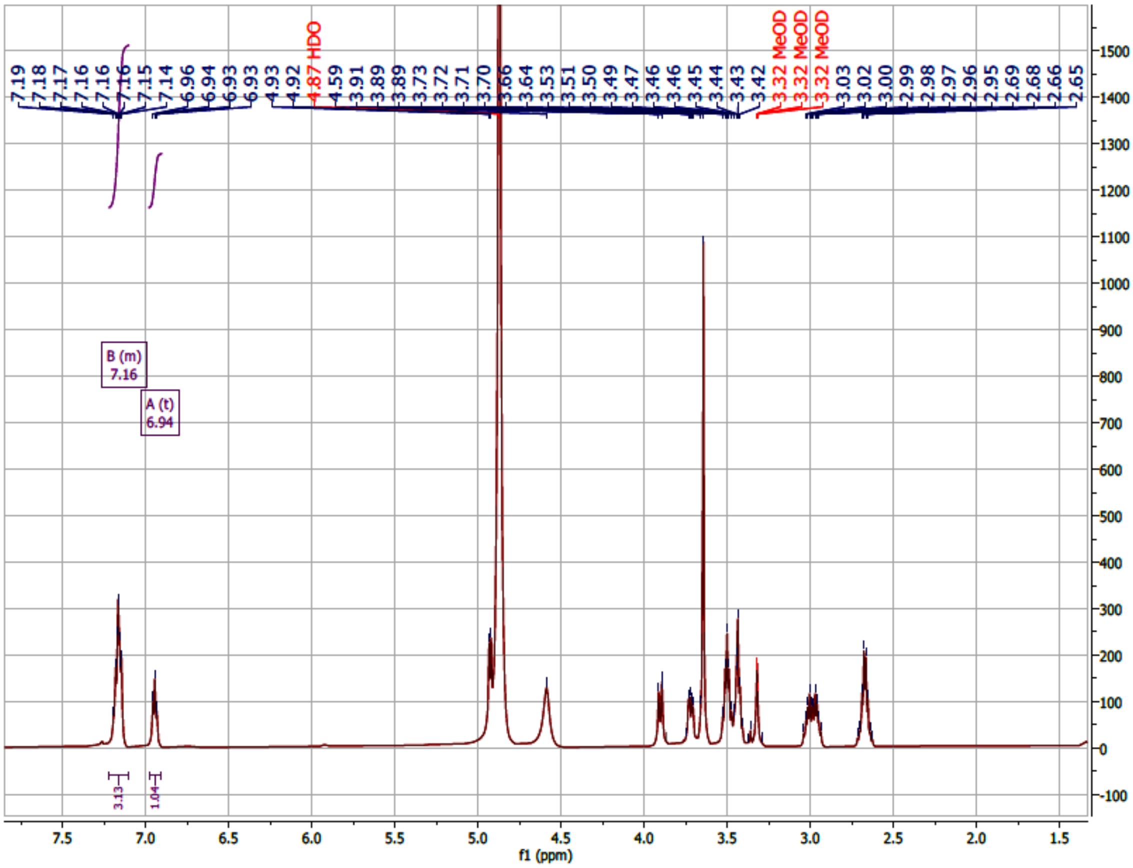

Supplementary figure S24: ^{1}H-NMR Spectrum of Compound **6** (600 MHz, CD3OD)

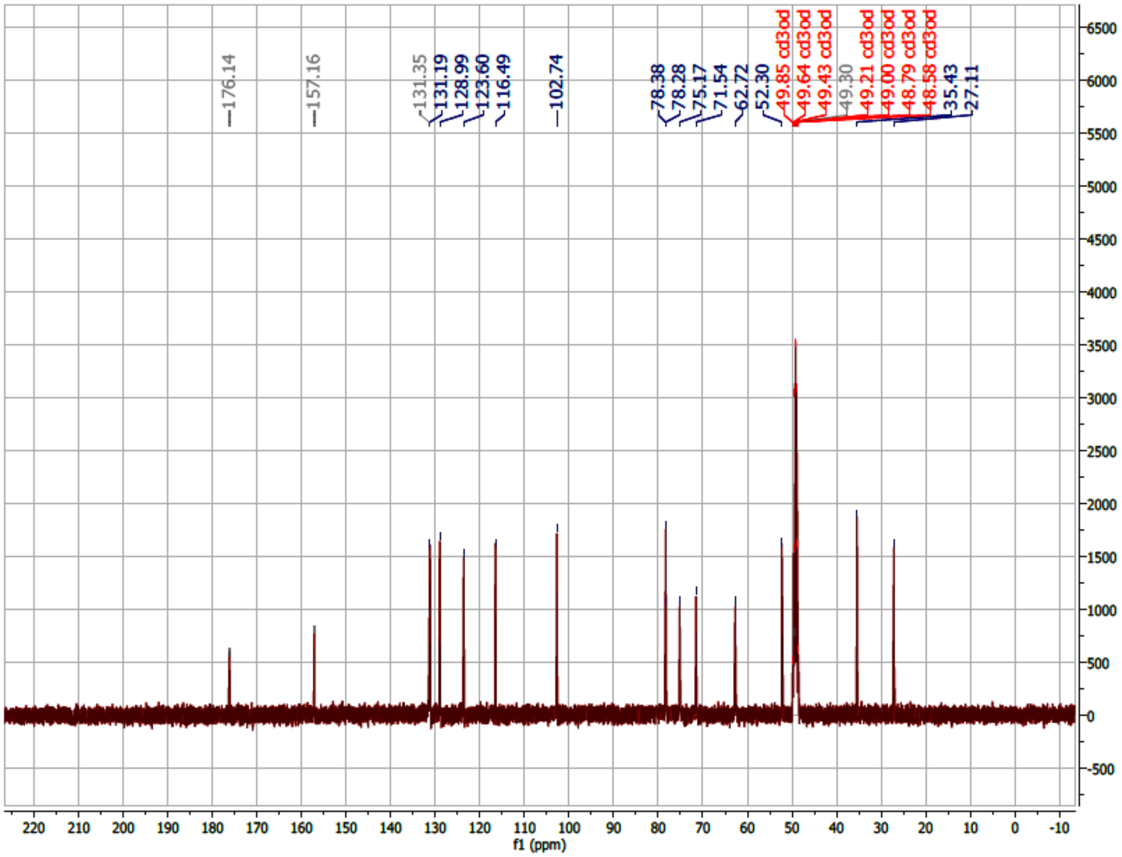

Supplementary figure S25: ^{13}C-NMR Spectrum of Compound **6** (600 MHz, CD3OD)

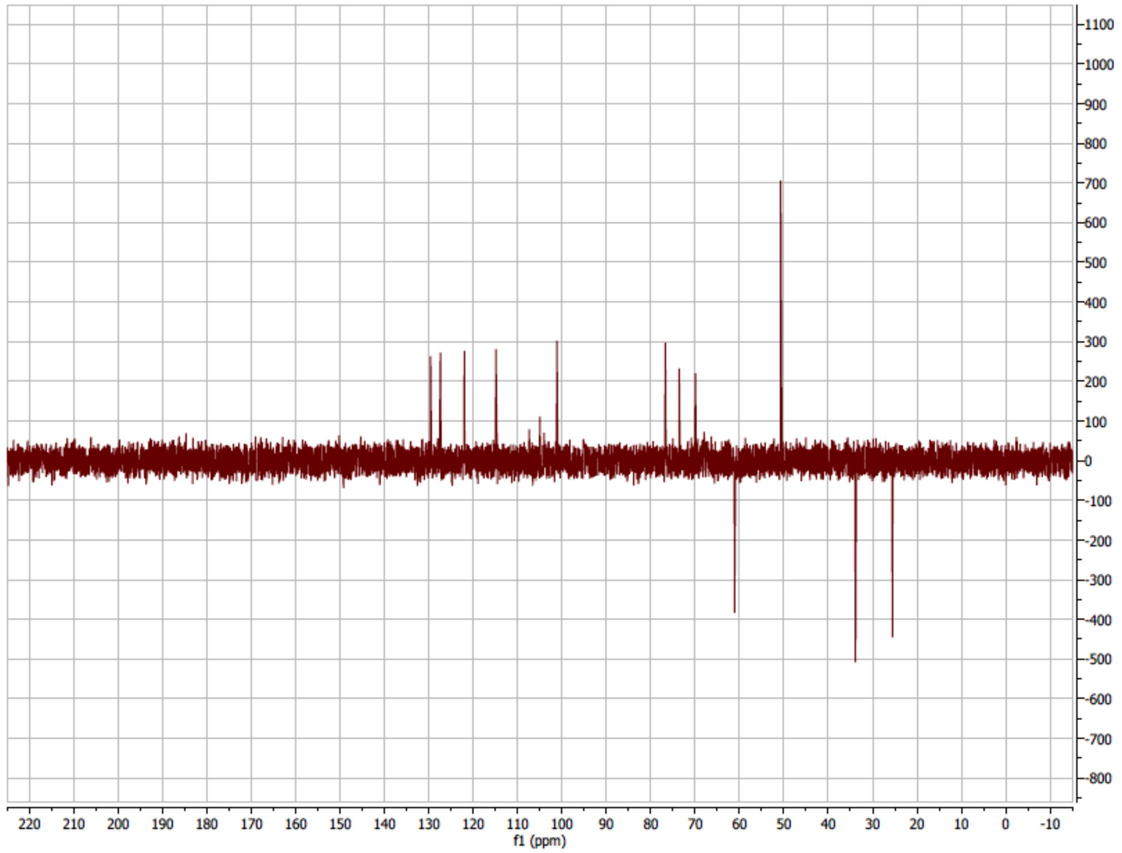

Supplementary figure S26: DEPT Spectrum of Compound **6** in CD3OD

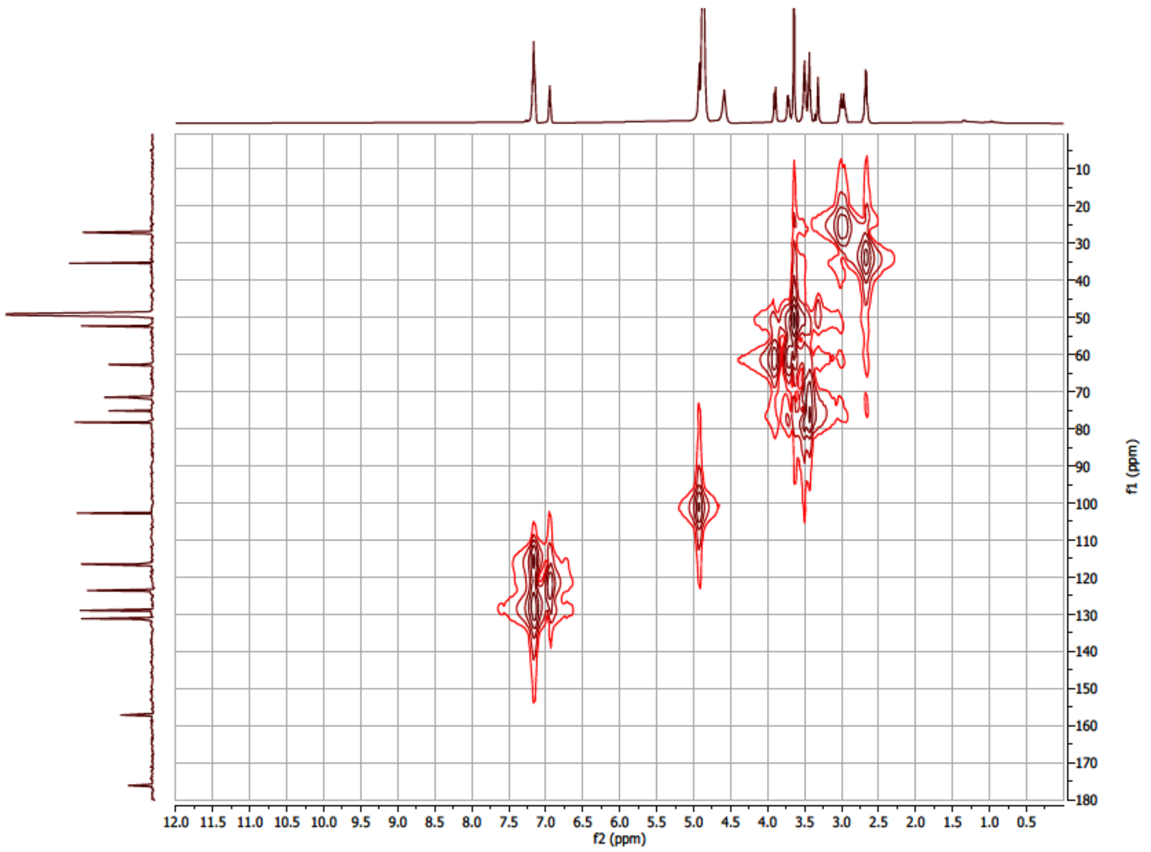

Supplementary figure S27: HMQC Spectrum of Compound **6** (600 MHz, CD3OD)

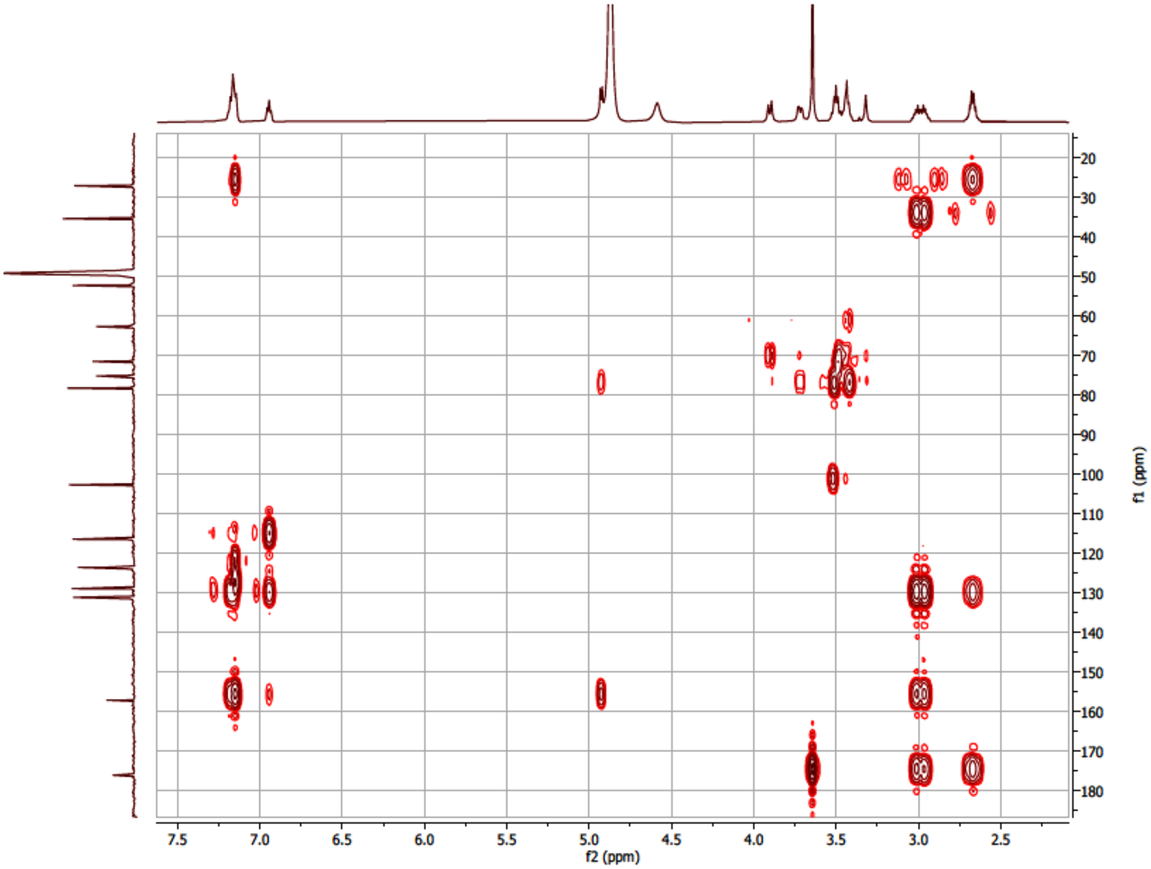

Supplementary figure S28: HMBC Spectrum of Compound **6** (600 MHz, CD3OD)

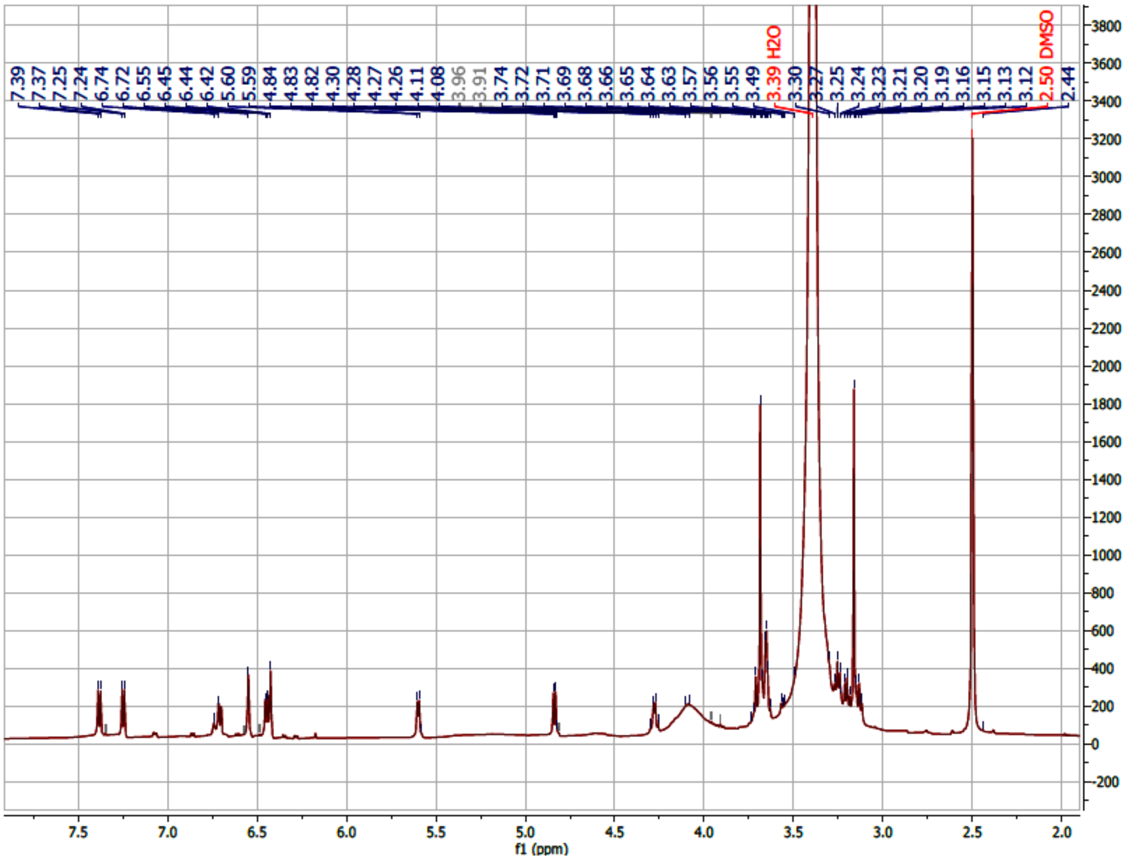

Supplementary figure S29: ^{1}H-NMR Spectrum of Compound **7** (400 MHz, DMSO-d_6)

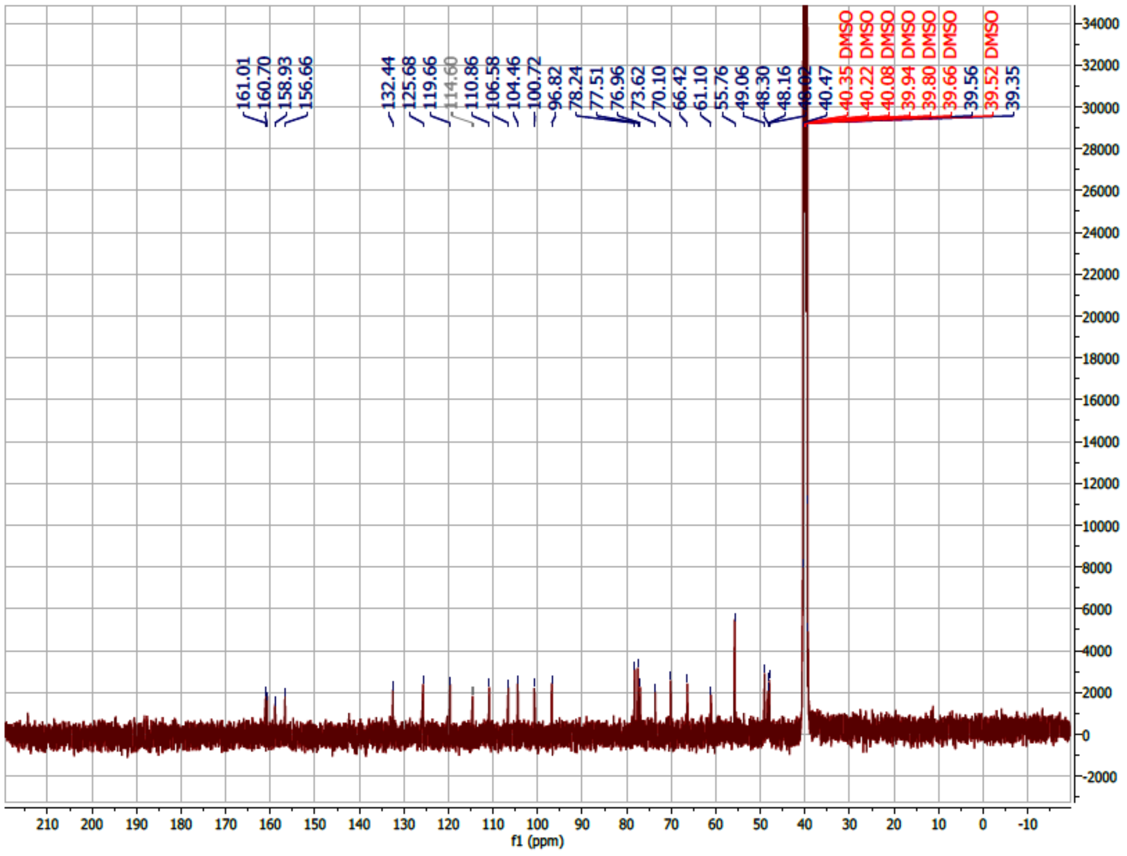

Supplementary figure S30: ^{13}C-NMR Spectrum of Compound **7** (100 MHz, DMSO-d_6)

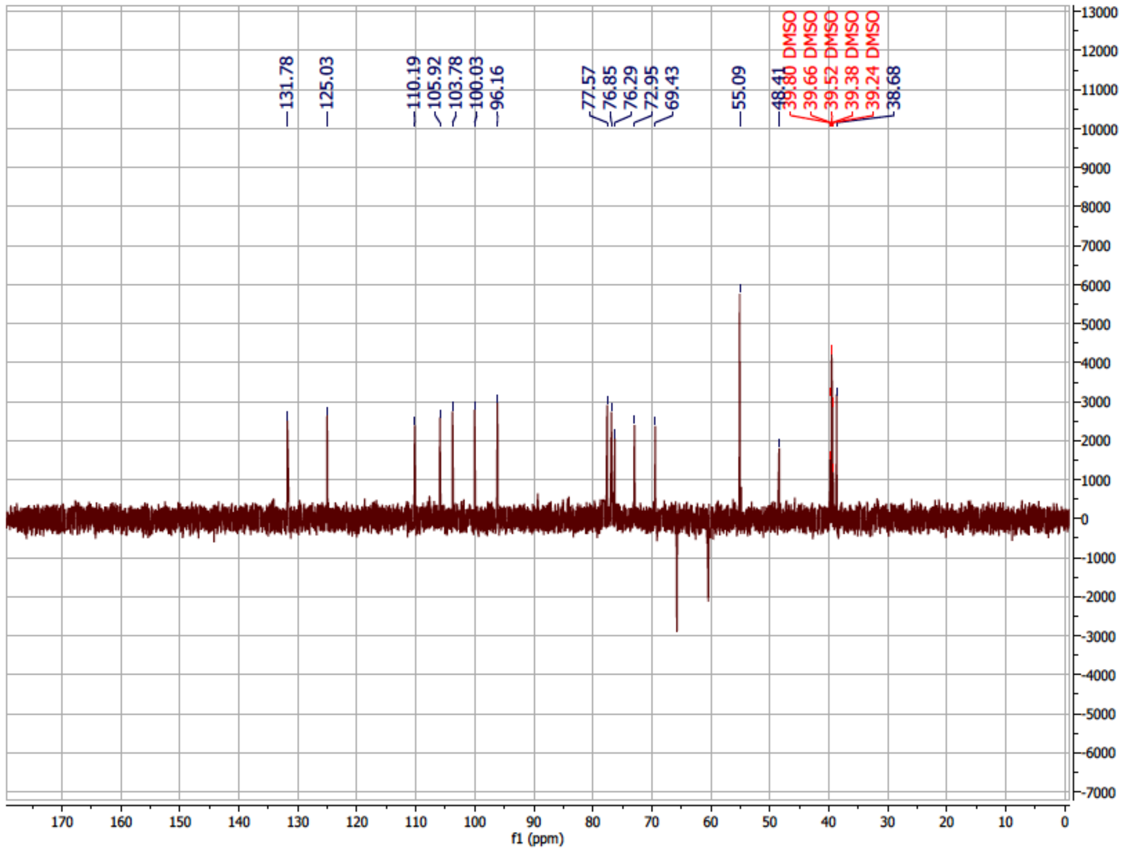

Supplementary figure S31: DEPT Spectrum of Compound **7** in DMSO-d_6

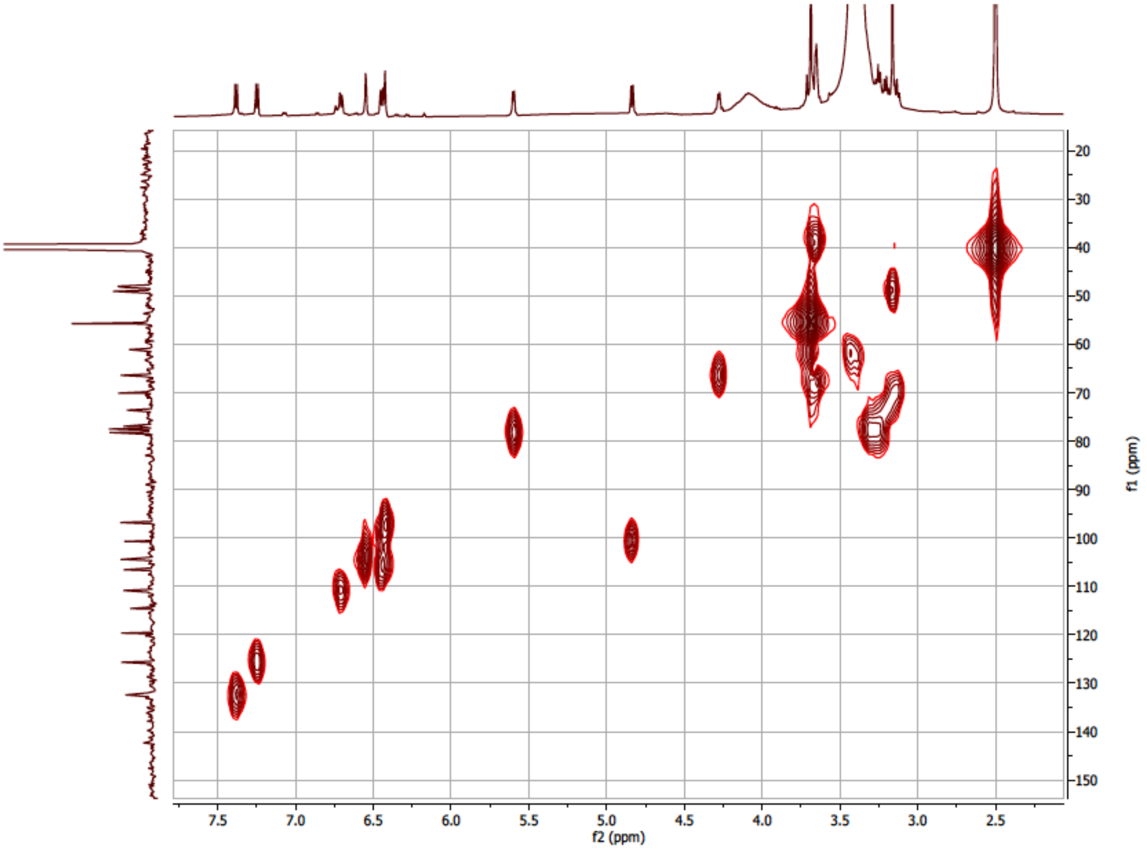

Supplementary figure S32: HMQC Spectrum of Compound **7** (400 MHz, DMSO-d_6)

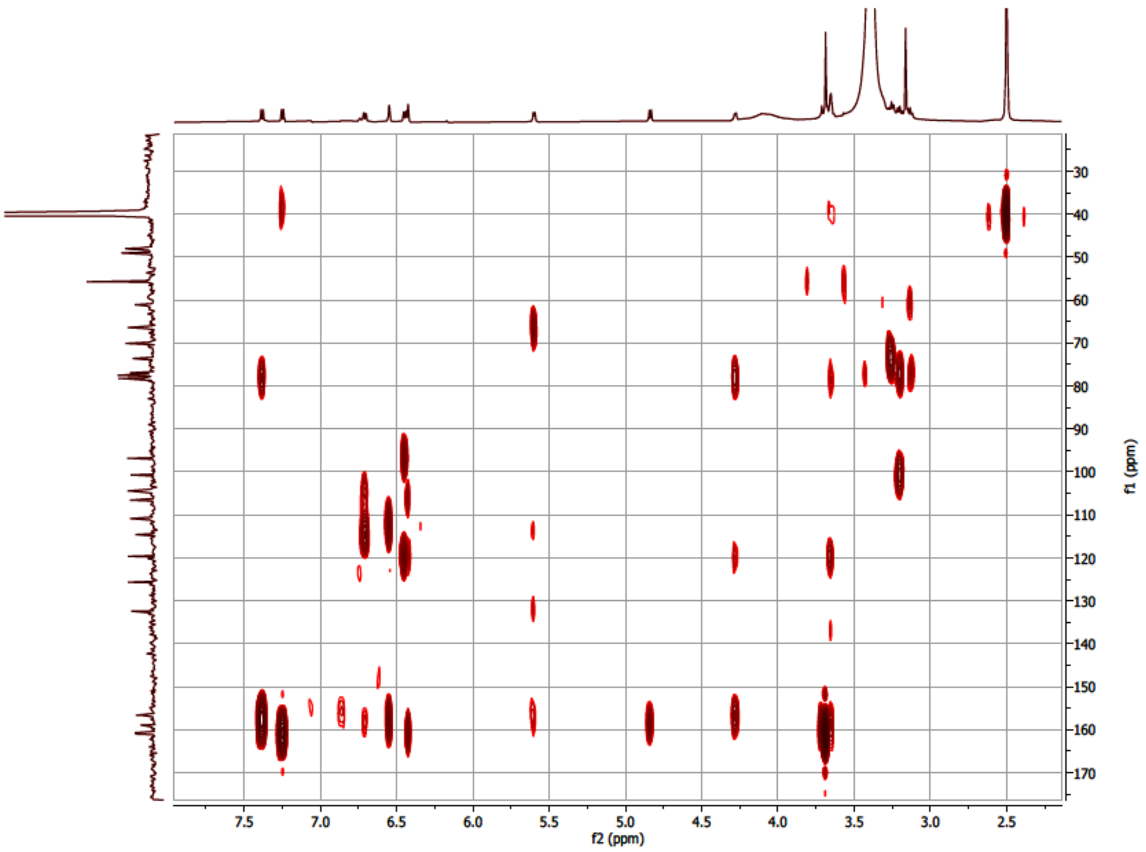

Supplementary figure S33: HMBC Spectrum of Compound **7** (400 MHz, DMSO-d_6)

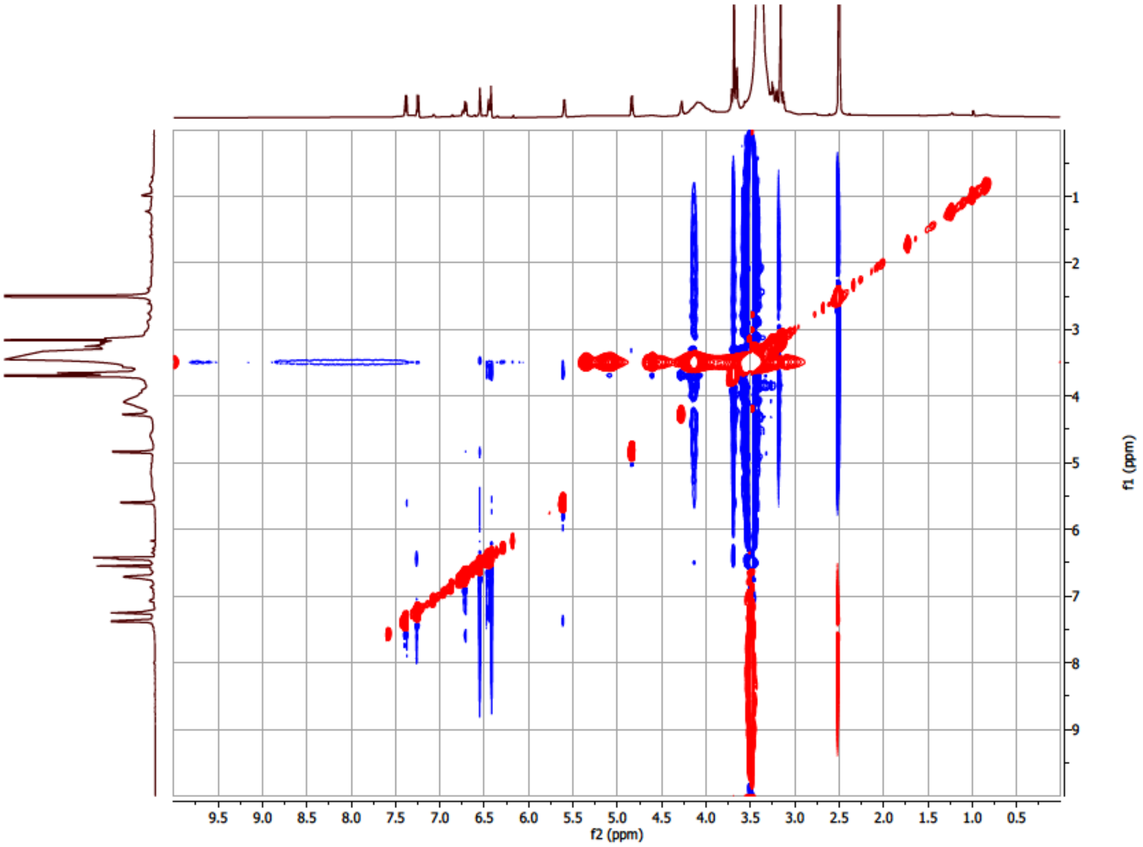

Supplementary figure S34: NOESY Spectrum of Compound **7**

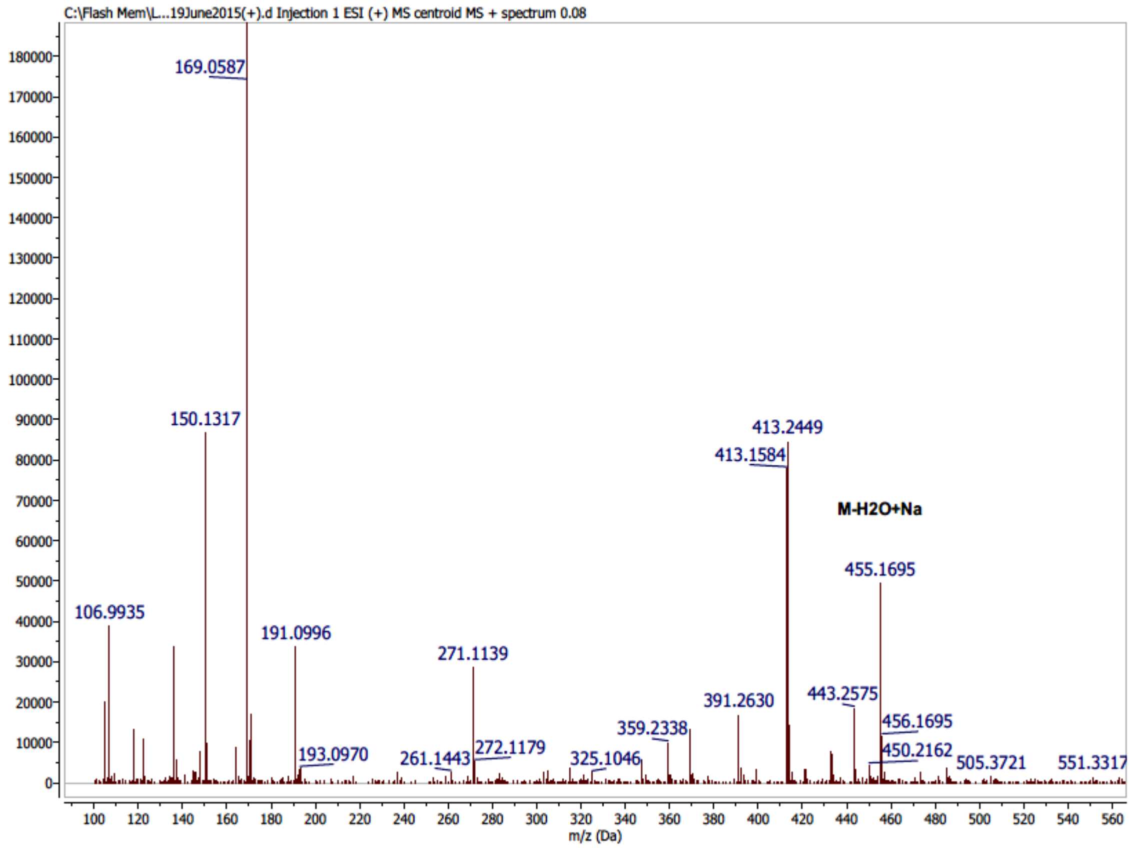

Supplementary figure S35: HRESIMS Spectrum of Compound **7**

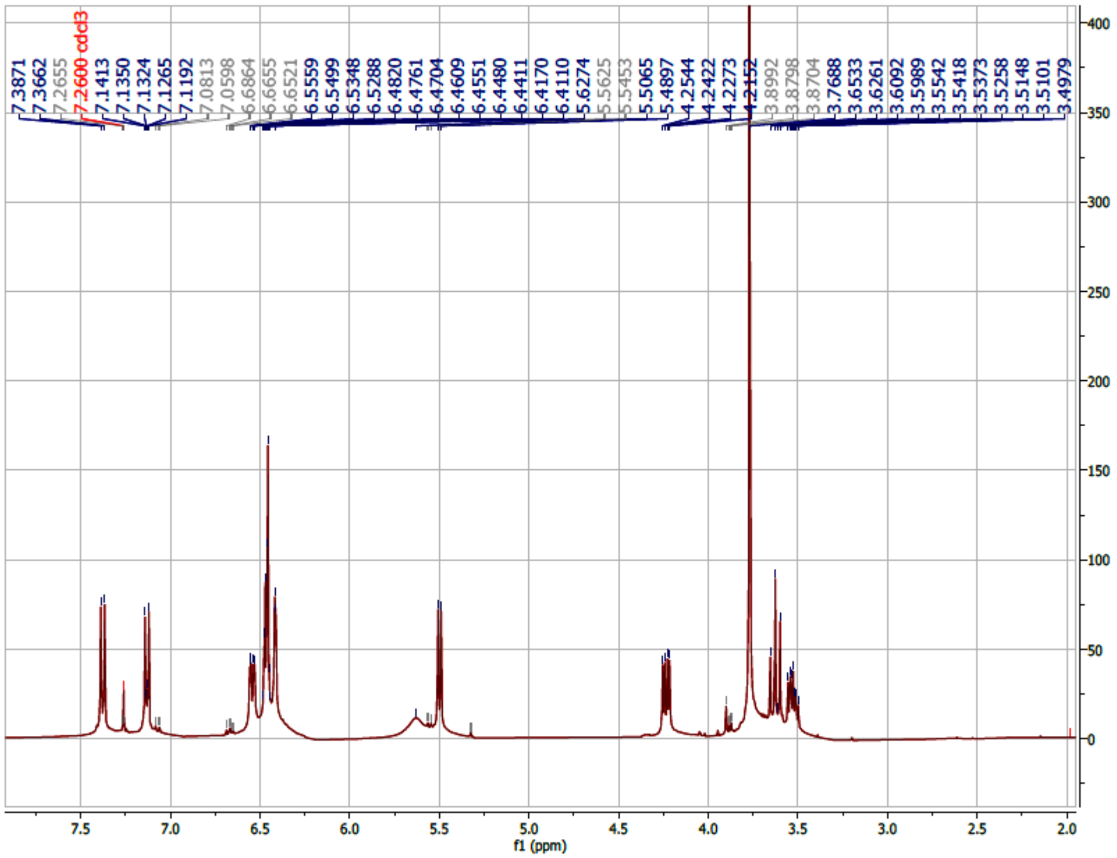

Supplementary figure S36: ^{1}H-NMR Spectrum of Compound **8** (400 MHz, $CDCl_3$)

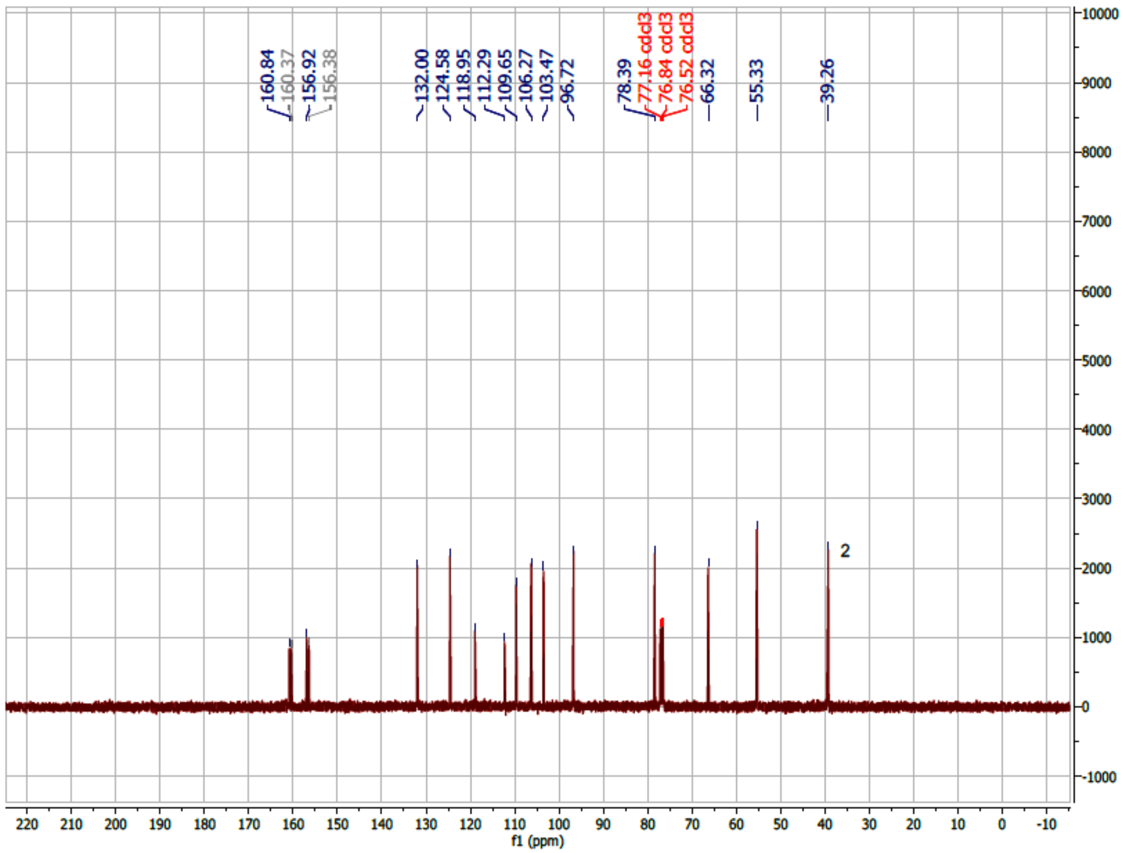

Supplementary figure S37: ^{13}C-NMR Spectrum of Compound **8** (100 MHz, $CDCl_3$)

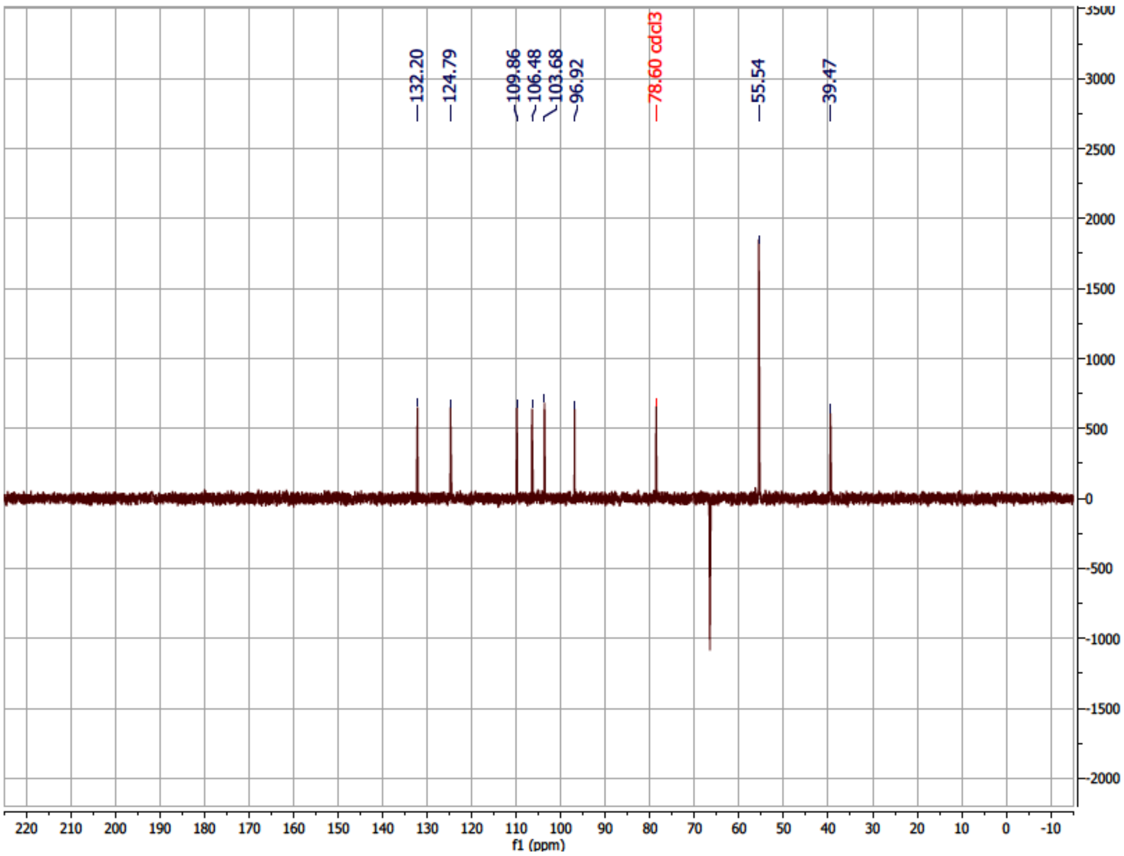

Supplementary figure S38: DEPT Spectrum of Compound **8** in $CDCl_3$

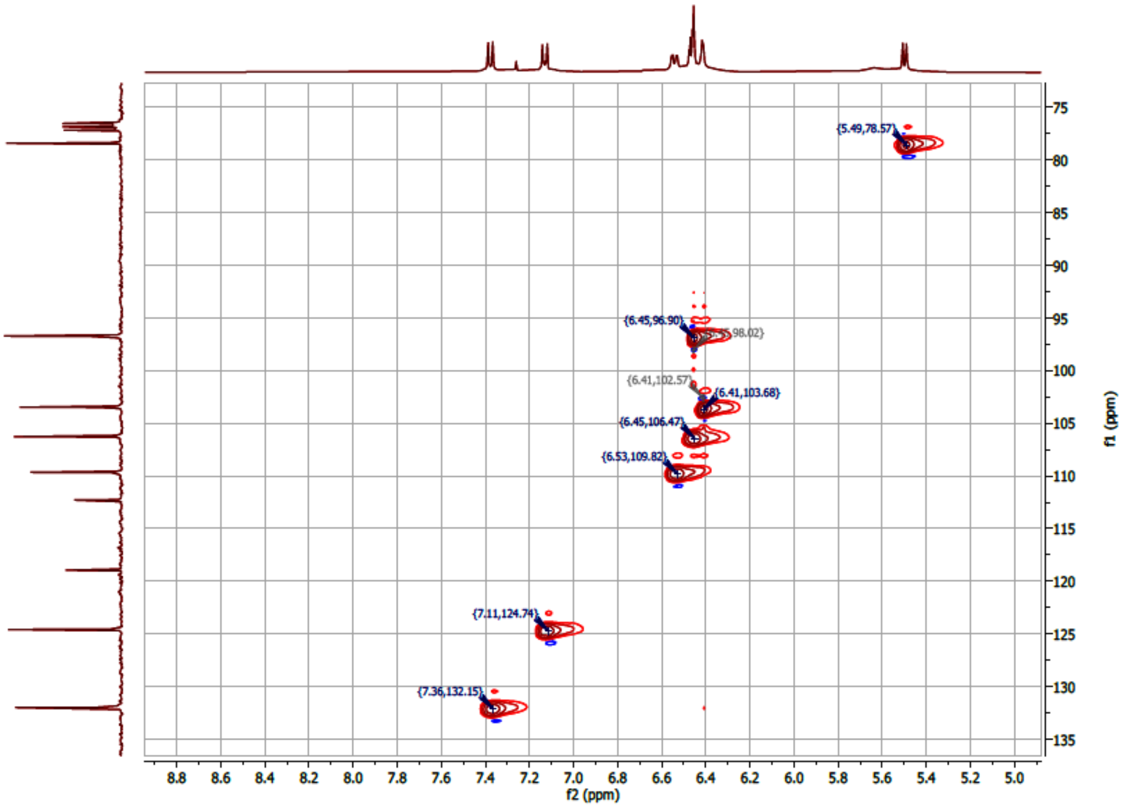

Supplementary figure S39: HMQC Spectrum of Compound **8** (400 MHz, $CDCl_3$)

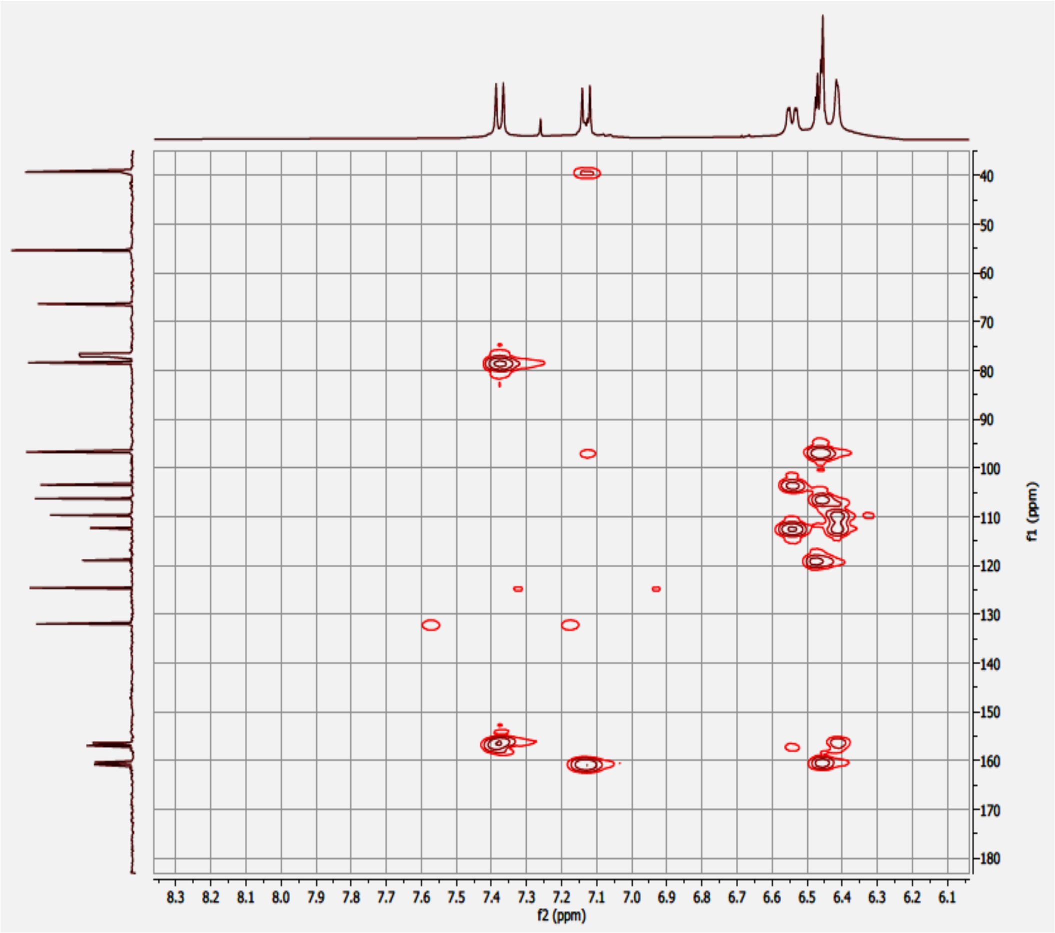

Supplementary figure S40: HMBC Spectrum of Compound **8** (400 MHz, $CDCl_3$)

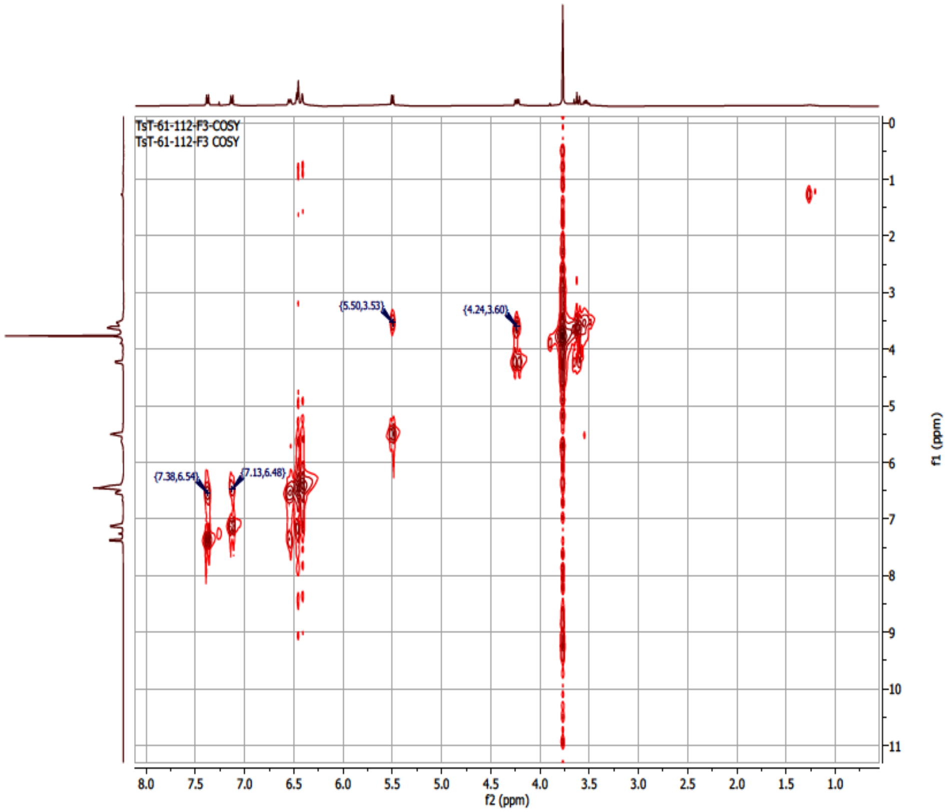

Supplementary figure S41: COSY Spectrum of Compound **8** (400 MHz, $CDCl_3$)

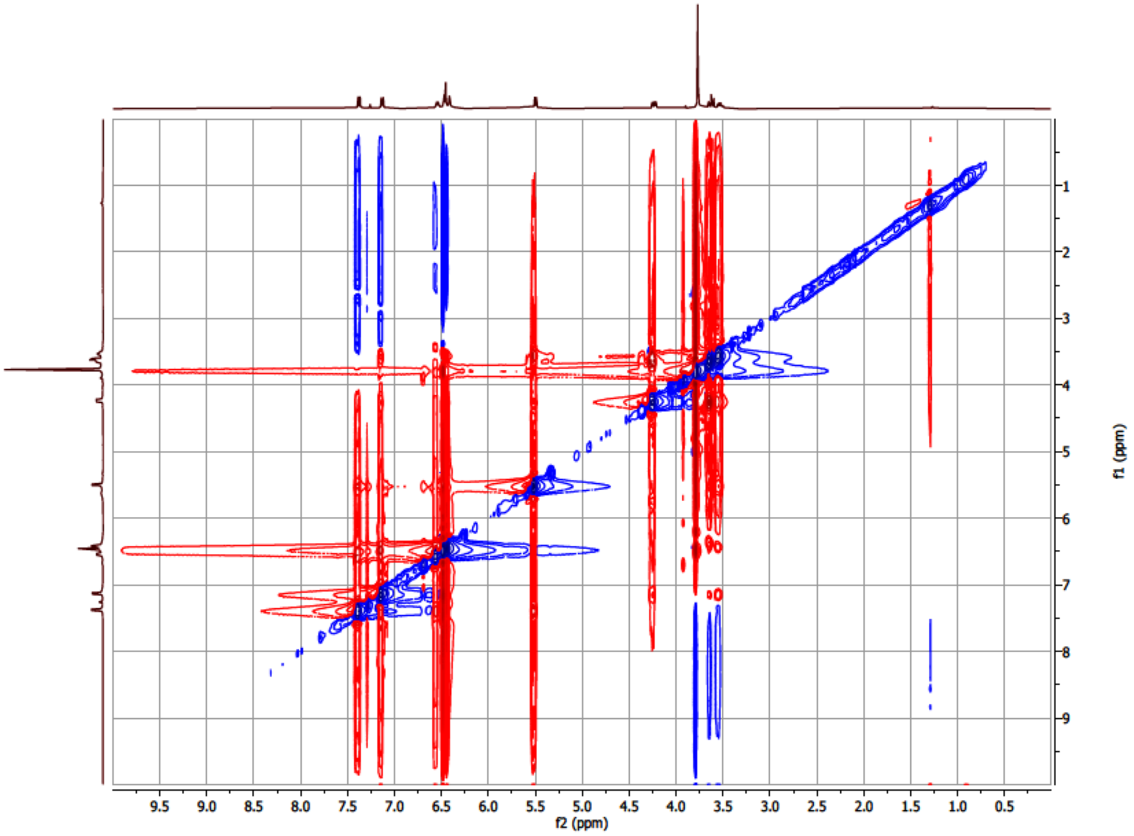

Supplementary figure S42: NOESY Spectrum of Compound **8** (400 MHz, $CDCl_3$)

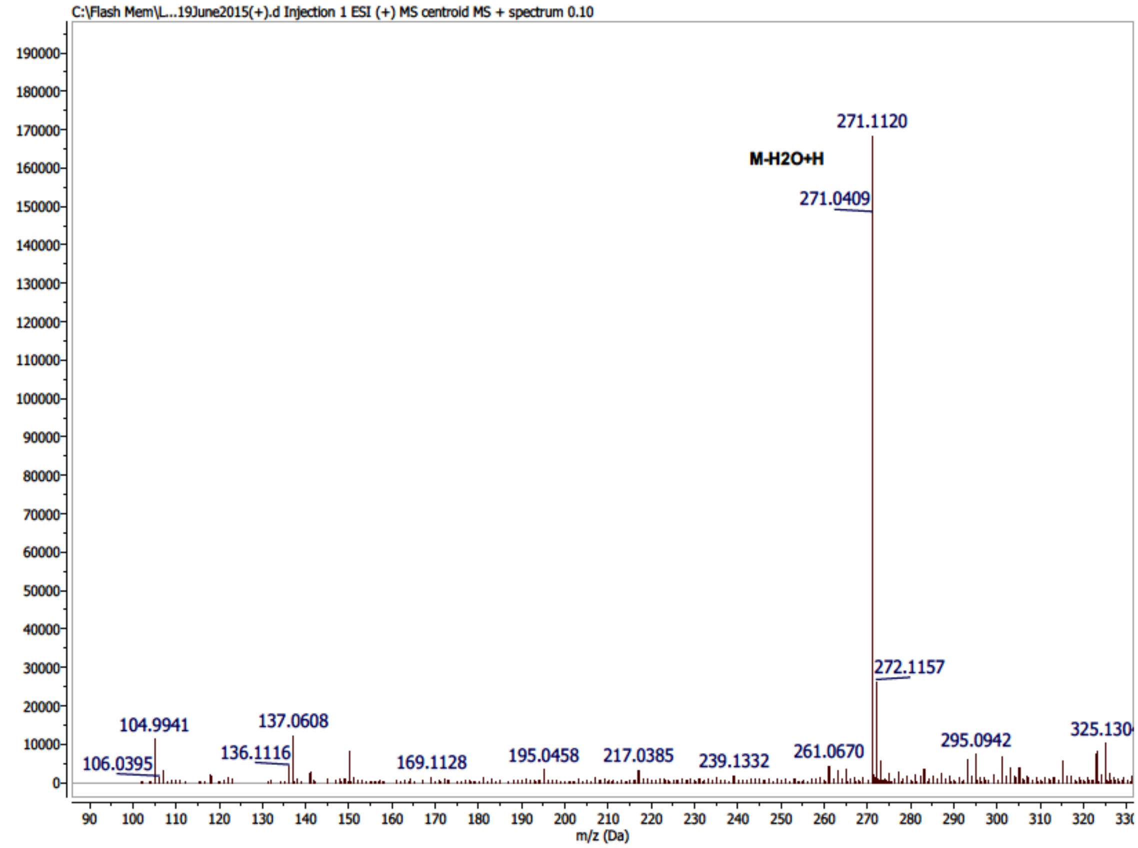

Supplementary figure S43: HRESIMS Spectrum of Compound 8

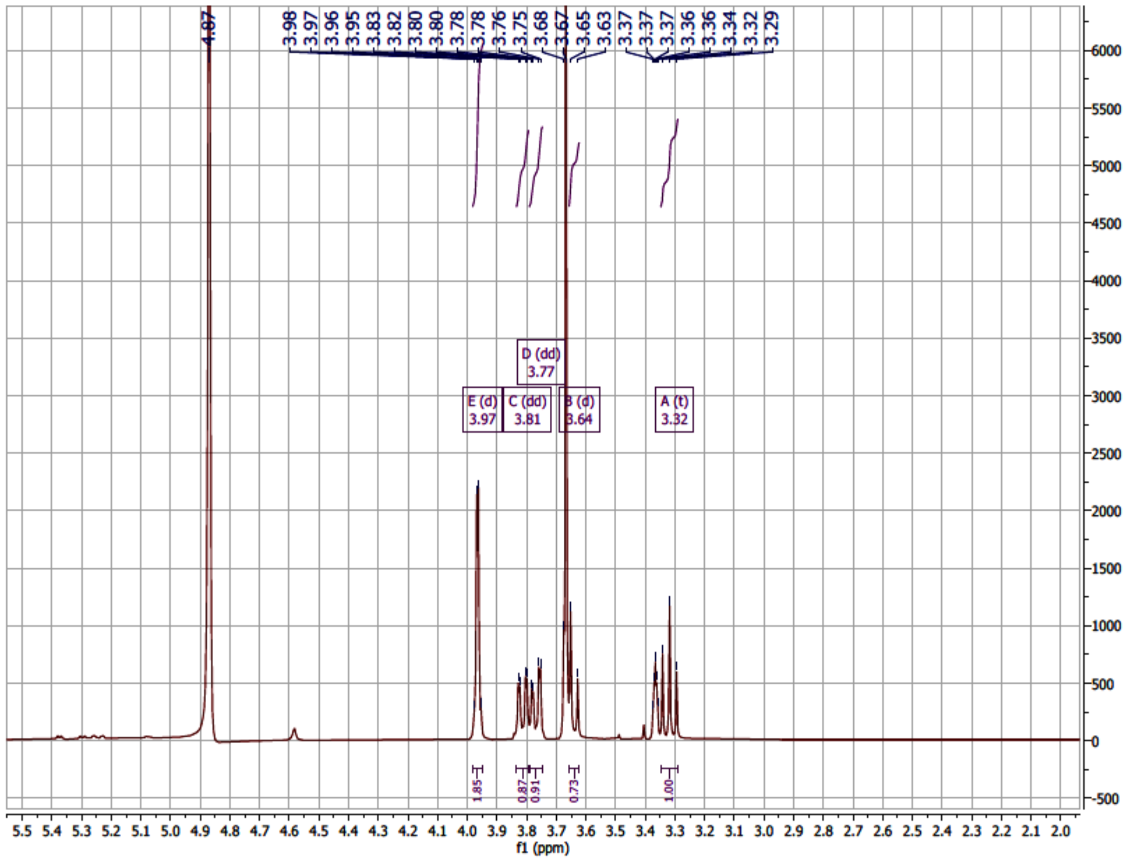

Supplementary figure S44: ^{1}H-NMR Spectrum of Compound **9** (400 MHz, CD3OD)

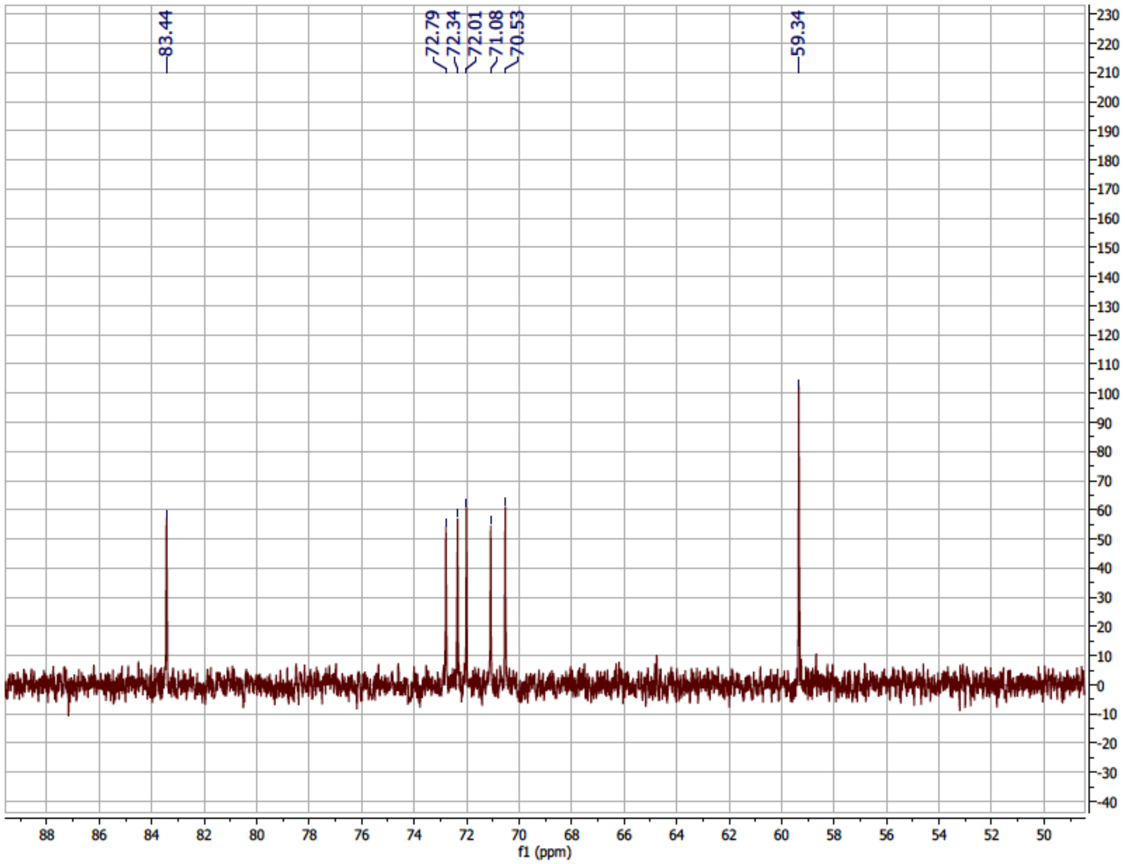

Supplementary figure S45: ^{13}C-NMR Spectrum of Compound **9** (100 MHz, CD3OD)

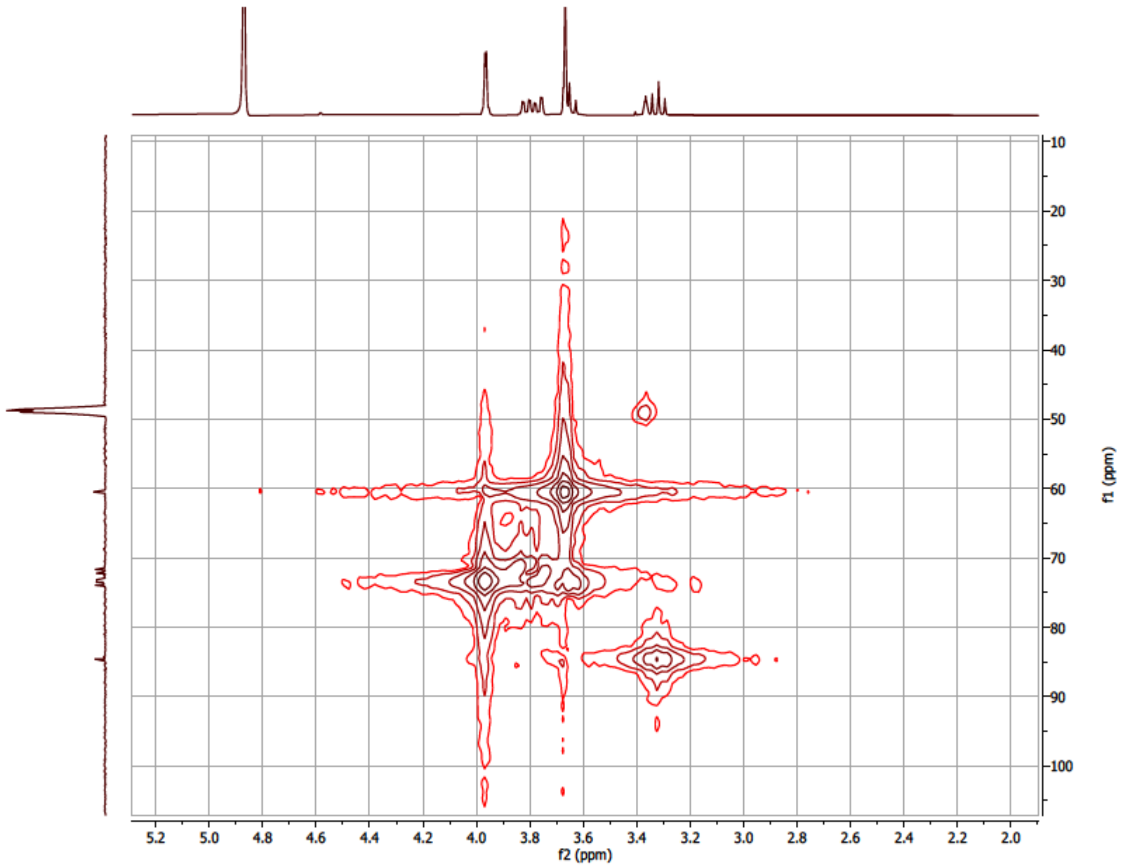

Supplementary figure S46: HMQC Spectrum of Compound **9** (400 MHz, CD3OD)

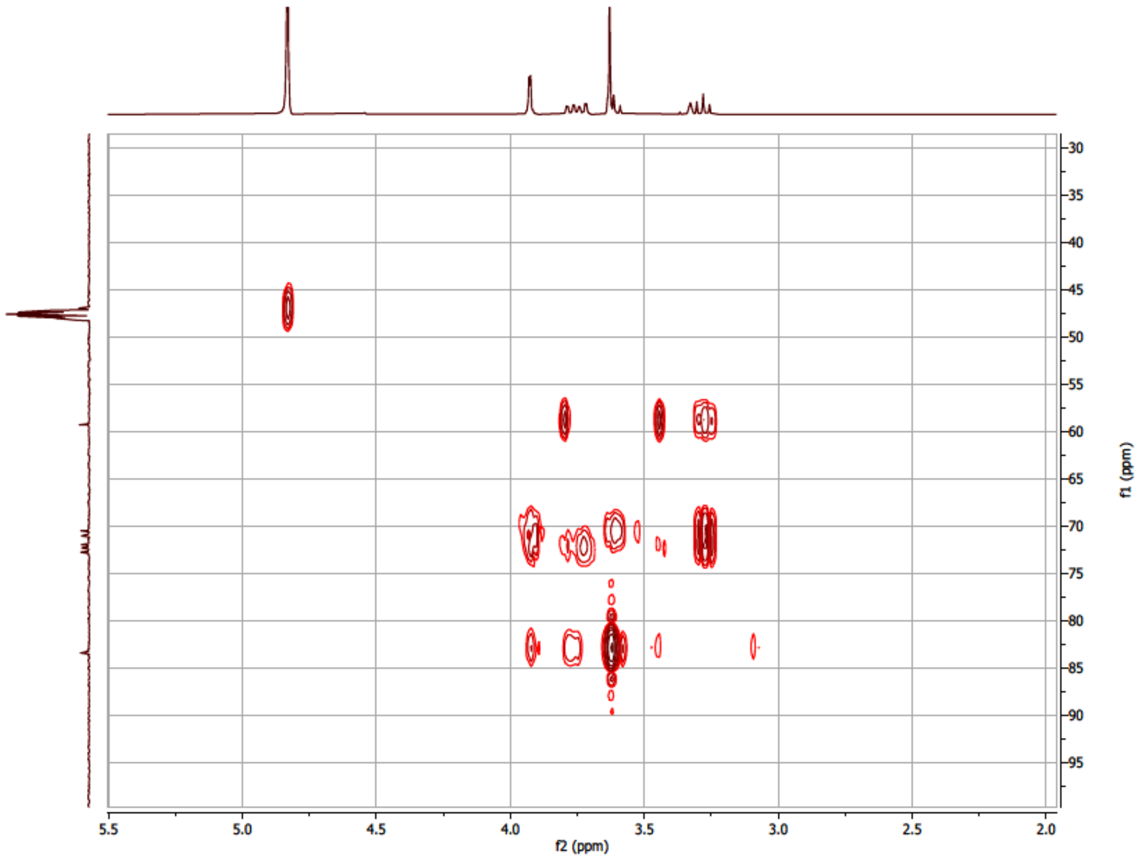

Supplementary figure S47: HMBC Spectrum of Compound **9** (400 MHz, CD3OD)

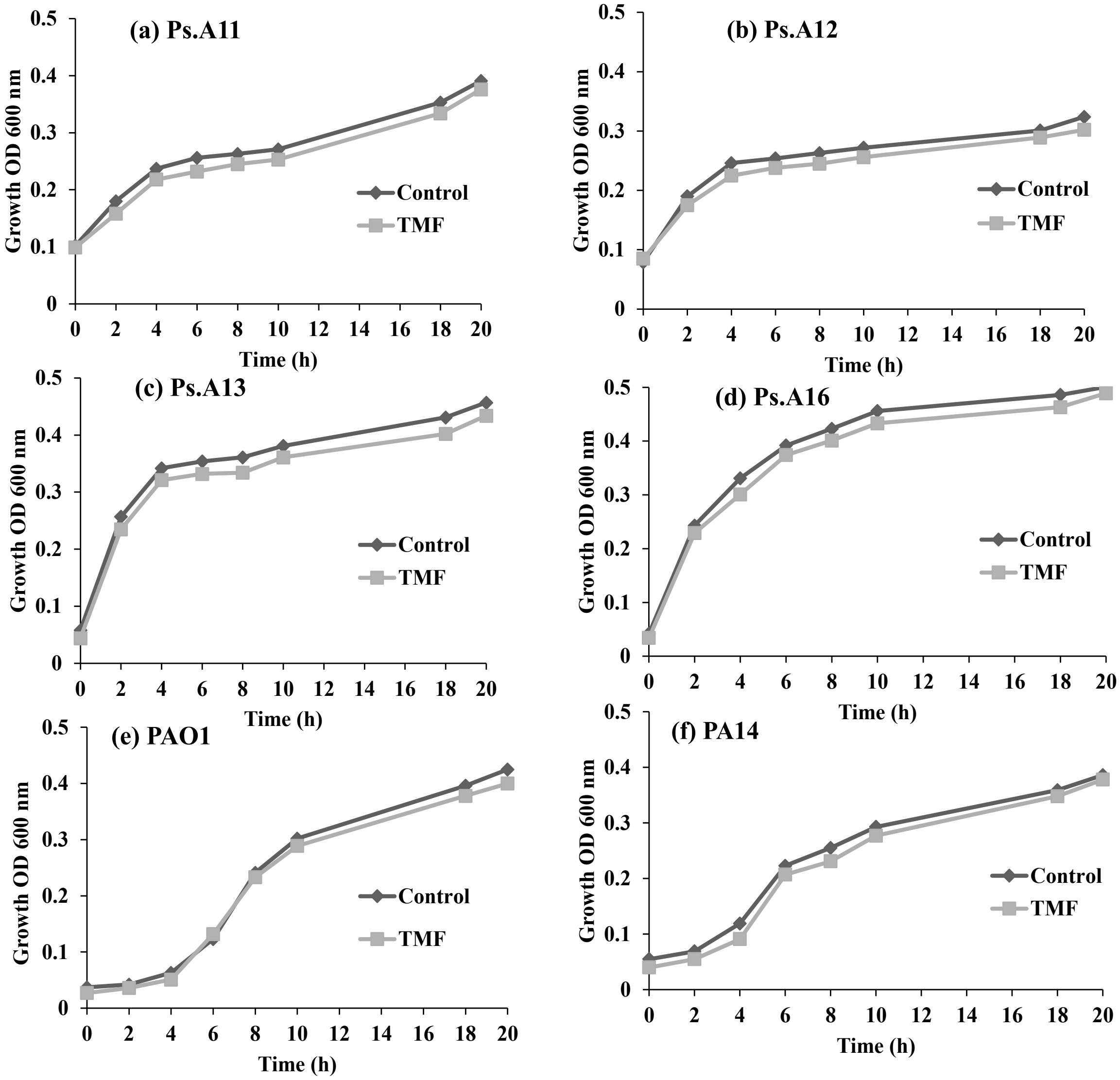

Supplementary figure S48: Growth curve of *P. aeruginosa* strains (a) Ps.A11, (b) Ps.A12, (c) Ps.A13, (d) Ps.A16, (e) PAO1, (f) PA14 in the presence and absence of 1/2 MIC of (3S,4R)-4,2`,4`-trihydroxy-7-methoxyisoflavan (TMF)

Supplementary table 1: Effect of 1/2 and 1/4 MIC of (3S,4R)-4,2`,4`-trihydroxy-7-methoxyisoflavan (TMF) on biofilm of *Pseudomonas aeruginosa* tested strains

Isolate number	OD 490 nm					
	Mean of untreated ± SD	SE	Mean of treated strains with 1/2 MIC ± SD	SE	Mean of treated strains with 1/4 MIC ± SD	SE
**Ps.A11**	1.45 ±0.030	0.0171	0.34 ±0.007	0.0040	0.33 ±0.022	0.0129
**Ps.A12**	1.12 ± 0.092	0.0531	0.28 ±0.018	0.0104	0.29 ±0.005	0.0026
**Ps.A13**	1.42 ±0.092	0.0529	0.29 ±0.012	0.0069	0.29 ±0.005	0.0026
**Ps.A16**	1.27 ±0093	0.0536	0.30 ±0.003	0.0015	0.32 ±0.009	0.0049
**PAO1**	1.32 ±0.030	0.0173	0.24 ±0.005	0.0027	0.30 ±0.003	0.0015
**PA14**	1.39 ±0.070	0.0404	0.26 ±0.010	0.0055	0.27 ±0.018	0.0101
**PAO-JP2**	0.04 ±0.004	0.0022	0.05 ±0.001	0.0007	0.04 ±0.004	0.0022

Supplementary table 2: Effect of 1/2 and 1/4 MIC of (3S,4R)-4,2`,4`-trihydroxy-7-methoxyisoflavan (TMF) on pyocyanin of *Pseudomonas aeruginosa* tested strains

Isolate number	Pyocyanin concentration (µg/ml)					
	Mean of untreated ± SD	SE	Mean of treated strains with 1/2 MIC ± SD	SE	Mean of treated strains with 1/4 MIC ± SD	SE
**Ps.A11**	15.70±0.047	0.0271	3.36±1.112	0.6418	3.50±1.272	0.7345
**Ps.A12**	11.95±0.547	0.3160	3.63±0.300	0.1734	3.79±0.267	0.1541
**Ps.A13**	12.42±0.473	0.2731	3.51±0.414	0.2389	3.31±0.240	0.1387
**Ps.A16**	13.42±0.244	0.1412	3.94±0.087	0.0502	4.63±0.053	0.0305
**PAO1**	13.67±0.913	0.5272	5.07±0.217	0.1251	5.26±0.245	0.1413
**PA14**	10.84±0.089	0.0512	4.23±0.091	0.0524	4.59±0.062	0.0358
**PAO-JP2**	0.78±0.022	0.0128	0.80±0.023	0.0131	0.79±0.023	0.0132

Supplementary table 3: Effect of 1/2 and 1/4 MIC of (3S,4R)-4,2`,4`-trihydroxy-7-methoxyisoflavan (TMF) on hemolysin of *Pseudomonas aeruginosa* strains

Isolate number	OD 540 nm					
	Mean of untreated ± SD	SE	Mean of treated strains with 1/2 MIC ± SD	SE	Mean of treated strains with 1/4 MIC ± SD	SE
**Ps.A11**	0.74±0.025	0.0145	0.34±0.015	0.0088	0.38±0.010	0.0058
**Ps.A12**	0.83±0.026	0.0153	0.38±0.031	0.0176	0.40±0.015	0.0088
**Ps.A13**	0.85±0.072	0.0416	0.50±0.012	0.0067	0.50±0.020	0.0115
**Ps.A16**	0.64±0.036	0.0208	0.31±0.006	0.0033	0.34±0.021	0.0120
**PAO1**	0.98±0.066	0.0379	0.37±0.055	0.0318	0.43±0.010	0.0058
**PA14**	0.97±0.010	0.0058	0.43±0.021	0.0120	0.44±0.035	0.0203
**PAO-JP2**	0.11±0.026	0.0153	0.10±0.052	0.0300	0.13±0.036	0.0208

Supplementary table 4: Effect of 1/2 and 1/4 MIC of (3S,4R)-4,2`,4`-trihydroxy-7-methoxyisoflavan (TMF) on protease of *Pseudomonas aeruginosa* strains

Isolate number	Reduction in OD 600 nm					
	Mean of untreated ± SD	SE	Mean of treated strains with 1/2 MIC ± SD	SE	Mean of treated strains with 1/4 MIC ± SD	SE
**Ps.A11**	0.87±0.015	0.0088	0.52±0.010	0.0058	0.50±0.015	0.0088
**Ps.A12**	0.79±0.021	0.0120	0.48±0.006	0.0033	0.49±0.010	0.0058
**Ps.A13**	0.98±0.017	0.0100	0.63±0.006	0.0033	0.64±0.012	0.0067
**Ps.A16**	0.85±0.015	0.0088	0.56±0.015	0.0088	0.59±0.010	0.0058
**PAO1**	0.91±0.015	0.0088	0.52±0.023	0.0133	0.54±0.025	0.0145
**PA14**	0.98±0.015	0.0088	0.63±0.021	0.0120	0.70±0.015	0.0088
**PAO-JP2**	0.04±0.065	0.0376	0.07±0.010	0.0058	0.02±0.031	0.0176
